# Inserting Three-Coordinate Nickel into [4Fe-4S] Clusters

**DOI:** 10.1021/acscentsci.4c00985

**Published:** 2024-10-03

**Authors:** Majed S. Fataftah, Daniel W. N. Wilson, Zachary Mathe, Theodore J. Gerard, Brandon Q. Mercado, Serena DeBeer, Patrick L. Holland

**Affiliations:** † Department of Chemistry, Yale University, New Haven, Connecticut 06520, United States; ‡ Max Planck Institute for Chemical Energy Conversion, Mülheim an der Ruhr 45470, Germany

## Abstract

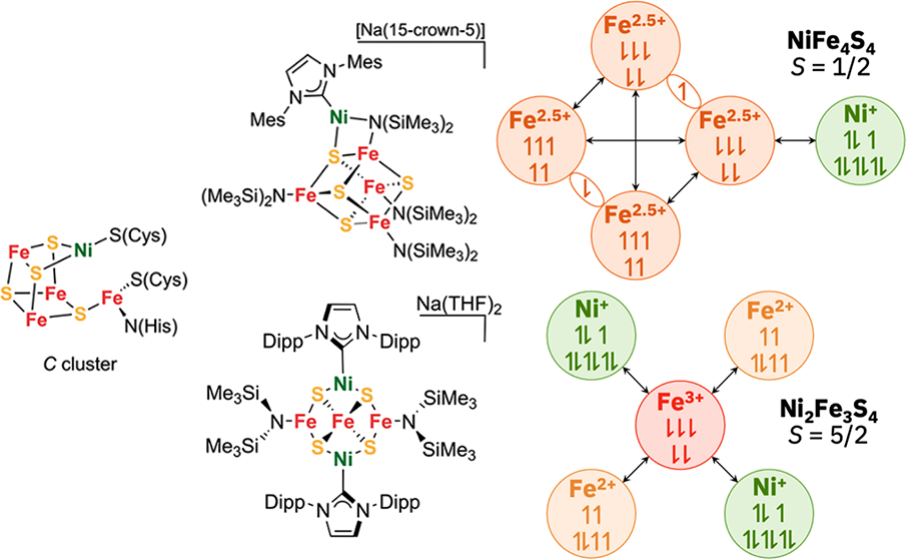

Metalloenzymes can efficiently achieve the multielectron interconversion of carbon dioxide and carbon monoxide under mild conditions. Anaerobic carbon monoxide dehydrogenase (CODH) performs these reactions at the *C* cluster, a unique nickel–iron–sulfide cluster that features an apparent three-coordinate nickel site. How nature assembles the [NiFe_3_S_4_]–Fe_u_ cluster is not well understood. We use synthetic clusters to demonstrate that electron transfer can drive insertion of a Ni^0^ precursor into an [Fe_4_S_4_]^3+^ cluster to assemble higher nuclearity nickel–iron–sulfide clusters with the same complement of metal ions as the *C* cluster. Initial electron transfer results in a [1Ni-4Fe-4S] cluster in which a Ni^1+^ ion sits outside of the cluster. Modifying the Ni^0^ precursor results in the insertion of two nickel atoms into the cluster, concomitant with ejection of an iron to yield an unprecedented [2Ni-3Fe-4S] cluster possessing four three-coordinate metal sites. Both clusters are characterized using magnetometry, electron paramagnetic resonance (EPR), Mössbauer, and X-ray absorption spectroscopy and supported by DFT computations that are consistent with both clusters having nickel in the +1 oxidation state. These results demonstrate that Ni^1+^ is a viable oxidation state within iron–sulfur clusters and that redox-driven transformations can give rise to higher nuclearity clusters of relevance to the CODH *C* cluster.

## Abstract 2

A nickel(0) complex inserts into an oxidized [4Fe-4S] cluster, giving iron−nickel−sulfur clusters that provide insight into the potential properties of the *C* cluster of CO dehydrogenase enzymes.

## Introduction

**Figure 1:**
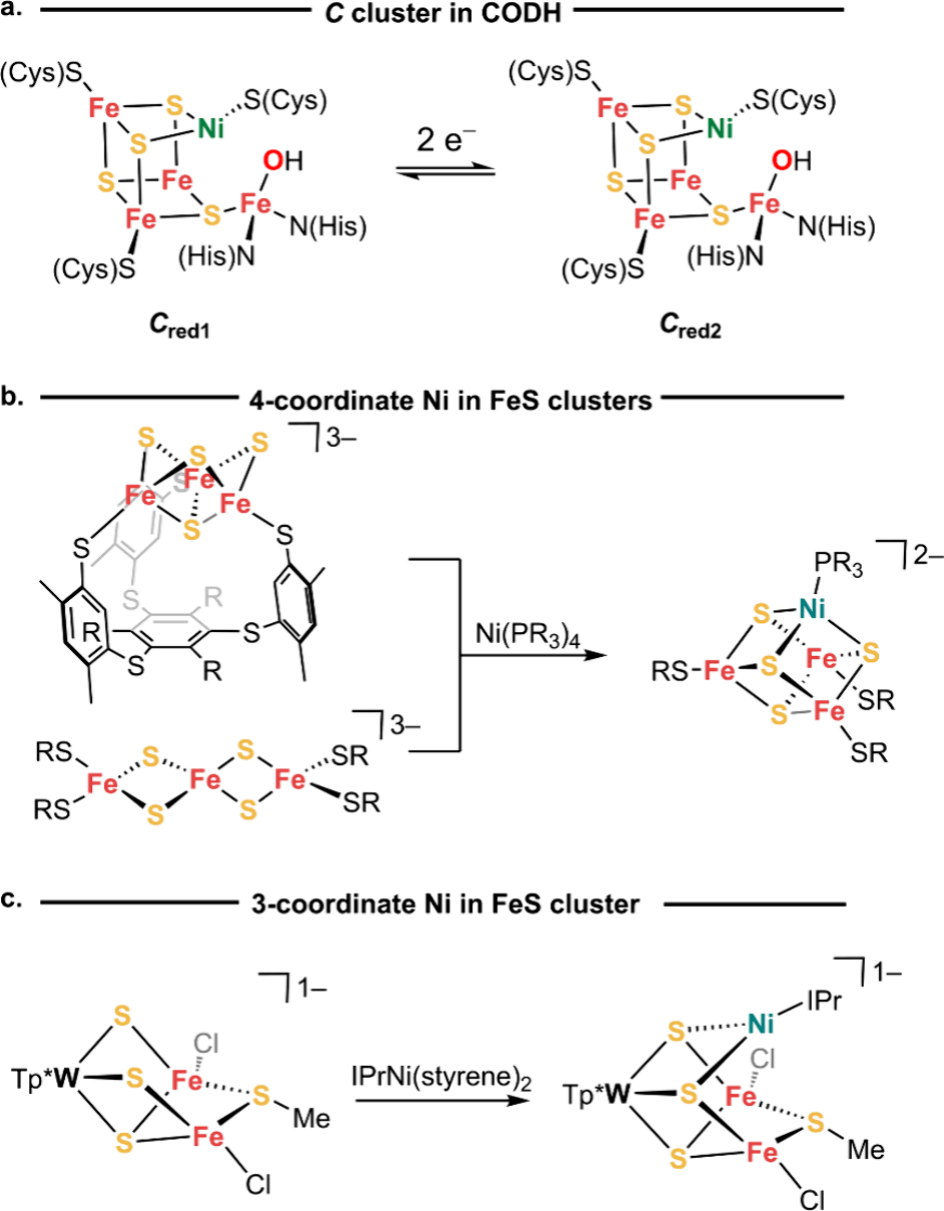
(a) Two catalytically relevant oxidation states of the *C* cluster in CODH that feature a three-coordinate nickel site. (b) Previously reported synthetic iron–sulfur clusters that contain four-coordinate nickel sites. (c) The first example of a three-coordinate nickel site in a synthetic iron–sulfur cluster. Tp* = tris(3,5-dimethyl-1-pyrazolyl)borate.

Nickel-containing carbon monoxide dehydrogenases (CODHs) catalyze the interconversion of CO_2_ and CO with high rates and vanishingly small overpotentials.^1−3^ Both directions of the reaction are important for biological functions.^4^ The oxidation of CO provides the energy required for the growth of anaerobic organisms, while the reduction of CO_2_ can be coupled with acetyl-coenzyme A synthetase (ACS) for the biosynthesis of the key metabolite acetyl-CoA.^5,6^ Crystallographic studies of anaerobic CODHs have revealed that the active site, known as the *C* cluster, is comprised of a unique heterometallic [NiFe_3_S_4_] open-cubane cluster attached to an additional iron site (Fe_u_) through a μ^3^-sulfide ligand (Figure 1a).^7,8^ The nickel site within the *C* cluster resides in a putative three-coordinate ligand environment and is the only crystallographically characterized example of a three-coordinate nickel site in biology. The reported crystallographic structures of the two active states of the *C* cluster, *C*_red1_, and its two-electron-reduced form, *C*_red2_, are nearly identical and display similar EPR spectra which leads to ambiguity in assigning metal oxidation states.^7^ Further, the absence of ^61^Ni coupling by ENDOR spectroscopy has led to the proposal that the Ni site has a closed shell configuration, as low-spin Ni^2+^ or Ni^0^.^5,9^ However, Ni^0^ is unprecedented in biological systems, especially with π-donating ligands such as the sulfides and thiolates found in the *C* cluster. The biosynthesis, catalytic mechanism, and structure–function relationships of this unusual cluster remain a topic of ongoing debate.

Iron–sulfur clusters exhibit compositional and structural diversity. A variety of cluster rearrangements have been identified in biological and synthetic systems, with redox state and ligand identity influencing the core structure of the cluster.^10,11^ Despite decades of synthetic studies on iron–sulfur clusters, synthetic strategies for incorporating heterometals such as nickel into cubane iron–sulfur clusters are limited and are summarized in Figure 1b.^11^ Three approaches have been used. The first involves using a multidentate ligand to construct a [Fe_3_S_4_] cluster that allows for addition of a heterometal, while the second approach involves a rearrangement of a linear [Fe_3_S_4_] cluster upon introduction of low-valent heterometals using precursors such as cobalt(0) and nickel(0) complexes.^12−16^ Both strategies have given access to [NiFe_3_S_4_] cubane clusters with four-coordinate nickel sites. In these synthetic clusters, the nickel sites have adopted either tetrahedral or square planar coordination geometries depending on the choice of mono- or bidentate supporting ligands on nickel. In contrast, when starting from a [Fe_4_S_4_] cluster, addition of a heterometal (M = Ni^2+^, Mo^0^) instead results in rhombic dodecahedral clusters ([M_2_Fe_6_S_6_]^2+/3+^).^17−19^ Alternatively, Holm and others have leveraged early transition metal tetrathiometalates (V, Mo, W) or metal trisulfides (e.g., [Tp*MS_3_]^−^, M = Mo or W) to template the stepwise addition of iron to assemble MFe_3_S_4_ clusters.^20,21^ We recently used this approach to isolate the first example of a three-coordinate nickel site in a [W-2Fe–Ni] cluster (Figure 1c).^22^ It remains unclear how clusters with structurally unusual features such as those found in the *C* cluster, which features a three-coordinate nickel site and dangling iron, may be assembled synthetically.

**Figure 2:**
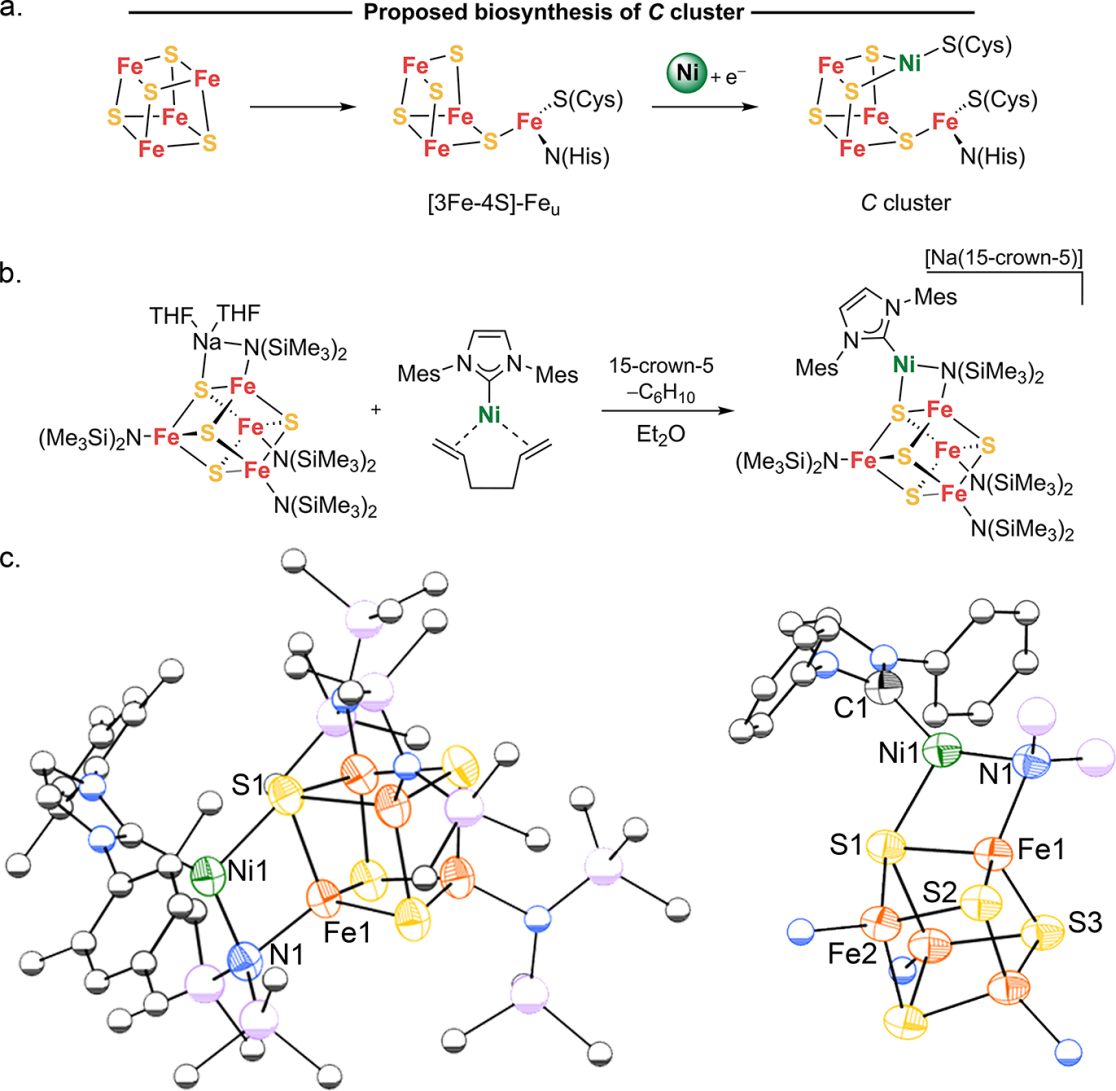
(a) Proposed biosynthesis of the *C* cluster. (b) Synthetic route to the **NiFe**_**4**_**S**_**4**_ cluster. (c) Molecular structure of **NiFe**_**4**_**S**_**4**_ as determined by single-crystal X-ray diffraction presented from two view perspectives. Orange, yellow, green, blue, pink and gray represent iron, sulfur, nickel, nitrogen, silicon, and carbon atoms, respectively. Anisotropic displacement ellipsoids depicted at 50% probability. The outer sphere cation, hydrogen atoms, and solvent molecules are omitted for clarity, and the right view additionally omits most silicon and some carbon substituents.

Herein, we demonstrate an additional strategy: treating an oxidized [Fe_4_S_4_]^3+^ cluster (formally Fe^3+^_3_Fe^2+^) with reduced Ni^0^ precursors, which leverages electron transfer to drive insertion of the nickel into the iron–sulfur cubane. This synthetic approach was inspired by the biosynthesis of the *C* cluster, which in the final step is proposed to involve a one-electron reduction of a [Fe_4_S_4_] cluster concomitant with nickel insertion into an incomplete cuboidal [Fe_3_S_4_]–Fe_u_ cluster (Figure 2a).^8,23,24^ Remarkably, the resulting heterometallic clusters exhibit unprecedented topologies for iron–sulfur clusters. In one instance, double insertion of the nickel into two edges of the [Fe_4_S_4_]^3+^ cubane ejects one Fe atom to give a [Ni_2_Fe_3_S_4_]^+^ cluster that features four three-coordinate metal sites. Modification of the steric protection around the nickel atom enables the isolation of a species in which the cubane remains intact and conjoined to a three-coordinate Ni^1+^ by a bridging amide and a cubane sulfide. The resulting [Ni–Fe–S] clusters are characterized using magnetic, spectroscopic, and computational methods to unequivocally identify that the nickel sites are in the +1 oxidation state. These results may guide future synthetic and biological studies of CODH enzymes. For instance, the disclosed strategy for synthesizing heterometallic clusters featuring low-coordinate metal sites could help target synthetic models that are topologically similar to the *C* cluster (i.e., models featuring a three-coordinate nickel and external iron site). Additionally, this work gives experimental precedent that three-coordinate Ni sites in iron–sulfur clusters are more consistent with Ni^1+^ rather than the initially proposed Ni^0^, aligning with computational studies which suggest that a Ni^0^ site would reduce the nearby iron centers.^25^

## Results

### Synthesis and Structural Characterization of Clusters

In order to drive the insertion of Ni into a [Fe_4_S_4_] cluster concomitant with one-electron reduction, we selected low-oxidation state nickel(0) precursors supported by a *N*-heterocyclic carbene ligand along with labile alkene/arene ligands.^26,27^ We hypothesized that the *redox mismatch* between this reduced nickel precursor and highly oxidized [Fe_4_S_4_]^3+^ clusters would enable insertion of Ni into the [Fe_4_S_4_] cluster using the electron transfer as a driving force. For the iron–sulfur precursors, we chose the series of clusters from Lee and Ohki that are stabilized by amide ligands (N(SiMe_3_)_2_).^28,29^

The stoichiometric reaction between IMesNi(1,5-hexadiene) (IMes = 1,3-(bis-2,4,6-trimethylphenyl)-1,3-dihydro-2*H*-imidazol-2-ylidene) and Na(THF)_2_Fe_4_S_4_(N(SiMe_3_)_2_)_4_ (**[Fe**_**4**_**S**_**4**_**]**^**3+**^) in diethyl ether followed by the addition of 15-crown-5 affords [Na(15-crown-5)Et_2_O][IMesNiFe_4_S_4_(N(SiMe_3_)_2_)_4_] (**NiFe**_**4**_**S**_**4**_) in isolated yields around 60% (Figure 2b). The ^1^H NMR spectrum of **1** in C_6_D_6_ displays nine paramagnetically broadened resonances between δ 1.0 and 9.0 ppm. The X-ray crystal structure of **1** reveals that the IMesNi moiety is bound to the exterior of the [4Fe–4S] cubane via a μ_2_-N(SiMe_3_)_2_ ligand and a μ_4_-sulfide (Figure 2c). The Ni site in **NiFe**_**4**_**S**_**4**_ has a trigonal planar geometry (sum of angles = 359.95(2)°) with a Ni–S1 distance of 2.257(1) Å and a Ni–C distance of 1.901(5) Å. The geometry about the Ni site is distorted toward T-shaped with a N–Ni–C angle of 143.9(2)°. The bond distances to the μ_4_-sulfide (S1) in **NiFe**_**4**_**S**_**4**_ are elongated by 0.06–0.07 Å relative to the μ_3_-sulfides in **[Fe**_**4**_**S**_**4**_**]**^**3+**^ (Fe2–S1 2.377(1) Å and Fe1–S1 2.357(2) Å). The molecular structure of the cubane portion of **NiFe**_**4**_**S**_**4**_ is otherwise similar to that of the **[Fe**_**4**_**S**_**4**_**]**^**3+**^ precursor, which enables a comparison of their cubane core volumes for insight into the cubane redox states.^30^ Analysis of the structural metrics of **NiFe**_**4**_**S**_**4**_ shows expansion of the S_4_ tetrahedron volume from 5.23(1) Å^3^ in **[Fe**_**4**_**S**_**4**_**]**^**3+**^ to 5.44(2) Å^3^ in **NiFe**_**4**_**S**_**4**_, while the Fe_4_ tetrahedron volume remains unchanged (2.76(1) Å^3^ in **[Fe**_**4**_**S**_**4**_**]**^**3+**^ versus 2.74(2) Å^3^ in **NiFe**_**4**_**S**_**4**_). The changes in the S_4_ core volume suggest a one-electron reduction of the [4Fe–4S] core in **1**, which is consistent with a literature analysis of a redox series of [Fe_4_S_4_]^*n*^ clusters (*n* = 0–4).^31^

**Figure 3:**
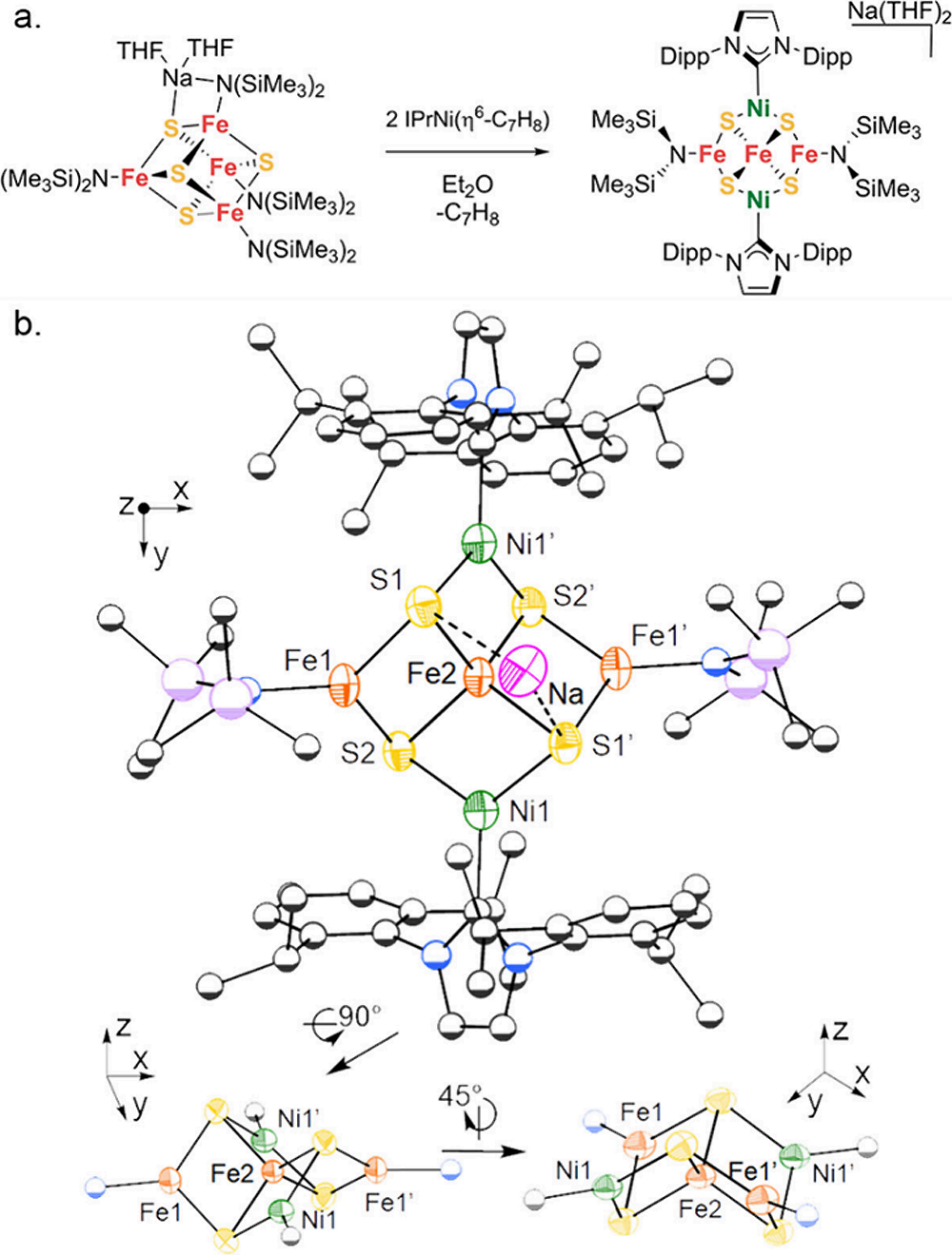
(a) Synthetic route to the **Ni**_**2**_**Fe**_**3**_**S**_**4**_ cluster. (b) Molecular structures of **Ni**_**2**_**Fe**_**3**_**S**_**4**_ as determined by single-crystal X-ray diffraction. Orange, yellow, green, blue, gray, and pink represent iron, sulfur, nickel, nitrogen, carbon, and sodium atoms, respectively. Anisotropic displacement ellipsoids depicted at 50% probability. The 2,6-diisopropylphenyl and N(SiMe_3_)_2_ moieties are represented as spheres of arbitrary radius, and hydrogen atoms and solvent molecules are omitted for clarity.

Addition of the more sterically hindered precursor IPrNi(1,5-hexadiene) (IPr = 1,3-(bis-2,6-diisopropylphenyl)-1,3-dihydro-2*H*-imidazol-2-ylidene) to **[Fe**_**4**_**S**_**4**_**]**^**3+**^ leads to the formation of a new product by ^1^H NMR and EPR spectroscopy. Unfortunately, we were unable to crystallographically characterize the product. It is notable, however, that EPR and Mössbauer spectra of the reaction products are similar to those of **NiFe**_**4**_**S**_**4**_, suggesting that at least one of the products could be structurally similar to **NiFe**_**4**_**S**_**4**_ (see SI for detailed discussion). Changing to a different nickel precursor with a more labile leaving group, IPrNi(η^6^-C_7_H_8_), enables the conversion of **[Fe**_**4**_**S**_**4**_**]**^**3+**^ to a product with a more extensive rearrangement of the cluster core (Figure 3a). Addition of 2 equiv of IPrNi(η^6^-C_7_H_8_) to **[Fe**_**4**_**S**_**4**_**]**^**3+**^ gives a product **Ni**_**2**_**Fe**_**3**_**S**_**4**_ in 40–60% yield. Its ^1^H NMR spectrum shows several paramagnetically shifted resonances between −30 and +12 ppm. The molecular structure of **Ni**_**2**_**Fe**_**3**_**S**_**4**_ was identified by X-ray crystallography as [Na(THF)_2_](IPrNi)_2_Fe_3_S_4_(N(SiMe_3_)_2_)_2_ (Figure 3b). Surprisingly, **Ni**_**2**_**Fe**_**3**_**S**_**4**_ has three-coordinate metals in four of its five metal sites. Overall, the topology of **Ni**_**2**_**Fe**_**3**_**S**_**4**_ can be described as two intersecting M_3_S_4_ linear chains with a central tetrahedral iron site (Fe2), two peripheral three-coordinate iron sites (Fe1), and two peripheral three-coordinate nickel sites, where each pair of peripheral metals is crystallographically equivalent. The Fe2–S distances are 2.283(2) and 2.231(2) Å, and the central Fe has a τ_4_ parameter of 0.94 that indicates a near-tetrahedral coordination environment. The geometry about each Fe1 site is trigonal planar (sum of angles = 359.9(2)°), the Fe1–S distances are 2.267(2) and 2.252(2) Å, and the Fe1–N distance is 1.908(6) Å. Similarly, the geometry about each Ni is trigonal planar (sum of angles = 359.8(2)°) with Ni–S distances of 2.182(2) Å and a Ni–C distance of 1.899(8) Å. The bond parameters for **Ni**_**2**_**Fe**_**3**_**S**_**4**_ can be compared to those reported for [IPrNi(μ_2_-SH)]_2_ and [IPrNi(μ_2_-S)]_2_, which feature formally Ni^1+^ and Ni^2+^ sites, respectively. The Ni–C and Ni–S distances in **Ni**_**2**_**Fe**_**3**_**S**_**4**_ are closest to those in [IPrNi(μ_2_-SH)]_2_ (Ni–C 1.87 Å and average Ni–S 2.20 Å).^32^ Though the similarity of these Ni–(μ_2_-SH) and Ni–(μ_3_-S) cores is limited, a more direct comparison can be made with a recently reported [W–2Fe–Ni] cluster which has a nickel site most consistent with nickel(I).^22^ It has nickel–sulfide distances of 2.1681(8) and 2.1775(8) Å, which are very similar to those seen in **Ni**_**2**_**Fe**_**3**_**S**_**4**_, suggesting that the sites in **Ni**_**2**_**Fe**_**3**_**S**_**4**_ are also nickel(I), an idea that is tested with spectroscopy below.

### Redox and Ligand Properties

We used cyclic voltammetry (CV) in 0.1 M [Bu_4_N][PF_6_]/THF to explore the redox properties of **NiFe**_**4**_**S**_**4**_. The first oxidation at *E* = −0.75 V (vs Fc/Fc^+^) is quasi-reversible with a nonlinear dependence of the peak current on the square root of the scan rate. The potential may be compared to that of the [Fe_4_S_4_]^3+/2+^ redox couple of the four-iron analogue **NaFe**_**4**_**S**_**4**_ with a simple Na^+^ cation, which lies at −1.06 V.^31,33^ The shift of almost 0.3 V suggests that the nickel is not redox innocent during the process. The identification of this oxidation product as well as the result of the irreversible second oxidation at −0.08 V is ongoing.

In addition to the difference in topology, there are some differences between the ligand environment of **NiFe**_**4**_**S**_**4**_ and the *C* cluster that are important to notice in this context. First, the amide donors in **NiFe**_**4**_**S**_**4**_ strongly impact the redox potentials relative to the thiolate and histidine ligands in the natural system. In a head-to-head comparison of synthetic systems, the redox potentials of thiolate-bound Fe_4_S_4_ clusters are nearly 350 mV more anodic than those in **NaFe**_**4**_**S**_**4**_.^29^ The amide ligands stabilize higher oxidation states of the cluster and may have other influences on the electronic structures of our compounds. This effect is the topic of ongoing research. Second, NHC ligands are coordinated to the nickel sites in **NiFe**_**4**_**S**_**4**_ and **Ni**_**2**_**Fe**_**3**_**S**_**4**_, contrasting the thiolate/thiol and histidine ligands found at the *C* cluster. NHCs are neutral ligands that are strong σ-donors and π-acceptors, and they stabilize lower cluster oxidation states than thiol/thiolate ligands. For example, NHC-coordinated Fe_4_S_4_ clusters can be isolated as the all-ferrous [Fe_4_S_4_]^0^,^34^ whereas thiolate-coordinated all-ferrous Fe_4_S_4_ clusters have only been isolated by using closely associated potassium cations to stabilize the negative charge.^31,33^ Though NHC ligands deviate from the biological coordination environment, they provide kinetic protection and electronic stabilization of the clusters, which makes them valuable in many bioinorganic model complexes.^35−40^

### Spin States from Spectroscopy and Magnetometry

**Figure 4:**
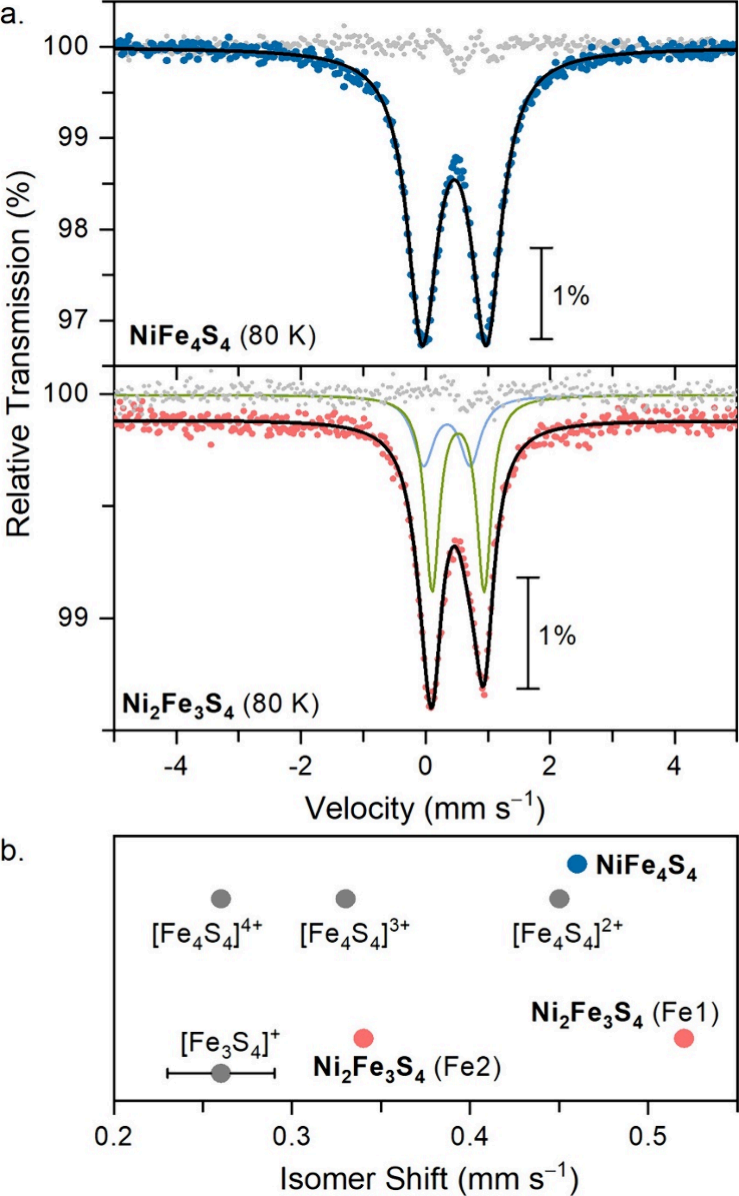
(a) Stacked Mössbauer spectra of **NiFe**_**4**_**S**_**4**_ and **Ni**_**2**_**Fe**_**3**_**S**_**4**_ at 80 K. **NiFe**_**4**_**S**_**4**_: δ = 0.46 mm s^–1^ (δ_DFT_ = 0.44 mm s^–1^), |Δ*E*_Q_| = 1.03 mm s^–1^, Γ = 0.56 mm s^–1^. **Ni**_**2**_**Fe**_**3**_**S**_**4**_, site 1 (green, 67%): δ_1_ = 0.49 mm s^–1^ (δ_1,DFT_ = 0.39 mm s^–1^), |Δ*E*_Q_|_1_ = 0.75 mm s^–1^, Γ_1_ = 0.36 mm s^–1^; site 2 (blue, 33%): δ_2_ = 0.34 mm s^–1^ (δ_2,DFT_ = 0.35 mm s^–1^), |Δ*E*_Q_|_2_ = 0.83 mm s^–1^, Γ_2_ = 0.28 mm s^–1^. (b) Experimental δ values for **NiFe**_**4**_**S**_**4**_ and **Ni**_**2**_**Fe**_**3**_**S**_**4**_ relative to structurally analogous amide-supported Fe_4_S_4_ clusters and to thiolate-supported linear chain Fe_3_S_4_ clusters from the literature.^29,41^

Mössbauer spectroscopy is a powerful tool to assess iron oxidation states in iron–sulfur clusters. The zero-field Mössbauer spectrum of **NiFe**_**4**_**S**_**4**_ was collected at 80 K (Figure 4a, upper spectrum) and reveals one quadrupole doublet with an isomer shift (δ) of 0.46 mm s^–1^ and quadrupole splitting (Δ*E*_Q_) of 1.03 mm s^–1^. Comparing the δ value for **NiFe**_**4**_**S**_**4**_ relative to the structurally analogous redox series [Fe_4_S_4_(N(SiMe_3_)_2_]^0,1–,2–^ shows an increase in the isomer shift value relative to the **[Fe**_**4**_**S**_**4**_**]**^**3+**^ precursor (δ_avg_ = 0.32 mm s^–1^). Instead, the isomer shift is most similar to the one in [**Fe**_**4**_**S**_**4**_]^2+^ (δ = 0.45 mm s^–1^), which has two mixed-valent Fe^2.5+^_2_ pairs (Figure 4b). Thus, the Mössbauer data of **NiFe**_**4**_**S**_**4**_ are consistent with one-electron transfer from the [IMesNi^0^] moiety to the [Fe_4_S_4_]^3+^ cluster. This suggests that the metal oxidation states in **NiFe**_**4**_**S**_**4**_ are 4Fe^2.5+^ and Ni^1+^, consistent with the core volume analysis presented above. Notably, addition of *tert*-butyl isocyanide to **NiFe**_**4**_**S**_**4**_ regenerates the [Fe_4_S_4_]^3+^ cluster and generates the nickel(0) species IMesNi(*t*BuNC)_3_, showing that the electron can be transferred back to nickel (Figures S5 and S6).

We collected the zero-field Mössbauer spectrum of **Ni**_**2**_**Fe**_**3**_**S**_**4**_ at 80 K to assess the iron oxidation states and to provide indirect evidence of the Ni oxidation states. The Mössbauer spectrum of **Ni**_**2**_**Fe**_**3**_**S**_**4**_ shows one apparent quadrupole doublet centered at δ = 0.49 mm s^–1^ (Figure 4a, lower spectrum). Since **Ni**_**2**_**Fe**_**3**_**S**_**4**_ features two unique iron sites that have different coordination numbers and ligands, the Mössbauer spectrum was fitted to a model with two sites in a 1:2 ratio, which gave δ_1_ = 0.34 mm s^–1^ (33%) and δ_2_ = 0.52 mm s^–1^ (67%). The closest structural analogues to **Ni**_**2**_**Fe**_**3**_**S**_**4**_ are the [Fe_3_S_4_(SR)_4_]^3–^ linear chain clusters whose Mössbauer spectra gave δ values in the range of 0.23–0.29 mm s^–1^ and are representative of four-coordinate Fe^3+^ sites with thiolate ligands.^41,42^ This comparison is consistent with **Ni**_**2**_**Fe**_**3**_**S**_**4**_ having one Fe^3+^ and two Fe^2+^ sites. However, the poor spectral resolution of the two sites by Mössbauer spectroscopy compels further evidence from magnetometry, X-ray absorption spectroscopy, and broken-symmetry density functional theory calculations.

**Figure 5:**
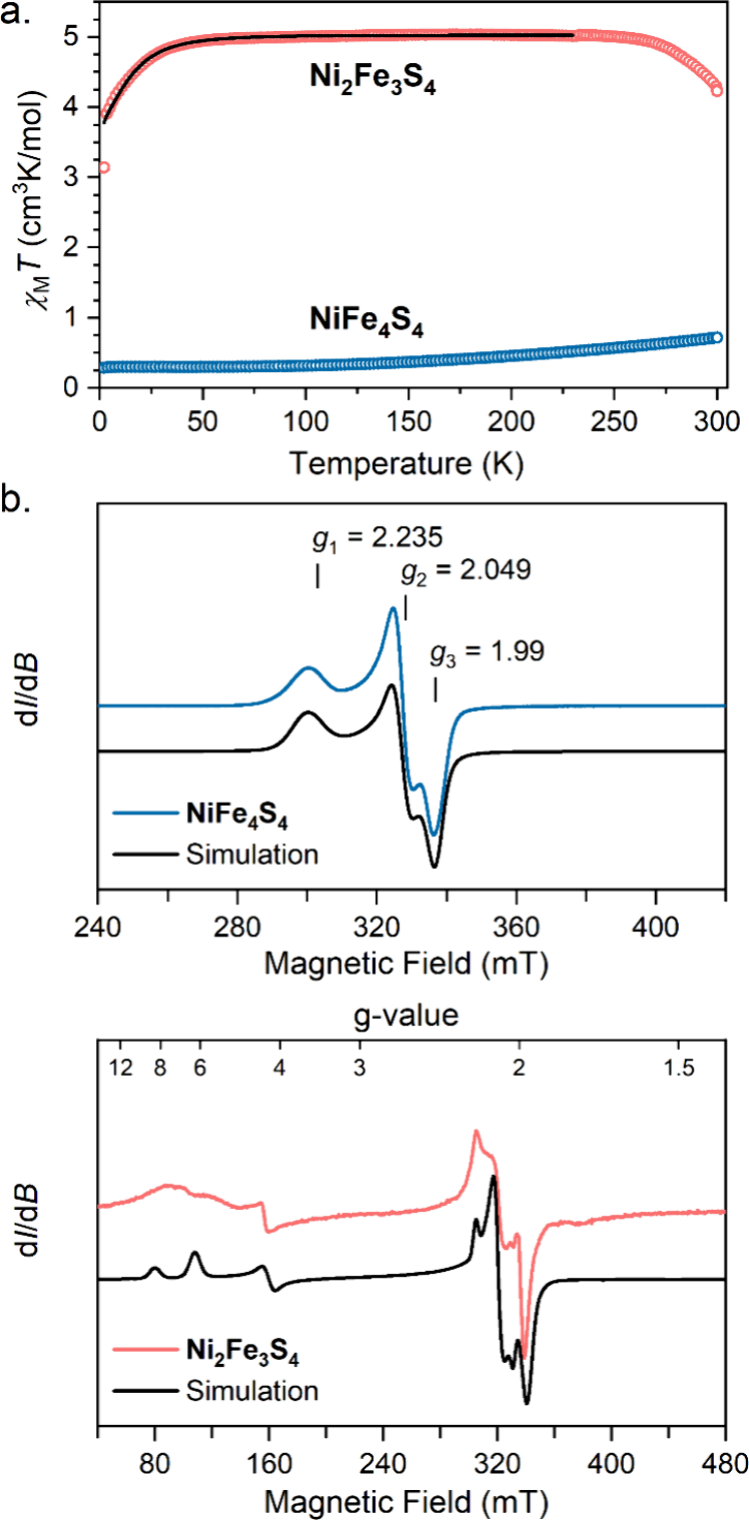
(a) Overlay of the dc magnetic susceptibility data for **NiFe**_**4**_**S**_**4**_ (blue) and **Ni**_**2**_**Fe**_**3**_**S**_**4**_ (red) collected under an applied magnetic field of 0.1 T. (b) EPR spectrum of a frozen solution of **NiFe**_**4**_**S**_**4**_ in toluene (2 mM) collected at 9.38 GHz and 5 K. (c) EPR spectrum of a solid sample of **Ni**_**2**_**Fe**_**3**_**S**_**4**_ collected at 9.38 GHz and 10 K.

To inspect the spin ground state in **NiFe**_**4**_**S**_**4**_ and **Ni**_**2**_**Fe**_**3**_**S**_**4**_ we measured their dc magnetic susceptibilities between 2 and 300 K under an applied magnetic field of 0.1 T. The χ_M_*T* value for **NiFe**_**4**_**S**_**4**_ at 2 K is 0.38 cm^3^·K/mol, which indicates a *S* = 1/2 ground state. The χ_M_*T* value steadily increases at higher temperature, suggesting that there are low-lying excited states with higher spin (Figure 5a). To corroborate our magnetic analysis, **NiFe**_**4**_**S**_**4**_ was characterized by EPR spectroscopy (Figure 5b). The X-band EPR spectrum of **NiFe**_**4**_**S**_**4**_ was collected in frozen toluene at 5 K and reveals a rhombic spectrum that was simulated using EasySpin^43^ and the spin Hamiltonian *Ĥ* = (*g*_*x*_ + *g*_*y*_ + *g*_*z*_)μ_B_***SH***, where *g*_*x*,*y*,*z*_ are the principal *g* values. Our best simulation gave *g* values of 2.23, 2.05, and 1.99. The large *g*_*x*_ value of 2.23 supports significant participation of the nickel site in the spin system, as such large *g* values are uncommon for [4Fe-4S] clusters but are typical for *S* = 1/2 Ni^1+^ compounds.^44,45^ However, the low *g*_*z*_ value of 1.99 suggests that some spin density lies on the [4Fe-4S] component, as *S* = 1/2 nickel species do not give *g* values < 2.^46^

Next, we probed **Ni**_**2**_**Fe**_**3**_**S**_**4**_ by SQUID magnetometry to assess possible spin coupling schemes that would provide insight into the metal oxidation states and their electronic configurations. We measured the dc susceptibility of **Ni**_**2**_**Fe**_**3**_**S**_**4**_ from 2 to 300 K under an applied magnetic field of 0.1 T (Figure 5a). **Ni**_**2**_**Fe**_**3**_**S**_**4**_ exhibits a χ_M_*T* value of 5.05 cm^3^·K/mol in the temperature range of 30–250 K, close to the theoretically expected value for a *S* = 5/2 ground state (χ_M_*T* = 4.875 for isotropic *S* = 5/2). The χ_M_*T* data were simulated using the Van Vleck equation using the spin Hamiltonian *Ĥ* = *DŜ*_*z*_^2^ + *E*(*Ŝ*_*x*_^2^ – *Ŝ*_*y*_^2^) + (*g*_*x*_ + *g*_*y*_ + *g*_*z*_)μ_B_***SH***, where *D* and *E* are the axial and transverse zero-field splitting parameters, *S*_*x*,*y*,*z*_ are the spin operators, and *g*_*x*,*y*,*z*_ are the principal *g* values. The best simulation to the data yielded the following parameters for **Ni**_**2**_**Fe**_**3**_**S**_**4**_: *g*_*x*,*y*_ = 2.12, *g*_*z*_ = 2.18, and *D* = −9.0 ± 0.5 cm^–1^, while *E* was held at zero. We corroborated our spin Hamiltonian parameters using EPR spectroscopy. The X-band EPR spectrum of **Ni**_**2**_**Fe**_**3**_**S**_**4**_ at 10 K shows resonances in the range from 50 to 350 mT, characteristic of a high-spin ground state (Figure 5c). The EPR spectrum was simulated using the following parameters: *S* = 5/2, *g*_*x,y,z*_ = [2.11 2.15 2.15], *D* = −9.0 cm^–1^, and *E*/*D* = 0.09. These spin Hamiltonian parameters are in good agreement with those extracted from fitting the dc magnetic susceptibility data. The *D* value is unusually large for a [4Fe-4S] cluster. Previously reported |*D|* values for heterometal-substituted ferredoxins are in the range of 1.5–3 cm^–1^.^47^ However, the three-coordinate Fe sites in **Ni**_**2**_**Fe**_**3**_**S**_**4**_ likely enhance the magnetic anisotropy of the ground state of **Ni**_**2**_**Fe**_**3**_**S**_**4**_ relative to four-coordinate Fe sites typically found in [4Fe-4S] clusters. We recently reported *D* values in the range of 35–40 cm^–1^ for β-diketiminate-supported Fe^2+^ compounds.^48^ Thus, spin projection of the three-coordinate Fe^2+^ sites on the total ground state is likely the primary contributor to the large *D* value for **Ni**_**2**_**Fe**_**3**_**S**_**4**_ relative to [4Fe-4S] clusters that feature higher coordinate iron sites.^49^ Because **Ni**_**2**_**Fe**_**3**_**S**_**4**_ has a thermally persistent *S* = 5/2 ground state, the magnitudes of the antiferromagnetic exchange energies are too large to be determined nor do these data specify the metal oxidation states. The origin of the downturn in χ_M_*T* above 250 K (Figure 5a) remains unclear as collecting data above 300 K led to sample degradation (see SI for additional discussion).

The *S* = 5/2 ground state of **Ni**_**2**_**Fe**_**3**_**S**_4_ could in principle be rationalized using antiferromagnetic coupling between a central Fe^3+^_,_ two outside Fe^3+^ sites, and two closed-shell Ni^0^ sites. However, this interpretation is inconsistent with the Mössbauer and structural data above. The more reasonable spin coupling scheme has a central high-spin Fe^3+^ antiferromagnetically coupled to two high-spin Fe^2+^ and two Ni^1+^ sites. To test this spin coupling model, we pursued X-ray absorption spectroscopy (XAS) and broken-symmetry density functional theory (BS DFT) calculations on **NiFe**_**4**_**S**_**4**_ and **Ni**_**2**_**Fe**_**3**_**S**_**4**_.

### X-ray Absorption Spectroscopy and DFT Computations

**Figure 6:**
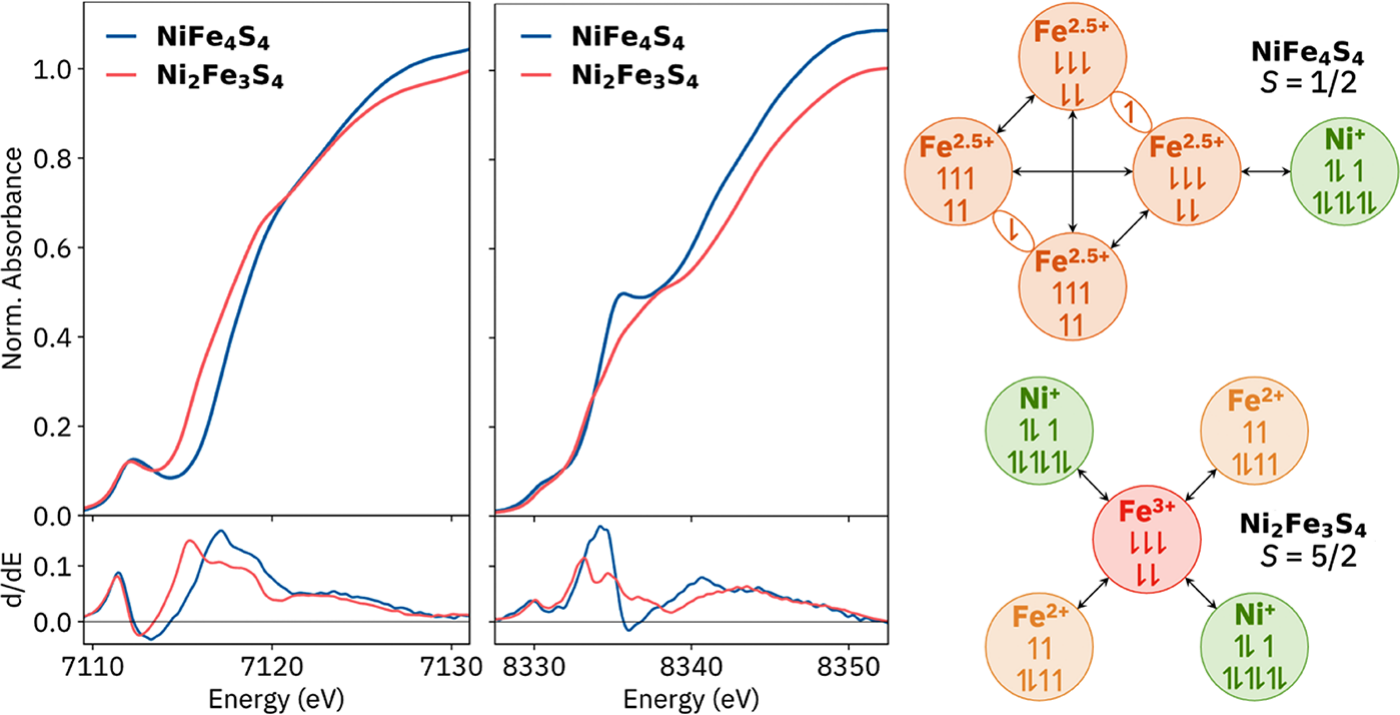
(a) X-ray absorption spectra at the iron and nickel K-edges, with derivative spectra below. In the Fe XAS of **NiFe**_**4**_**S**_**4**_ and **Ni**_**2**_**Fe**_**3**_**S**_**4**_, the pre-edges are found at 7112.2 and 7112.4 eV, while the first-derivative maxima are found at 7116.9 and 7115.6 eV, respectively. (b) Spin coupling schemes for **NiFe**_**4**_**S**_**4**_ (top) and **Ni**_**2**_**Fe**_**3**_**S**_**4**_ (bottom); arrows represent dominantly antiferromagnetic exchange interactions.

The X-ray absorption spectra at the iron and nickel K-edges are presented in Figure 6 together with schematic representations of the spin coupling within the clusters. The iron K-edge energy of **NiFe**_**4**_**S**_**4**_ is typical for mixed-valent (Fe^2.5+^)_4_S_4_ systems, which are remarkably consistent across chemical environments.^50^ For **Ni**_**2**_**Fe**_**3**_**S**_**4**_, the early edge (7114–7117 eV) has very high intensity, and despite the trigonal-planar Fe coordination, a pronounced 4p_*z*_ feature is not observed.^51^ These observations indicate the presence of many low-lying metal–metal charge transfer (MMCT) states and significant mixing of the metal 3d orbitals (see Figure S41). Although local 3d transitions for the central Fe^3+^ are calculated to be ∼0.5 eV higher in energy than those of Fe^2+^ sites and support the local oxidation state assignments, separate pre-edge peaks are not experimentally resolvable (see Figure S43).

Both nickel spectra include pre-edge features at 8330 eV, indicating significant depopulation of the Ni 3d orbitals and consistent with Ni^1+^ local oxidation states. In Ni K-edge time-dependent DFT calculations (TPSSh+ZORA/ZORA-def2-TZVP, performed with ORCA 5;^52^ see SI for computational details), the first excited states are beta-only with primarily local Ni 3d character, further supporting Ni^1+^ assignments (see SI). For **Ni**_**2**_**Fe**_**3**_**S**_**4**_, an intense MMCT region (8331–8334 eV) is observed, similar to that observed in the Fe edge. The sharp Ni 4p_*z*_ feature at 8335 eV for **NiFe**_**4**_**S**_**4**_ is typical for planar Ni species;^53−55^ it is less pronounced in **Ni**_**2**_**Fe**_**3**_**S**_**4**_ because the approximately coplanar NHC and Ni coordination planes allow for mixing of the Ni 4p_*z*_ orbital with NHC π* orbitals (see SI).^56^

Localized orbital analysis with BS DFT supports the above oxidation state assignments for **NiFe**_**4**_**S**_**4**_ and **Ni**_**2**_**Fe**_**3**_**S**_**4**_. For both clusters, SOMOs with predominant Ni 3d character were found (see Figures S33 and S35). **NiFe**_**4**_**S**_**4**_ has a typical mixed-valent (Fe^2.5+^)_4_S_4_ subsystem with the unpaired 3d electron at Ni^1+^ aligned antiparallel to the majority spin on the adjacent Fe site (Figure 6). The geometry of **NiFe**_**4**_**S**_**4**_ was optimized with three BS topologies that differ in the spin coupling within the Fe_4_S_4_ subcluster, resulting in structures with energies within 4.1 kcal/mol. The lowest-energy structure has a reasonable geometry with M–M and M–L distances having mean absolute errors (MAEs) of 0.056 and 0.028 Å, respectively, and was used for further BS calculations. An excited state in which the Ni^1+^ spin is parallel to the spin at the adjacent iron site was found to be 2.8 kcal/mol (970 cm^–1^) higher in energy, indicating spin coupling between Ni^1+^ and the Fe_4_S_4_ subcluster at the BS DFT level. Though significant, this interaction is weaker than that between the two mixed-valent 2Fe subsystems, estimated at 31 kcal/mol (11 000 cm^–1^). See the SI for spin density plots and further discussion.

For **Ni**_**2**_**Fe**_**3**_**S**_**4**_, the ground-state BS wavefunction has antiferromagnetic coupling between the high-spin central Fe^3+^ and the peripheral Ni^1+^ and high-spin Fe^2+^ (Figure 6). The optimized geometry has M–M and M–L MAEs of only 0.004 and 0.010 Å, respectively. An all-parallel-spin, *M*_S_ = 15/2 wavefunction was found 49 kcal/mol above the ground state at the same geometry, indicating large spin coupling, and significant σ interactions were evident in the localized orbitals between the central Fe and the other metals (see Figure S40). We recently reported a BS DFT investigation of such M–M bonding interactions in other clusters.^22^

Mössbauer isomer shifts were calculated for clusters **NiFe**_**4**_**S**_**4**_ and **Ni**_**2**_**Fe**_**3**_**S**_**4**_ using established procedures (see SI for further discussion). For **NiFe**_**4**_**S**_**4**_, the calculated isomer shift is 0.44 mm s^–1^ and is within uncertainty limits of the experimental value of 0.46 mm s^–1^. For **Ni**_**2**_**Fe**_**3**_**S**_**4**_, on the other hand, the calculations are accurate for the central Fe (0.36 vs 0.34 mm s^–1^ experimental) but are less accurate for the outside Fe sites (0.39 vs e 0.52 mm s^–1^ experimental). This deviation may arise from the deconvolution in the experimental spectra or from inaccuracy in the computations because of some unmodeled delocalization of 3d orbitals of the outside Fe onto the central Fe. With this caveat, the qualitative electronic structure (i.e., the local oxidation states and spin coupling topology) from the calculations fits well with the data for **Ni**_**2**_**Fe**_**3**_**S**_**4**_.

## Discussion

Fe_4_S_4_ cubanes are generally the most stable of iron–sulfur clusters, but we show here that the redox mismatch strategy enables the insertion of heterometals. Through a combination of structural and spectroscopic studies, we first coordinated nickel to the edge of a [Fe_4_S_4_] cluster, concomitant with electron transfer, to yield **NiFe**_**4**_**S**_**4**_. Some of the cuboidal Fe–S bond lengths are elongated in **NiFe**_**4**_**S**_**4**_ with respect to the starting cluster, implying that Fe–S bonds in the new cluster are weaker and poised to break. Switching to the more reactive IPrNi(η^6^-C_7_H_8_) precursor opens the cluster to give an unprecedented iron–sulfur cluster shape in **Ni**_**2**_**Fe**_**3**_**S**_**4**_ that has two perpendicular three-metal chains coupled physically and electronically through the tetrahedral central Fe. Each peripheral metal site is three coordinate, making **Ni**_**2**_**Fe**_**3**_**S**_**4**_ the only synthetic iron–sulfur cluster to feature more than one three-coordinate metal site.^57−59^ The formation of this cluster is like the reverse of the conversions from a Fe_3_S_4_ linear chain to MFe_3_S_4_ (M = Fe, Ni, Co) cubane clusters, which are known in both synthetic and biological systems.^60^ The reaction to form **Ni**_**2**_**Fe**_**3**_**S**_**4**_ is the first synthetic example of transforming a cubane cluster to one with a linear chain topology. The redox-mediated insertion of Ni into an Fe_4_S_4_ cluster is reminiscent of the biosynthesis of the *C* cluster, where nickel incorporation requires the *D* cluster (which plays a role in mediating electron transfer in CODH), suggesting that redox events are intimately linked to Ni insertion into the iron–sulfur cluster.^8,61^

**Figure 7:**
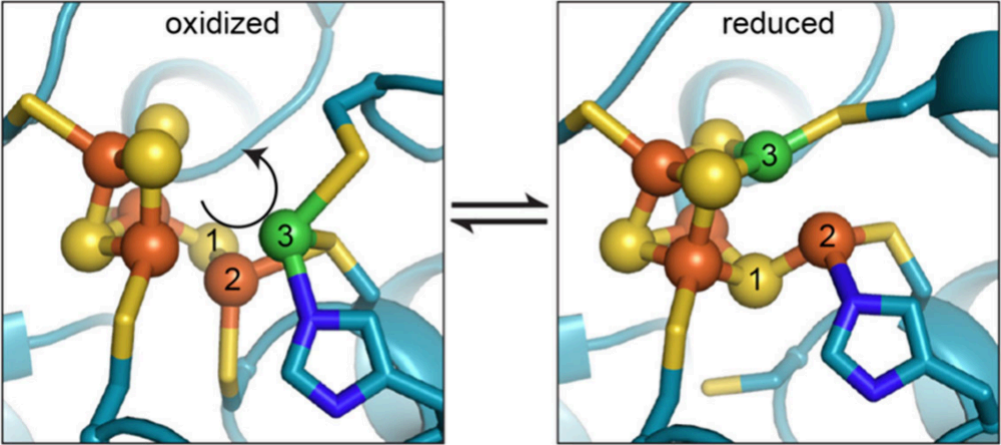
Redox transformation of the *C* cluster, as demonstrated by X-ray crystallographic studies on *D. vulgaris* CO dehydrogenase. Reprinted with permission from ref (23). Copyright 2018 eLife; used under a CC-BY license.

The redox-driven cluster rearrangement described here is also relevant to crystallographic work by Drennan on a reversible transformation of the *C* cluster that is illustrated in Figure 7.^23^ Oxidation of the sample with air gave a structure in which nickel lies outside the cluster, whereas reduction with dithionite caused nickel insertion to generate the topology of the active *C* cluster with the nickel embedded in the cluster. Though the oxidation levels are not specified by these crystallographic studies, it is noteworthy that reduction drives nickel insertion into the cluster. Similarly, Dobbek recently showed the *C* cluster can undergo reductive reactivation after O_2_ damage.^62^ In this scenario, the reductive activation eliminates a μ^2^-sulfide bridging the Ni and Fe_u_ sites. The synthetic studies described here verify the feasibility of reductive nickel insertion into iron–sulfur clusters with full characterization of the metal oxidation states. We also characterize the spin coupling within the clusters, which was not possible in the biological clusters.

In recent work, we utilized a preorganized WFe_2_ cluster to support a three-coordinate nickel site in a heterometallic cluster for the first time (Figure 1c).^22^ However, strong W–Fe and W–Ni bonding dominated the cluster’s valence electronic structure. The new compounds described here have no heavier metal to complicate the comparison to biological clusters. The computations indicate significant antiferromagnetic coupling but overall weaker σ interactions among the 3d metals, as 3d orbitals are more contracted than the 5d orbitals from tungsten in the earlier reported compounds. There are similarities, though, in that the nickel sites are best described as Ni^1+^. The metal oxidation state assignments for both **NiFe**_**4**_**S**_**4**_ and **Ni**_**2**_**Fe**_**3**_**S**_**4**_ are established using magnetic and spectroscopic probes as well as broken-symmetry DFT. In both clusters, the experimental and calculated Ni pre-edge transitions clearly support a Ni^1+^ assignment. This indicates that Ni^1+^ is a reasonable oxidation state in biologically relevant sulfide-supported coordination environments. In **NiFe**_**4**_**S**_**4**_, the Ni^1+^ is exchange coupled to a mixed-valence [Fe_4_S_4_]^2+^ cluster, supporting the plausibility of this oxidation state topology as assigned to the Fe–S cluster and Ni_p_ site in preparations of the A site of acetyl coenzyme A.^63,64^

The oxidation state of nickel is unclear in the reduced forms of the CODH *C* cluster, but one proposal is that the two-electron reduction from *C*_red1_ to *C*_red2_ is nickel localized; if the iron sites remain in the same oxidation state, this would imply that a nickel(II) site is reduced to nickel(0).^65,66,5,67^ Such a low oxidation state of nickel is unprecedented in biological systems and in synthetic systems usually requires π-acidic ligand environments to stabilize the low oxidation states, which are very dissimilar to the π-donating S-based ligands in the *C* cluster. The redox transformations described here indicate that reduction of the *C*_red1_ cluster would not give nickel(0); instead, the electron density would spread into the iron–sulfur part of the cluster. For example, addition of a nickel(0) source gives **NiFe**_**4**_**S**_**4**_, with nickel(I) and a reduced cluster. This redox shift is reversible as addition of isocyanides regenerates nickel(0), which is released from the cluster. Thus, the iron–sulfur component can store reducing equivalents, in a manner similar to clusters by Suess where CO binding causes shifts in the electron density to the CO-bound iron from the other three iron sites in the cluster.^68^ In the *C* cluster, this could be achieved either by storing reducing equivalents in the Fe_3_S_4_ cuboid or by forming a dative bonding interaction with the Fe_u_ site. We cannot rule out the idea that nickel(0) present in the CODH cluster is stabilized by some factor that was not modeled here (e.g., hydrogen bonding), but our studies suggest that the oxidation state of nickel in the *C* cluster is unlikely to be less than +1.

## Conclusions

Here, we describe redox-driven reactions that lead to new NiFeS clusters featuring low-coordinate metal sites. Addition of the Ni^0^ precursor IMesNi(1,5-hexadiene) to an oxidized [Fe_4_S_4_]^3+^ cluster results in coordination of the Ni and electron transfer to the Fe_4_S_4_ cubane. The solid-state structure of the resulting **NiFe**_**4**_**S**_**4**_ displays long Fe–S bonds in proximity to the coordinated nickel. Increasing the steric profile of the precursor in IPrNi(1,5-hexadiene) results in a species with comparable spectroscopic signatures to **NiFe**_**4**_**S**_**4**_; however, this species could not be isolated. Addition of 2 equiv of IPrNi(η^6^-C_7_H_8_) to the same [Fe_4_S_4_]^3+^ cluster generates an unprecedented **Ni**_**2**_**Fe**_**3**_**S**_**4**_ cluster. These reactions imply that electron transfer into the cluster weakens the Fe–S bonds in the Fe_4_S_4_ cluster and enables the insertion of nickel atoms into the cluster along with an iron atom being ejected. This illustrates how redox processes can drive conformational and compositional changes in heterometallic clusters, yielding products that have sites that are unusually low-coordinate. The ability of these unsaturated sites to bind CODH substrates will be an interesting topic of future study.^69^

In both **NiFe**_**4**_**S**_**4**_ and **Ni**_**2**_**Fe**_**3**_**S**_**4**_, the nickel centers are three coordinate and best described as Ni^1+^, as determined by magnetic, spectroscopic, and computational analysis. While previous work had identified that electrons can move into W–Ni bonds to avoid Ni^0^, the NiFeS clusters described here do not require strong metal–metal σ interactions. Rather, the electron donated to the cluster by Ni^0^ is stored on the iron sites. These clusters are the first experimental demonstration of the feasibility of accessing Ni^1+^ sites within metal–sulfur clusters having only iron and nickel. Our synthetic approach to reductively insert Ni into an iron–sulfur cluster bears similarity to the proposed biosynthesis of the *C* cluster, which uses reduction to incorporate nickel into iron–sulfur cubanes.^24^

## Data Availability

Crystallographic data are in the CCSD under deposition numbers 2356745 and 2356746. All other relevant data generated and analyzed during this study, which include experimental, spectroscopic, and computational data, are available in the Edmond Open Research Data Repository at 10.17617/3.FV4QYX and in the Supporting Information described below.

## Supporting Information Available

Experimental details, spectroscopic data, computational data, and additional discussion (PDF)
Crystallographic information (ZIP)

## References

[ref1] Parkin A.; Seravalli J.; Vincent K. A.; Ragsdale S. W.; Armstrong F. A. Rapid and Efficient Electrocatalytic CO_2_/CO Interconversions by *Carboxydothermus Hydrogenoformans* CO Dehydrogenase I on an Electrode. J. Am. Chem. Soc. 2007, 129 (34), 10328–10329. 10.1021/ja073643o. 17672466 PMC3056240

[ref2] Shin W.; Lee S. H.; Shin J. W.; Lee S. P.; Kim Y. Highly Selective Electrocatalytic Conversion of CO_2_ to CO at –0.57 V (NHE) by Carbon Monoxide Dehydrogenase from *Moorella Thermoacetica*. J. Am. Chem. Soc. 2003, 125 (48), 14688–14689. 10.1021/ja037370i. 14640627

[ref3] Svetlitchnyi V.; Peschel C.; Acker G.; Meyer O. Two Membrane-Associated NiFeS-Carbon Monoxide Dehydrogenases from the Anaerobic Carbon-Monoxide-Utilizing Eubacterium *Carboxydothermus Hydrogenoformans.*. J. Bacteriol. 2001, 183 (17), 5134–5144. 10.1128/JB.183.17.5134-5144.2001. 11489867 PMC95390

[ref4] Ribbe M. W. Insights into the Mechanism of Carbon Monoxide Dehydrogenase at Atomic Resolution. Angew. Chem., Int. Ed. 2015, 54 (29), 8337–8339. 10.1002/anie.201503979. 26031600

[ref5] Can M.; Armstrong F. A.; Ragsdale S. W. Structure, Function, and Mechanism of the Nickel Metalloenzymes, CO Dehydrogenase, and Acetyl-CoA Synthase. Chem. Rev. 2014, 114 (8), 4149–4174. 10.1021/cr400461p. 24521136 PMC4002135

[ref6] King G. M.; Weber C. F. Distribution, Diversity and Ecology of Aerobic CO-Oxidizing Bacteria. Nat. Rev. Microbiol. 2007, 5 (2), 107–118. 10.1038/nrmicro1595. 17224920

[ref7] Jeoung J.-H.; Dobbek H. Carbon Dioxide Activation at the Ni,Fe-Cluster of Anaerobic Carbon Monoxide Dehydrogenase. Science 2007, 318 (5855), 1461–1464. 10.1126/science.1148481. 18048691

[ref8] Wittenborn E. C.; Cohen S. E.; Merrouch M.; Léger C.; Fourmond V.; Dementin S.; Drennan C. L. Structural Insight into Metallocofactor Maturation in Carbon Monoxide Dehydrogenase. J. Biol. Chem. 2019, 294 (35), 13017–13026. 10.1074/jbc.RA119.009610. 31296570 PMC6721931

[ref9] DeRose V. J.; Telser J.; Anderson M. E.; Lindahl P. A.; Hoffman B. M. A Multinuclear ENDOR Study of the C-Cluster in CO Dehydrogenase from *Clostridium Thermoaceticum*: Evidence for H_x_O and Histidine Coordination to the [Fe_4_S_4_] Center. J. Am. Chem. Soc. 1998, 120 (34), 8767–8776. 10.1021/ja9731480.

[ref10] Beinert H.; Holm R. H.; Münck E. Iron-Sulfur Clusters: Nature’s Modular, Multipurpose Structures. Science 1997, 277 (5326), 653–659. 10.1126/science.277.5326.653. 9235882

[ref11] Holm R. H.; Lo W. Structural Conversions of Synthetic and Protein-Bound Iron–Sulfur Clusters. Chem. Rev. 2016, 116 (22), 13685–13713. 10.1021/acs.chemrev.6b00276. 27933770

[ref12] Ciurli S.; Yu S. B.; Holm R. H.; Srivastava K. K. P.; Munck E. Synthetic Nickel-Iron NiFe_3_Q_4_ Cubane-Type Clusters (S = 3/2) by Reductive Rearrangement of Linear [Fe_3_Q_4_(SEt)_4_]^3-^ (Q = Sulfur, Selenium). J. Am. Chem. Soc. 1990, 112 (22), 8169–8171. 10.1021/ja00178a053.

[ref13] Ciurli S.; Ross P. K.; Scott M. J.; Yu S. B.; Holm R. H. Synthetic Nickel-Containing Heterometal Cubane-Type Clusters with NiFe_3_Q_4_ Cores (Q = Sulfur, Selenium). J. Am. Chem. Soc. 1992, 114 (13), 5415–5423. 10.1021/ja00039a063.

[ref14] Zhou J.; Raebiger J. W.; Crawford C. A.; Holm R. H. Metal Ion Incorporation Reactions of the Cluster [Fe_3_S_4_(LS_3_)]^3-^, Containing the Cuboidal [Fe_3_S_4_]^0^ Core. J. Am. Chem. Soc. 1997, 119 (27), 6242–6250. 10.1021/ja9704186.

[ref15] Panda R.; Zhang Y.; McLauchlan C. C.; Venkateswara Rao P.; Tiago de Oliveira F. A.; Münck E.; Holm R. H. Initial Structure Modification of Tetrahedral to Planar Nickel(II) in a Nickel–Iron–Sulfur Cluster Related to the C-Cluster of Carbon Monoxide Dehydrogenase. J. Am. Chem. Soc. 2004, 126 (20), 6448–6459. 10.1021/ja030627s. 15149242

[ref16] Panda R.; Berlinguette C. P.; Zhang Y.; Holm R. H. Synthesis of MFe_3_S_4_ Clusters Containing a Planar M^II^ Site (M = Ni, Pd, Pt), a Structural Element in the C-Cluster of Carbon Monoxide Dehydrogenase. J. Am. Chem. Soc. 2005, 127 (31), 11092–11101. 10.1021/ja052381s. 16076217 PMC2631389

[ref17] Coucouvanis D.; Salifoglou A.; Kanatzidis M. G.; Dunham W. R.; Simopoulos A.; Kostikas A. Synthesis, Structural Characterization, and Electronic Properties of the [(Fe_6_S_6_X_6_)(M(CO)_3_)_2_]^n-^ Anions (M = Mo, W; n = 3, 4; X = Cl, Br, I). Heteronuclear Clusters of Possible Structural Relevance to the Iron-Molybdenum-Sulfur Center in Nitrogenase. Inorg. Chem. 1988, 27 (22), 4066–4077. 10.1021/ic00295a034.

[ref18] Al-Ahmad S. A.; Salifoglou A.; Kanatzidis M. G.; Dunham W. R.; Coucouvanis D. Octanuclear Heterometallic Clusters with Rhombic Dodecahedral Cores. The Synthesis, Structural Characterization, and Properties of the [Fe_6_S_6_(p-RPhO)_6_[M(CO_3_]_2_]^n-^ Clusters (M = Mo, n = 3, R = Me, OMe, N(Me_2_); M = W, n = 3, R = Me; M = Mo, n = 4, R = Me, OMe, COMe). Precursors for Synthetic Analogs for the Fe/Mo/S Site in Nitrogenase. Inorg. Chem. 1990, 29 (5), 927–938. 10.1021/ic00330a007.

[ref19] Junghans C.; Saak W.; Pohl S. Hexacapped Mixed-Metal Cubic [M_8_S_6_] Clusters. Formation and Structures of [Fe_6_Ni_2_S_6_I_6_(PMePh_2_)_2_]^2–^ and [Fe_4_Ni_4_S_6_I_4_(PMePh_2_)_4_]. J. Chem. Soc. Chem. Commun. 1994, 2327–2328. 10.1039/c39940002327.

[ref20] Zheng B.; Chen X.-D.; Zheng S.-L.; Holm R. H. Selenium as a Structural Surrogate of Sulfur: Template-Assisted Assembly of Five Types of Tungsten–Iron–Sulfur/Selenium Clusters and the Structural Fate of Chalcogenide Reactants. J. Am. Chem. Soc. 2012, 134 (14), 6479–6490. 10.1021/ja3010539. 22424175 PMC3353770

[ref21] Xu G.; Wang Z.; Ling R.; Zhou J.; Chen X.-D.; Holm R. H. Ligand Metathesis as Rational Strategy for the Synthesis of Cubane-Type Heteroleptic Iron–Sulfur Clusters Relevant to the FeMo Cofactor. Proc. Natl. Acad. Sci. U. S. A. 2018, 115 (20), 5089–5092. 10.1073/pnas.1801025115. 29654147 PMC5960317

[ref22] Wilson D. W. N.; Fataftah M. S.; Mathe Z.; Mercado B. Q.; DeBeer S.; Holland P. L. Three-Coordinate Nickel and Metal–Metal Interactions in a Heterometallic Iron–Sulfur Cluster. J. Am. Chem. Soc. 2024, 146 (6), 4013–4025. 10.1021/jacs.3c12157. 38308743 PMC10993082

[ref23] Wittenborn E. C.; Merrouch M.; Ueda C.; Fradale L.; Léger C.; Fourmond V.; Pandelia M.-E.; Dementin S.; Drennan C. L. Redox-Dependent Rearrangements of the NiFeS Cluster of Carbon Monoxide Dehydrogenase. eLife 2018, 7, e39451 10.7554/eLife.39451. 30277213 PMC6168284

[ref24] Alfano M.; Cavazza C. Structure, Function, and Biosynthesis of Nickel-Dependent Enzymes. Protein Sci. 2020, 29 (5), 1071–1089. 10.1002/pro.3836. 32022353 PMC7184782

[ref25] Amara P.; Mouesca J.-M.; Volbeda A.; Fontecilla-Camps J. C. Carbon Monoxide Dehydrogenase Reaction Mechanism: A Likely Case of Abnormal CO_2_ Insertion to a Ni–H Bond. Inorg. Chem. 2011, 50 (5), 1868–1878. 10.1021/ic102304m. 21247090

[ref26] Wu J.; Faller J. W.; Hazari N.; Schmeier T. J. Stoichiometric and Catalytic Reactions of Thermally Stable Nickel(0) NHC Complexes. Organometallics 2012, 31 (3), 806–809. 10.1021/om300045t.

[ref27] Hoshimoto Y.; Hayashi Y.; Suzuki H.; Ohashi M.; Ogoshi S. One-Pot, Single-Step, and Gram-Scale Synthesis of Mononuclear [(η^6^-Arene)Ni(*N*-Heterocyclic Carbene)] Complexes: Useful Precursors of the Ni^0^–NHC Unit. Organometallics 2014, 33 (5), 1276–1282. 10.1021/om500088p.

[ref28] Ohki Y.; Sunada Y.; Tatsumi K. Synthesis of [2Fe–2S] and [4Fe–4S] Clusters Having Terminal Amide Ligands from an Iron(II) Amide Complex. Chem. Lett. 2005, 34 (2), 172–173. 10.1246/cl.2005.172.

[ref29] Sharp C. R.; Duncan J. S.; Lee S. C. [Fe_4_S_4_]^q^ Cubane Clusters (q = 4+, 3+, 2+) with Terminal Amide Ligands. Inorg. Chem. 2010, 49 (14), 6697–6705. 10.1021/ic100742c. 20553035

[ref30] Strop P.; Takahara P. M.; Chiu H.-J.; Angove H. C.; Burgess B. K.; Rees D. C. Crystal Structure of the All-Ferrous [4Fe-4S]^0^ Form of the Nitrogenase Iron Protein from *Azotobacter Vinelandii*. Biochemistry 2001, 40 (3), 651–656. 10.1021/bi0016467. 11170381

[ref31] Grunwald L.; Clémancey M.; Klose D.; Dubois L.; Gambarelli S.; Jeschke G.; Wörle M.; Blondin G.; Mougel V. A Complete Biomimetic Iron-Sulfur Cubane Redox Series. Proc. Natl. Acad. Sci. U. S. A. 2022, 119 (31), e2122677119 10.1073/pnas.2122677119. 35881795 PMC9351461

[ref32] Olechnowicz F.; Hillhouse G. L.; Jordan R. F. Synthesis and Reactivity of NHC-Supported Ni_2_(μ^2^-η_2_,η^2^-S_2_)-Bridging Disulfide and Ni_2_(μ-S)_2_-Bridging Sulfide Complexes. Inorg. Chem. 2015, 54 (6), 2705–2712. 10.1021/ic502892r. 25742125

[ref33] Grunwald L.; Inoue M.; Carril P. C.; Wörle M.; Mougel V. Gated Electron Transfers at Synthetic Iron-Sulfur Cubanes. Chem. 2024, 10, 365–387. 10.1016/j.chempr.2023.09.023.

[ref34] Deng L.; Holm R. H. Stabilization of Fully Reduced Iron–Sulfur Clusters by Carbene Ligation: The [Fe_n_S_n_]^0^ Oxidation Levels (n = 4, 8). J. Am. Chem. Soc. 2008, 130 (30), 9878–9886. 10.1021/ja802111w. 18593124 PMC2527442

[ref35] Brown A. C.; Suess D. L. M. Controlling Substrate Binding to Fe_4_S_4_ Clusters through Remote Steric Effects. Inorg. Chem. 2019, 58 (8), 5273–5280. 10.1021/acs.inorgchem.9b00360. 30901206

[ref36] Hsieh C.-H.; Darensbourg M. Y. A {Fe(NO)_3_}^10^ Trinitrosyliron Complex Stabilized by an N-Heterocyclic Carbene and the Cationic and Neutral {Fe(NO)_2_}^9/10^ Products of Its NO Release. J. Am. Chem. Soc. 2010, 132 (40), 14118–14125. 10.1021/ja104135x. 20857969

[ref37] Zhang S.; Warren T. H. Three Coordinate Models for the Binuclear Cu_A_ Electron-Transfer Site. Chem. Sci. 2013, 4 (4), 1786–1792. 10.1039/c3sc21936d.

[ref38] Kupper C.; Rees J. A.; Dechert S.; DeBeer S.; Meyer F. Complete Series of {FeNO}^8^, {FeNO}^7^, and {FeNO}^6^ Complexes Stabilized by a Tetracarbene Macrocycle. J. Am. Chem. Soc. 2016, 138 (25), 7888–7898. 10.1021/jacs.6b00584. 27191681

[ref39] Liu Y.; Chatterjee S.; Cutsail G. E. I.; Peredkov S.; Gupta S. K.; Dechert S.; DeBeer S.; Meyer F. Cu_4_S Cluster in “0-Hole” and “1-Hole” States: Geometric and Electronic Structure Variations for the Active Cu_Z_* Site of N_2_O Reductase. J. Am. Chem. Soc. 2023, 145 (33), 18477–18486. 10.1021/jacs.3c04893. 37565682 PMC10450684

[ref40] Wilson D. W. N.; Thompson B. C.; Collauto A.; Hooper R. X.; Knapp C. E.; Roessler M. M.; Musgrave R. A. Mixed Valence {Ni^2+^Ni^1+^} Clusters as Models of Acetyl Coenzyme A Synthase Intermediates. J. Am. Chem. Soc. 2024, 146 (30), 21034–21043. 10.1021/jacs.4c06241. 39023163 PMC11295191

[ref41] Girerd J. J.; Papaefthymiou G. C.; Watson A. D.; Gamp E.; Hagen K. S.; Edelstein N.; Frankel R. B.; Holm R. H. Electronic Properties of the Linear Antiferromagnetically Coupled Clusters [Fe_3_S_4_(SR)_4_]^3-^, Structural Isomers of the Iron-Sulfur(1+) [Fe_3_S_4_]^1+^ Unit in Iron-Sulfur Proteins. J. Am. Chem. Soc. 1984, 106 (20), 5941–5947. 10.1021/ja00332a031.

[ref42] Kennedy M. C.; Kent T. A.; Emptage M.; Merkle H.; Beinert H.; Münck E. Evidence for the Formation of a Linear [3Fe-4S] Cluster in Partially Unfolded Aconitase. J. Biol. Chem. 1984, 259 (23), 14463–14471. 10.1016/S0021-9258(17)42622-6. 6094558

[ref43] Stoll S.; Schweiger A. EasySpin, a Comprehensive Software Package for Spectral Simulation and Analysis in EPR. J. Magn. Reson. 2006, 178 (1), 42–55. 10.1016/j.jmr.2005.08.013. 16188474

[ref44] Venkateswara Rao P.; Holm R. H. Synthetic Analogues of the Active Sites of Iron–Sulfur Proteins. Chem. Rev. 2004, 104 (2), 527–560. 10.1021/cr020615+. 14871134

[ref45] Page M. J.; Lu W. Y.; Poulten R. C.; Carter E.; Algarra A. G.; Kariuki B. M.; Macgregor S. A.; Mahon M. F.; Cavell K. J.; Murphy D. M.; Whittlesey M. K. Three-Coordinate Nickel(I) Complexes Stabilised by Six-, Seven- and Eight-Membered Ring N-Heterocyclic Carbenes: Synthesis, EPR/DFT Studies and Catalytic Activity. Chem. Eur. J. 2013, 19 (6), 2158–2167. 10.1002/chem.201202950. 23292787

[ref46] Lin C.-Y.; Power P. P. Complexes of Ni(I): A “Rare” Oxidation State of Growing Importance. Chem. Soc. Rev. 2017, 46 (17), 5347–5399. 10.1039/C7CS00216E. 28675200

[ref47] Finnegan M. G.; Conover R. C.; Park J.-B.; Zhou Z. H.; Adams M. W. W.; Johnson M. K. Electronic, Magnetic, Redox, and Ligand-Binding Properties of [MFe_3_S_4_] Clusters (M = Zn, Co, Mn) in *Pyrococcus Furiosus* Ferredoxin. Inorg. Chem. 1995, 34 (21), 5358–5369. 10.1021/ic00125a040.

[ref48] Nagelski A. L.; Ozerov M.; Fataftah M. S.; Krzystek J.; Greer S. M.; Holland P. L.; Telser J. Electronic Structure of Three-Coordinate Fe^II^ and Co^II^ β-Diketiminate Complexes. Inorg. Chem. 2024, 63 (10), 4511–4526. 10.1021/acs.inorgchem.3c03388. 38408452 PMC11751772

[ref49] Bencini A.; Gatteschi D. EPR of Exchange Coupled Systems; Dover Books on Chemistry Series; Dover Publications, Inc., 2012.

[ref50] Musgrave K. B.; Laplaza C. E.; Holm R. H.; Hedman B.; Hodgson K. O. Structural Characterization of Metallopeptides Designed as Scaffolds for the Stabilization of Nickel(II)-Fe_4_S_4_ Bridged Assemblies by X-Ray Absorption Spectroscopy. J. Am. Chem. Soc. 2002, 124 (12), 3083–3092. 10.1021/ja011861q. 11902899

[ref51] Chandrasekaran P.; Chiang K. P.; Nordlund D.; Bergmann U.; Holland P. L.; DeBeer S. Sensitivity of X-Ray Core Spectroscopy to Changes in Metal Ligation: A Systematic Study of Low-Coordinate, High-Spin Ferrous Complexes. Inorg. Chem. 2013, 52 (11), 6286–6298. 10.1021/ic3021723. 23662855 PMC4029952

[ref52] Neese F. Software Update: The ORCA Program System—Version 5.0. WIREs Comput. Mol. Sci. 2022, 12, e1606 10.1002/wcms.1606.

[ref53] Colpas G. J.; Maroney M. J.; Bagyinka C.; Kumar M.; Willis W. S.; Suib S. L.; Mascharak P. K.; Baidya N. X-Ray Spectroscopic Studies of Nickel Complexes, with Application to the Structure of Nickel Sites in Hydrogenases. Inorg. Chem. 1991, 30 (5), 920–928. 10.1021/ic00005a010.

[ref54] Hugenbruch S.; Shafaat H. S.; Krämer T.; Delgado-Jaime M. U.; Weber K.; Neese F.; Lubitz W.; Debeer S. In Search of Metal Hydrides: An X-Ray Absorption and Emission Study of [NiFe] Hydrogenase Model Complexes. Phys. Chem. Chem. Phys. 2016, 18 (16), 10688–10699. 10.1039/C5CP07293J. 26924248

[ref55] Lewis L. C.; Sanabria-Gracia J. A.; Lee Y.; Jenkins A. J.; Shafaat H. S. Electronic Isomerism in a Heterometallic Nickel–Iron–Sulfur Cluster Models Substrate Binding and Cyanide Inhibition of Carbon Monoxide Dehydrogenase. Chem. Sci. 2024, 15, 5916 10.1039/D4SC00023D. 38665523 PMC11040638

[ref56] Desnoyer A. N.; He W.; Behyan S.; Chiu W.; Love J. A.; Kennepohl P. The Importance of Ligand-Induced Backdonation in the Stabilization of Square Planar d^10^ Nickel π-Complexes. Chem. Eur. J. 2019, 25 (20), 5259–5268. 10.1002/chem.201805987. 30693581

[ref57] Rodriguez M. M.; Stubbert B. D.; Scarborough C. C.; Brennessel W. W.; Bill E.; Holland P. L. Isolation and Characterization of Stable Iron(I) Sulfide Complexes. Angew. Chem., Int. Ed. 2012, 51 (33), 8247–8250. 10.1002/anie.201202211. PMC3970908 22821816

[ref58] DeRosha D. E.; Chilkuri V. G.; Van Stappen C.; Bill E.; Mercado B. Q.; DeBeer S.; Neese F.; Holland P. L. Planar Three-Coordinate Iron Sulfide in a Synthetic [4Fe-3S] Cluster with Biomimetic Reactivity. Nat. Chem. 2019, 11, 1019–1025. 10.1038/s41557-019-0341-7. 31611632 PMC6858550

[ref59] Brown A. C.; Suess D. L. M. An Iron–Sulfur Cluster with a Highly Pyramidalized Three-Coordinate Iron Center and a Negligible Affinity for Dinitrogen. J. Am. Chem. Soc. 2023, 145 (36), 20088–20096. 10.1021/jacs.3c07677. 37656961 PMC10824254

[ref60] Beinert H.; Holm R. H.; Münck E. Iron-Sulfur Clusters: Nature’s Modular, Multipurpose Structures. Science 1997, 277 (5326), 653–659. 10.1126/science.277.5326.653. 9235882

[ref61] Stripp S. T.; Duffus B. R.; Fourmond V.; Léger C.; Leimkühler S.; Hirota S.; Hu Y.; Jasniewski A.; Ogata H.; Ribbe M. W. Second and Outer Coordination Sphere Effects in Nitrogenase, Hydrogenase, Formate Dehydrogenase, and CO Dehydrogenase. Chem. Rev. 2022, 122 (14), 11900–11973. 10.1021/acs.chemrev.1c00914. 35849738 PMC9549741

[ref62] Basak Y.; Jeoung J.-H.; Domnik L.; Dobbek H. Stepwise O_2_-Induced Rearrangement and Disassembly of the [NiFe_4_(OH)(μ_3_-S)_4_] Active Site Cluster of CO Dehydrogenase. Angew. Chem., Int. Ed. 2023, 62 (32), e202305341 10.1002/anie.202305341. 37279092

[ref63] Tan X.; Martinho M.; Stubna A.; Lindahl P. A.; Münck E. Mössbauer Evidence for an Exchange-Coupled [Fe_4_S_4_]^1+^Ni_p_^1+^ A-Cluster in Isolated α Subunits of Acetyl-Coenzyme A Synthase/Carbon Monoxide Dehydrogenase. J. Am. Chem. Soc. 2008, 130 (21), 6712–6713. 10.1021/ja801981h. 18459773 PMC2701106

[ref64] Bender G.; Stich T. A.; Yan L.; Britt R. D.; Cramer S. P.; Ragsdale S. W. Infrared and EPR Spectroscopic Characterization of a Ni(I) Species Formed by Photolysis of a Catalytically Competent Ni(I)-CO Intermediate in the Acetyl-CoA Synthase Reaction. Biochemistry 2010, 49 (35), 7516–7523. 10.1021/bi1010128. 20669901 PMC2932805

[ref65] Hu Z.; Spangler N. J.; Anderson M. E.; Xia J.; Ludden P. W.; Lindahl P. A.; Münck E. Nature of the C-Cluster in Ni-Containing Carbon Monoxide Dehydrogenases. J. Am. Chem. Soc. 1996, 118 (4), 830–845. 10.1021/ja9528386.

[ref66] Anderson M. E.; Lindahl P. A. Spectroscopic States of the CO Oxidation/CO_2_ Reduction Active Site of Carbon Monoxide Dehydrogenase and Mechanistic Implications. Biochemistry 1996, 35 (25), 8371–8380. 10.1021/bi952902w. 8679595

[ref67] Lindahl P. A. Metal–Metal Bonds in Biology. J. Inorg. Biochem. 2012, 106 (1), 172–178. 10.1016/j.jinorgbio.2011.08.012. 22119810 PMC3232296

[ref68] Brown A. C.; Thompson N. B.; Suess D. L. M. Evidence for Low-Valent Electronic Configurations in Iron–Sulfur Clusters. J. Am. Chem. Soc. 2022, 144 (20), 9066–9073. 10.1021/jacs.2c01872. 35575703

[ref69] Newman-Stonebraker S. H.; Gerard T. J.; Holland P. L. Opportunities for Insight into the Mechanism of Efficient CO_2_/CO Interconversion at a Nickel-Iron Cluster in CO Dehydrogenase. Chem. 2024, 10 (6), 1655–1667. 10.1016/j.chempr.2024.04.012. 38966253 PMC11221784

