# Supplementary material for: Inserting Three-Coordinate Nickel into [4Fe-4S] Clusters
Source: ACS Cent Sci. 2024 Oct 3;10(10):1910–9. doi: 10.1021/acscentsci.4c00985 (PMC11503493; doi:10.1021/acscentsci.4c00985)
Supplement: Supplementary file 1 — oc4c00985_si_001.pdf [file oc4c00985_si_001.pdf]

Supplementary Information

Inserting Three-Coordinate Nickel into [4Fe-4S] clusters

Majed S. Fataftah,[†a] Daniel W. N. Wilson,[†a] Zachary Mathe,[†b] Theodore J. Gerard,[a] Brandon Q. Mercado,[a] Serena DeBeer,[*b] and Patrick L. Holland[*a]

[a]Department of Chemistry, Yale University, 225 Prospect St., New Haven, Connecticut 06520, USA

[b]Max Planck Institute for Chemical Energy Conversion, Stiftstrasse 34-36, 45470 Mülheim an der Ruhr, Germany

patrick.holland@yale.edu, serena.debeer@cec.mpg.de

General considerations 2

Synthetic procedures 3

Synthesis of [Na(15-crown-5)·Et_2O][IMesNiFe_4S_4($N(SiMe_3)_2$)$_4$], ($NiFe_4S_4$) 3

Synthesis of Na$(THF)_2$$(IPrNi)_2$$((Me_3Si)_2NFe)_2$$FeS_4$, ($Ni_2Fe_3S_4$) 4

XylNC addition to $NiFe_4S_4$ 5

^{1}H NMR spectra 6

FTIR spectra of metal complexes 12

UV/vis spectra of metal complexes 13

Mössbauer spectra and assignments 15

EPR spectra 19

Magnetic measurements 25

Cyclic voltammetry data 28

Crystallographic data 30

X-ray Absorption Spectroscopy 37

Calculations using Density Functional Theory 38

DFT Analysis of $NiFe_4S_4$ 38

DFT Analysis of $Ni_2Fe_3S_4$ 41

Computational X-ray Absorption Spectroscopy 42

Ni 4p_z Transitions 43

Mössbauer Isomer Shifts from DFT 45

References 47

General considerations

All manipulations were performed in a nitrogen-filled MBraun glovebox maintained below 1 ppm of O_2 and H_2O or using standard Schlenk techniques unless mentioned otherwise. Glassware was oven-dried at 150 °C for at least 12 h prior to use. Celite and molecular sieves were dried above 240 °C under vacuum for at least 12 h. Pentane, THF, hexanes, benzene, toluene, and diethyl ether were purified by passage through activated alumina and Q5 columns from Glass Contour Co, under argon. 2-Methyltetrahydrofuran (2-MeTHF) was freeze-pump-thawed (4 cycles) and stored over activated 4 Å molecular sieves for 3 days prior to use. All solvents were stored in gloveboxes over molecular sieves. Benzene-d_6 and THF-d_8 were vacuum transferred from a solution of potassium benzophenone ketyl and were stored over 4 Å molecular sieves. IPrNi(η^6-C_7H_8),[1] IPrNi(η^4-C_6H_{10}),[2] IMesNi(η^4-C_6H_{10}),[2] [Na(THF)$_2$][Fe_4S_4(N(SiMe$_3$)$_2$)$_2$],[3,4] and $FeCl_2$[5] were synthesized according to literature procedures. NaN(SiMe$_3$)$_2$ was purchased from Sigma Aldrich and recrystallized from Et_2O prior to use. NaSH was purchased from STREM Chemicals. NMR data were collected on either an Agilent 400 or 500 MHz spectrometer. Chemical shifts in ^1H NMR spectra are referenced to the residual protiated solvent peaks of C_6D_5H (7.16 ppm), THF-d_7 (δ 3.58 ppm) and C_7D_7H. Integrations often used deconvolution of broad peaks, using MestreNova. Elemental analyses were performed at the CENTC Elemental Analysis Facility at the University of Rochester. IR spectra were collected on an Alpha Platinum ATR IR Spectrometer. Notation used for IR assignment: s = strong, m = medium, w = weak, sh = shoulder. UV-vis spectra were recorded on a Cary 60 spectrometer using Schlenk-adapted quartz cuvettes with a 1 mm path length.

Synthetic procedures

Synthesis of [Na(15-crown-5)·Et_2O][IMesNiFe_4S_4(N($SiMe_3)_2)_4$], (**$NiFe_4S_4$**)

Na$(THF)_2Fe_4S_4(N(SiMe_3)_2)_4$ (160 mg, 0.14 mmol) was added with a stir bar to a 20 mL scintillation vial. Et_2O (12 mL) was added, and the mixture was cooled to –40 °C. Separately, IMesNi(η^4-C_6H_{10}) (62 mg, 0.14 mmol) was dissolved in cold Et_2O (–40 °C, 4 mL) and was then added dropwise to the reaction mixture. The mixture was stirred for 60 minutes while warming to room temperature. 15-crown-5 (33 mg, 0.15 mmol) was dissolved in Et_2O (2 mL) and added to the reaction mixture. The reaction mixture was stirred for another 30 minutes. Afterward, the volume of the reaction mixture was concentrated to 5 mL, filtered through Celite, then layered under 7 mL of hexanes. The vial was stored at –40 °C overnight to give crystals of **$NiFe_4S_4$** (160 mg, 69%). Typical isolated yields are in the range of 55–65%.

^{1}H NMR (400 MHz, benzene-*d_6*) δ (ppm) 8.16 (br, 4H, IMes *meta*-ArC**H**), 7.71 (br, 2H, IMes backbone C**H**=C**H**), 3.52 (br, THF), 3.26 (q, 3H, Et_2O C**H_2**), 3.08 (br, 6H, IMes *para*-C**H_3**), 2.90 (br, 20H, 15-crown-5), 2.67 (br, 18H, N(Si(C**H_3**$)_2)_3$), 2.18 (br, 12H, IMes *ortho*-C**H_3**), 1.3–1.7 (multiple overlapping broad resonances, 54H, N(Si(C**H_3**$)_2)_3$, 1.11 (t, Et_2O C**H_3**), 0.89 (multiplet, hexanes). The 1.3–1.7 ppm range has overlapping resonances corresponding to the three $N(Si(CH_3)_2)_3$, hexanes, and THF, which could not be resolved satisfactorily (see Fig S1).

IR (ATR, neat, cm^{-1}): 2954 (m), 2891 (m), 1615 (w), 1565 (w), 1481 (w), 1456 (w), 1399 (w), 1358 (w), 1308 (m), 1251 (s), 1239 (s), 1117 (s), 1097 (s), 1041 (w), 947 (s), 844 (s), 828 (s), 779 (s), 756 (s), 707 (s), 663 (s), 623 (m), 596 (w), 577 (w), 524 (w), 456 (w), 423 (w).

UV-vis (benzene, ε in mM^{-1} cm^{-1}): 365 nm (18), 675 nm (2.3).

Elemental analysis calculated for $C_{65}H_{127}Fe_4NiS_4N_6O_6Si_8Na$ (FW 1674.86): C, 43.35; H, 7.86; N, 4.89. Found: C, 43.87; H, 7.83; N, 4.22.

Mössbauer (80 K, solid): δ = 0.46 mm s^{-1}, $|\Delta E_Q|$ = 1.03 mm s^{-1}, Γ = 0.56 mm s^{-1}.

Synthesis of $Na(THF)_2(IPrNi)_2((Me_3Si)_2NFe)_2FeS_4$, ($Ni_2Fe_3S_4$)

$Na(THF)_2Fe_4S_4(N(SiMe_3)_2)_4$ (68 mg, 0.06 mmol) was dissolved in THF (4 mL) in a 20 mL scintillation vial with a stir bar added. Separately, IPrNi(η^6-C_7H_8) (68 mg, 0.12 mmol) was dissolved in toluene (4 mL) and was then added dropwise to the reaction mixture. The mixture was stirred for 60 minutes at ambient temperature, and then dried under vacuum. The dark residue was extracted with Et_2O (12 mL) and then filtered through a small pad of Celite. The eluent volume was concentrated under vacuum to 4 mL. HMDSO (4 mL) and THF (2-3 drops) were added to the Et_2O solution. The solution was concentrated under vacuum to a final volume of 4 mL and stored at –40 °C overnight to give the first crop of crystals of **$Ni_2Fe_3S_4$** (45 mg, 45%). The solution can be concentrated to 2-3 mL to give a second crop of crystals (total of 62 mg, 62%). Typical isolated yields are in the range of 35–60%.

Elemental analysis calcd for $C_{74}H_{126}Fe_3N_6Ni_2S_4Si_4O_2Na$ (FW 1680.4): C, 52.89; H, 7.56; N, 5.00. Found: C, 52.82; H, 7.36; N, 4.77.

^{1}H NMR (400 MHz, benzene–*d*$_6$) δ (ppm) 11.98 (4H), 4.49 (36H, N(Si(C**H**$_3$)$_2$)$_3$), 1.82 (8H, THF), 0.13 (8H, THF), –1.36 (4H), –2.14 (4H), –4.35 (4H), –4.95 (12H, CH(**CH$_3$**)$_2$), –6.17 (12H, CH(**CH$_3$**)$_2$), –8.45 (4H), –12.19 (4H), –13.97 (12H, CH(**CH$_3$**)$_2$), –19.5 (12H, CH(**CH$_3$**)$_2$). Specific assignment of the resonances with an integration of 4H has not been achieved since the IPr backbone protons, aryl para-CH, aryl meta-CH, and IPr $CH(CH_3)_2$ are each expected to integrate to 4 protons. However, the expected number of resonances are observed in the ^{1}H NMR spectrum.

^{1}H NMR (400 MHz, THF–*d*$_8$) δ (ppm) 10.6 (br, 8H, aryl *meta*-C**H** or C**H**(CH$_3$)$_2$), –4.2 (b, 24H, IPr CH(**CH$_3$**)$_2$), –6.3 (8H, aryl *meta*-C**H** or C**H**(CH$_3$)$_2$), –6.6 (24H, IPr CH(**CH$_3$**)$_2$), –8.49 (4H, aryl *para*-C**H** or C**H**=C**H**), –12.2 (4H, 4H, aryl *para*-C**H** or C**H**=C**H**), –14.8 (36H, N(Si(**CH$_3$**)$_3$)$_2$).

IR (ATR, neat, cm^{-1}): 3181 (w), 3148 (w), 3084 (w), 3039 (w), 2967 (m), 2956 (m), 2936 (m), 2897 (m), 2877 (m), 1662 (w), 1599 (w), 1565 (w), 1500 (w), 1465 (m), 1401 (m), 1368 (w), 1307 (s), 1272 (w), 1241 (s), 1210 (w), 1183 (w), 1120 (w), 1101 (m), 1054 (m), 990 (s), 970 (m), 943 (w), 867 (s), 847 (s), 828 (s), 801 (s), 756 (s), 732 (s), 713 (s), 697 (w), 670 (s), 629 (m), 614 (m), 553 (m), 456 (m), 415 (m).

UV-vis (benzene, ε in mM^{-1} cm^{-1}): 361 nm (24.0), 580 nm (5.0).

Mössbauer (80 K, solid): site 1 (67%): $\delta_1 = 0.49$ mm s^{-1}, $|\Delta E_Q|_1 = 0.75$ mm s^{-1}, $\Gamma_1 = 0.36$ mm s^{-1}; site 2 (33%): $\delta_2 = 0.34$ mm s^{-1}, $|\Delta E_Q|_2 = 0.83$ mm s^{-1}, $\Gamma_2 = 0.28$ mm s^{-1}.

XylNC addition to $NiFe_4S_4$

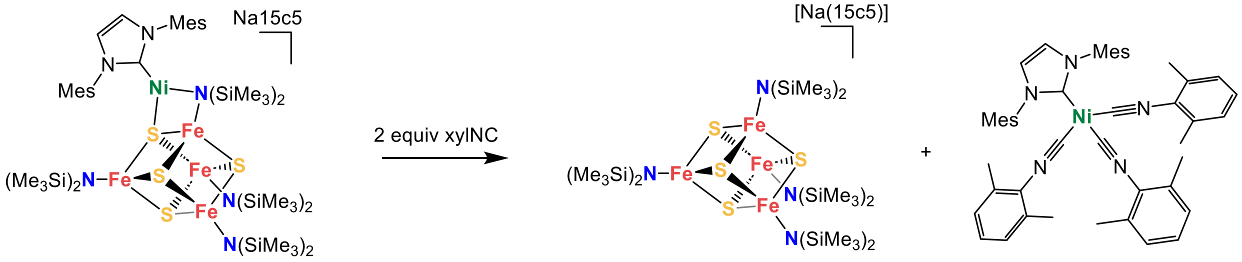

$NiFe_4S_4$ (23 mg, 0.014 mmol) was dissolved in Et_2O (6 mL) in a 20 mL scintillation vial with a stir bar added. Separately, xylyl isocyanide (4 mg, 0.03 mmol) was dissolved in Et_2O (1 mL) and was then added dropwise to the reaction mixture. The mixture was stirred for 5 minutes at ambient temperature. The reaction was subsequently layered under hexanes (7 mL) stored at –40 °C overnight to give crystalline material. The crystalline product was identified as $[Na(15c5)]_2Fe_4S_4(N(SiMe_3)_2)_4$ by single crystal X-ray diffraction and by ^1H NMR in C_6D_6 (see Fig S4). The eluent of the crystallization was dried under vacuum and inspected by ^1H NMR in C_6D_6 to reveal the second product of the reaction as $IMesNi(xylNC)_3$, the IPr analogue of which was previously reported (see Fig S5).[6] Addition of one equivalent of xylyl isocyanide leads to incomplete conversion to the above products.

^{1}H NMR of $[Na(15c5)]_2Fe_4S_4(N(SiMe_3)_2)_4$ **(400 MHz, C_6D_6)** δ (ppm) 3.2 (s, 36H, 15-crown-5), 1.56 (b, 72H, N(SiC**H$_3$**)$_2$).

^{1}H NMR of $IMesNi(xylNC)_3$ **(400 MHz, C_6D_6)** δ (ppm) 6.84 (s, 9H, xylylNC *ortho*-C**H**$_3$), 6.65 (s, 4H, IMes *meta*-C**H**), 6.45 (s, 2H, IMes backbone C**H**=C**H**), 2.3 (s, 13H, IMes *ortho*-C**H**$_3$), 2.25 (18H, xylylNC *ortho*-C**H**$_3$), 1.90 (6H, IMes *para*-C**H**$_3$).

^{1}H NMR spectra

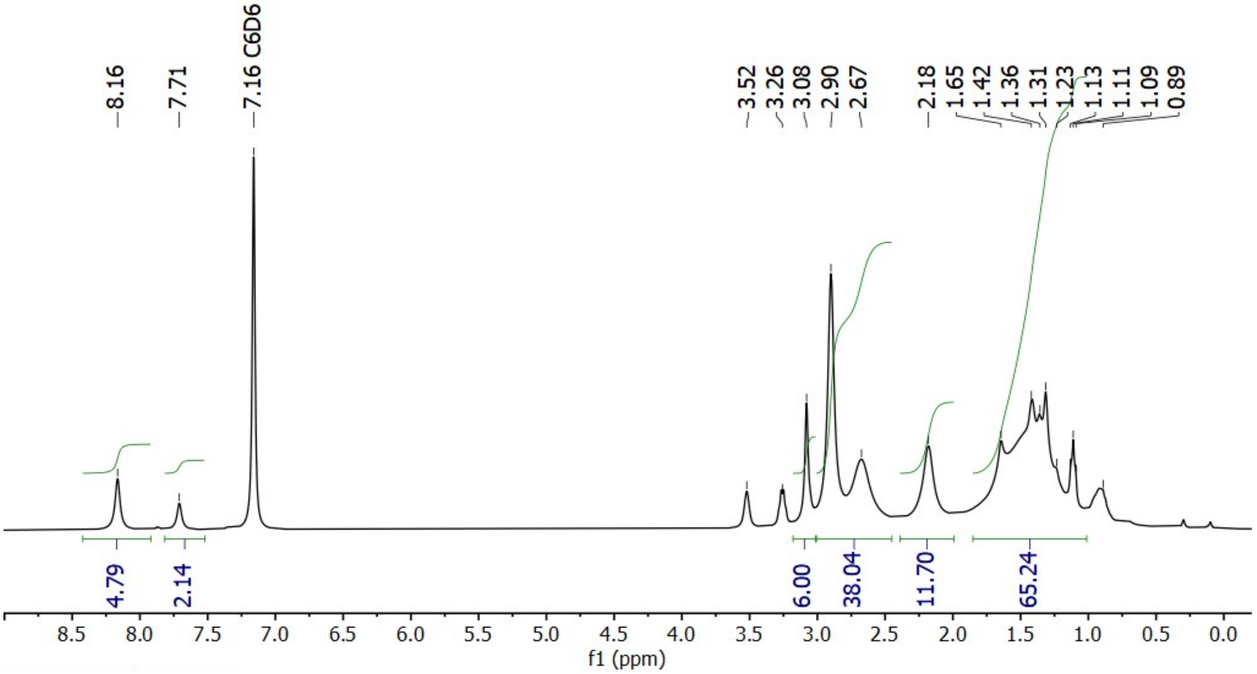

Figure S1. ^{1}H NMR (400 MHz) spectrum of **$NiFe_4S_4$** in C_6D_6.

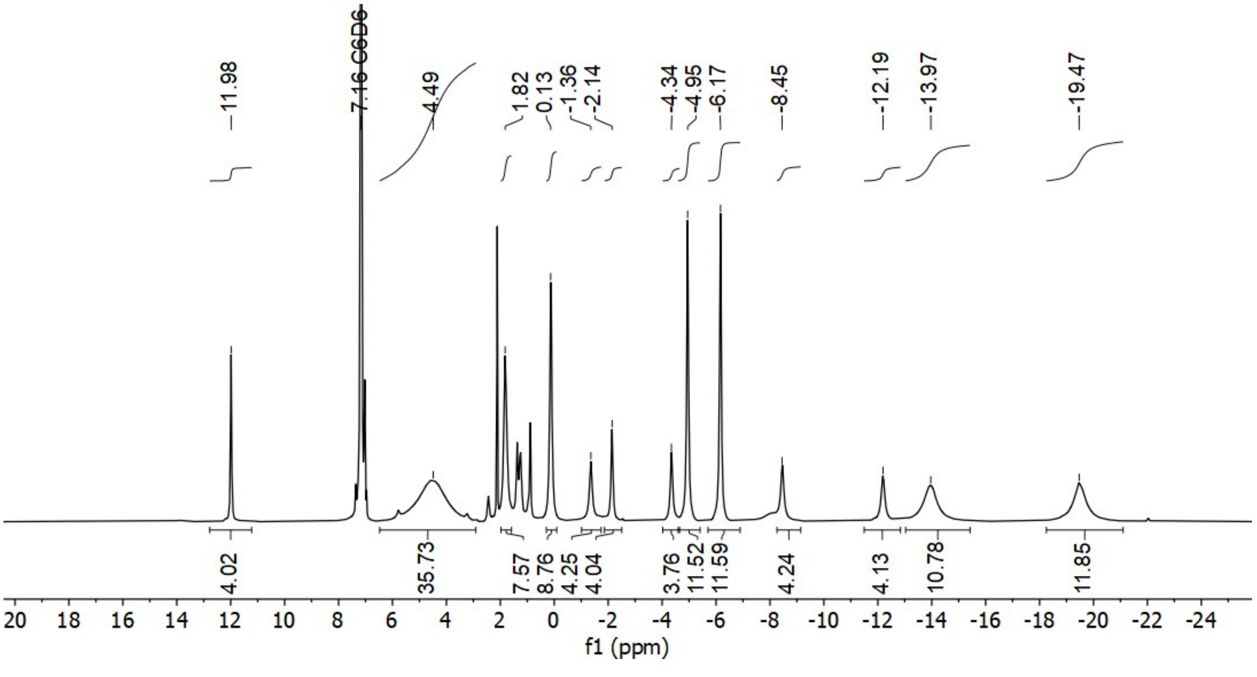

Figure S2. ^{1}H NMR (400 MHz) spectrum of **$Ni_2Fe_3S_4$** in C_6D_6.

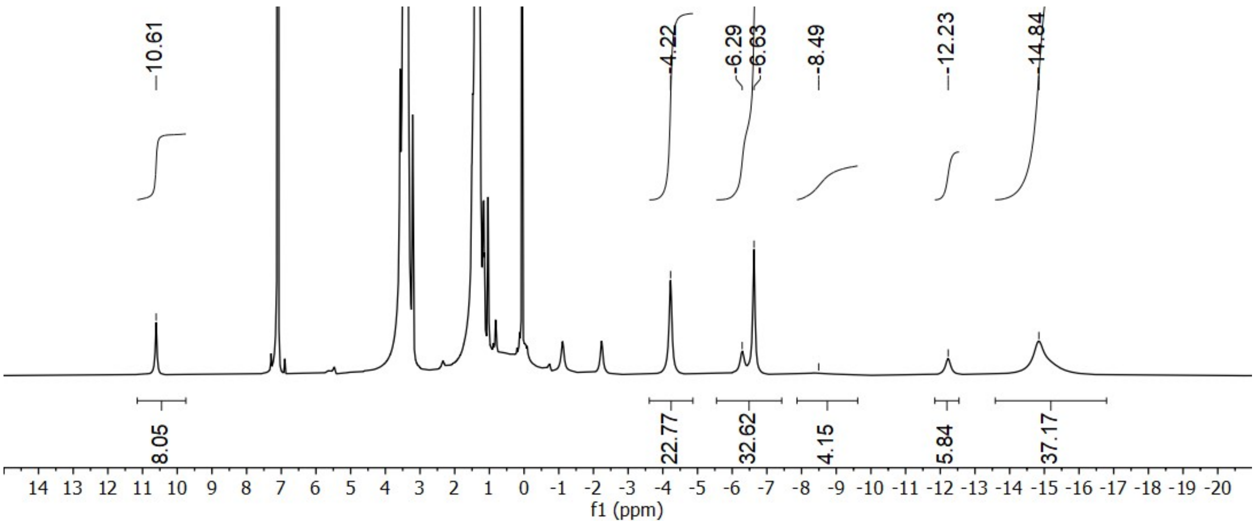

Figure S3. ^{1}H NMR (400 MHz) spectrum of $\mathbf{Ni_2Fe_3S_4}$ in C_6D_6 with the addition of 20 μL of THF. The higher symmetry ^{1}H NMR spectrum arises from sequestration of the inner sphere Na^+ cation, making the compound have D_{4h} symmetry in solution. The Na^+-bound THF molecules can be removed under vacuum. The solution phase symmetry of $\mathbf{Ni_2Fe_3S_4}$ is interchangeable between C_{4v} (without excess THF) and D_{4h} (with excess THF) by removal of the THF under vacuum.

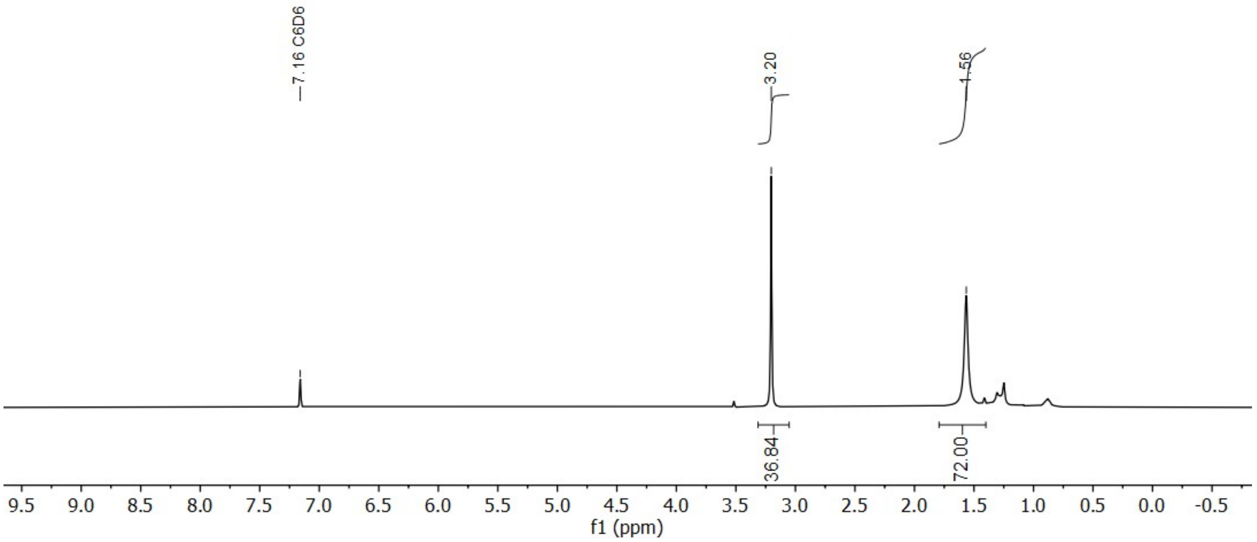

Figure S4. ^{1}H NMR of $[Na(15c5)]Fe_4S_4(N(SiMe_3)_2)_4$ in C_6D_6. ^{1}H NMR was taken of the crystalline material isolated from treating $\mathbf{NiFe_4S_4}$ with xylNC.

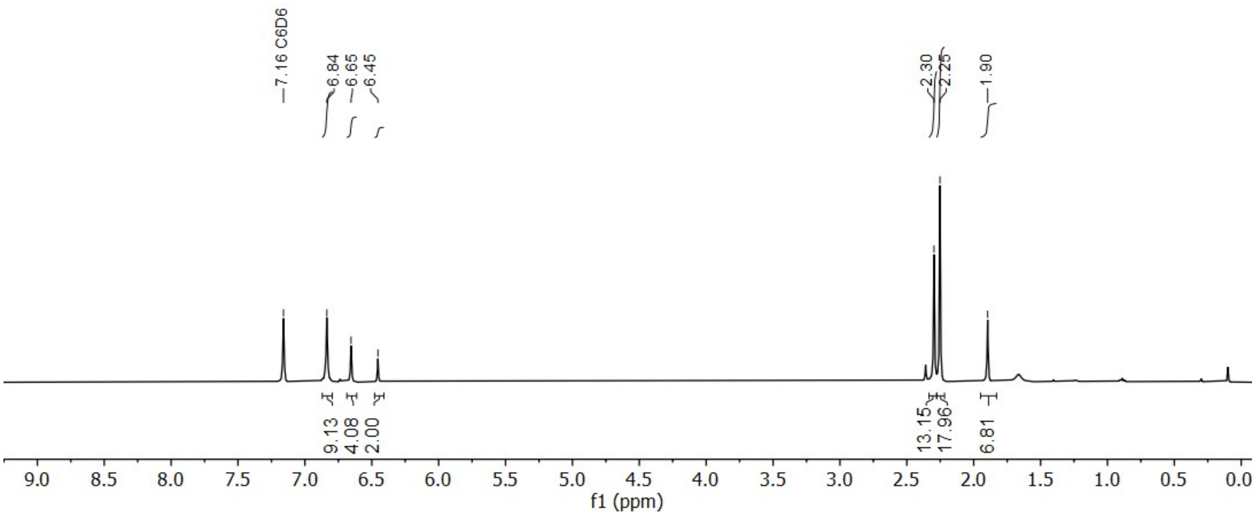

Figure S5. ^{1}H NMR of IMesNi(xylNC)$_3$ in C_6D_6. ^{1}H NMR was taken of the crystallization eluent from treating $\mathbf{NiFe_4S_4}$ with xylylNC.

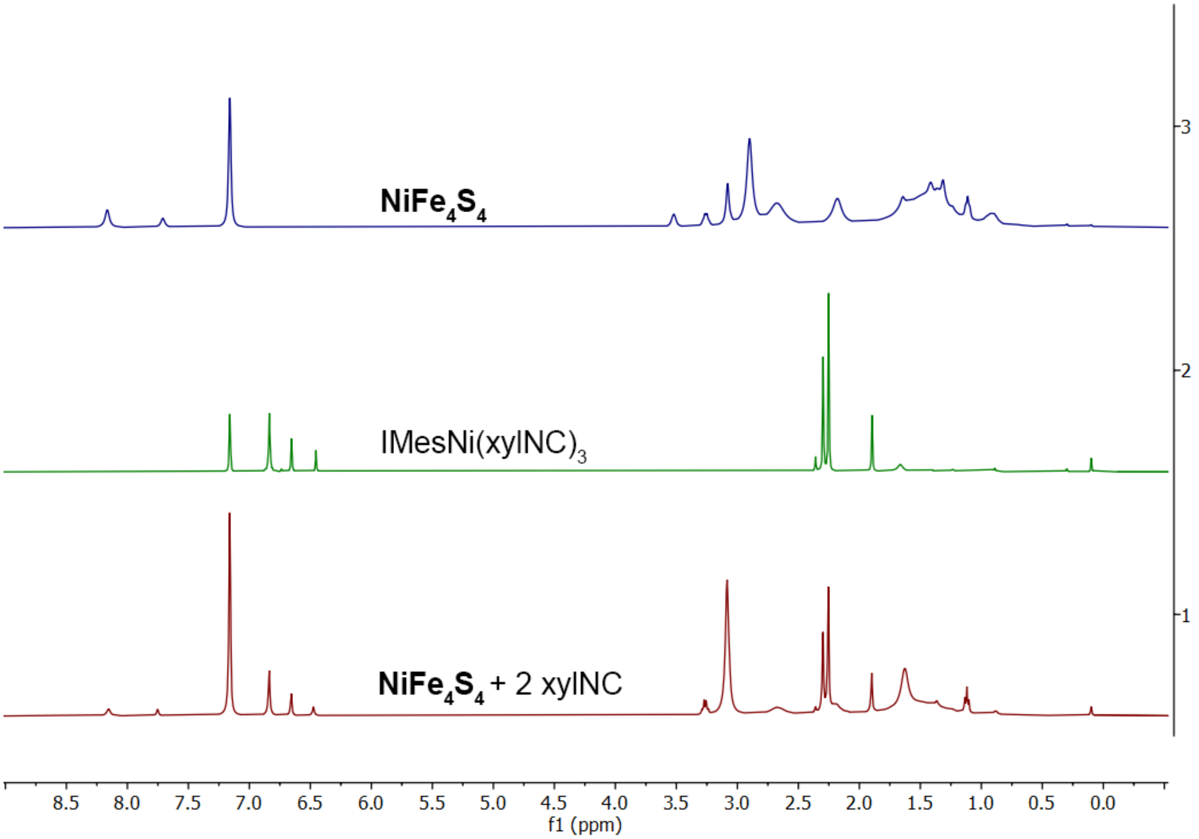

Figure S6. Overlay of the ^{1}H NMR spectra of $\mathbf{NiFe_4S_4}$ (top), independently prepared IMesNi(xylNC)$_3$ (middle), and the crude reaction between $\mathbf{NiFe_4S_4}$ and 2 equiv xylNC. The bottom spectrum shows a combination of the top and middle spectra.

Quantitative NMR Studies

In addition to the preparations above that led to isolation of **$Ni_2Fe_3S_4$**, we also measured crude yields (by comparing to a ^{1}H NMR integration standard) in order to understand the dependence of reaction outcome on the reaction conditions. In these experiments, $Na(THF)_2Fe_4S_4(N(SiMe_3)_2)_4$ (12.5 mg, 0.01 mmol) was dissolved in **solvent-1** (0.6 mL) in a 20 mL scintillation vial with a stir bar added. Separately, IPrNi(η^6-C_7H_8) (**x** equiv) was dissolved in C_7D_8 (0.4 mL). Both solutions were either at room temperature or cooled at –78 °C for 30 minutes, and the IPrNi(η^6-C_7H_8) was added dropwise to the reaction mixture. After stirring for time **t**, an aliquot was then added to an NMR tube containing a $NiCp_2$ capillary of known concentration, and a yield was determined using ^{1}H NMR integrations. If **solvent-1** was THF-d_8, the aliquot was dried under vacuum and then the ^{1}H NMR spectrum was measured as a solution in toluene-d_8.

These data indicate that THF plays an important role in maximizing the yield.

Table S1. Summary of yields of **$Ni_2Fe_3S_4$** as determined by ^{1}H NMR spectroscopy.

Trial	**x** (equiv)	**t** (min)	**solvent-1**	**temperature (°C)**	% Yield **$Ni_2Fe_3S_4$**
1	2.0	120	Toluene-d_8	–78	6
2	2.3	120	Toluene-d_8	–78	10
3	2.5	120	Toluene-d_8	–78	17
4	2.8	120	Toluene-d_8	–78	20
5	3.1	120	Toluene-d_8	–78	20
6	2.0	60	THF-d_8	–78	24
7	2.0	60	THF-d_8	22	45

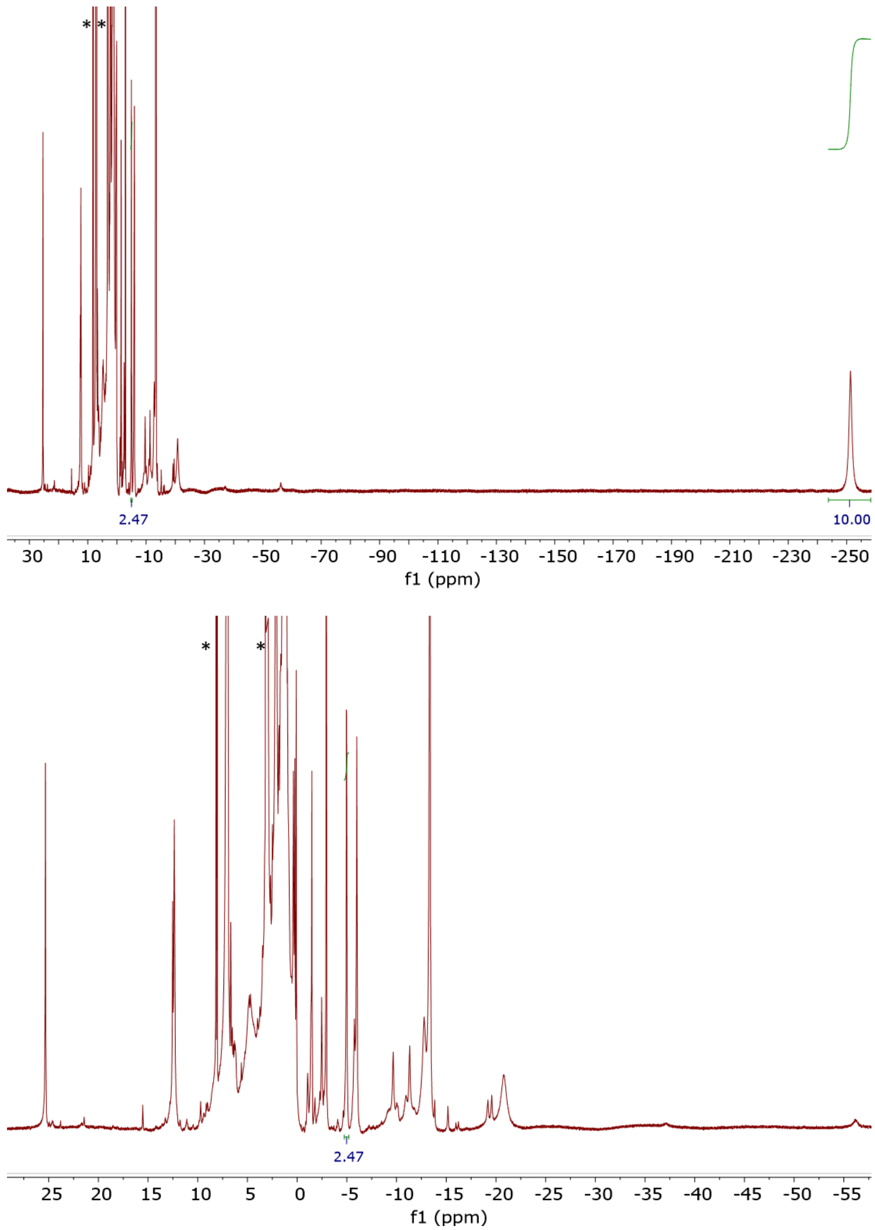

Figure S7. ^1H NMR (500 MHz) spectrum taken after stirring **$NaFe_4S_4$** and 3.1 equiv of IPrNi(toluene) at ambient temperature for 2 hours in toluene (Table S1, Trial 5). A zoomed in spectrum is shown on the bottom. The * denotes residual protiated solvent peaks of C_7D_7H (7.09 and 2.09 ppm). A yield of **$Ni_2Fe_3S_4$** was determined by comparing the observed concentration (1.9 mM) compared to the theoretical concentration (9.0 mM) using a $NiCp_2$ capillary (9.4 mM).

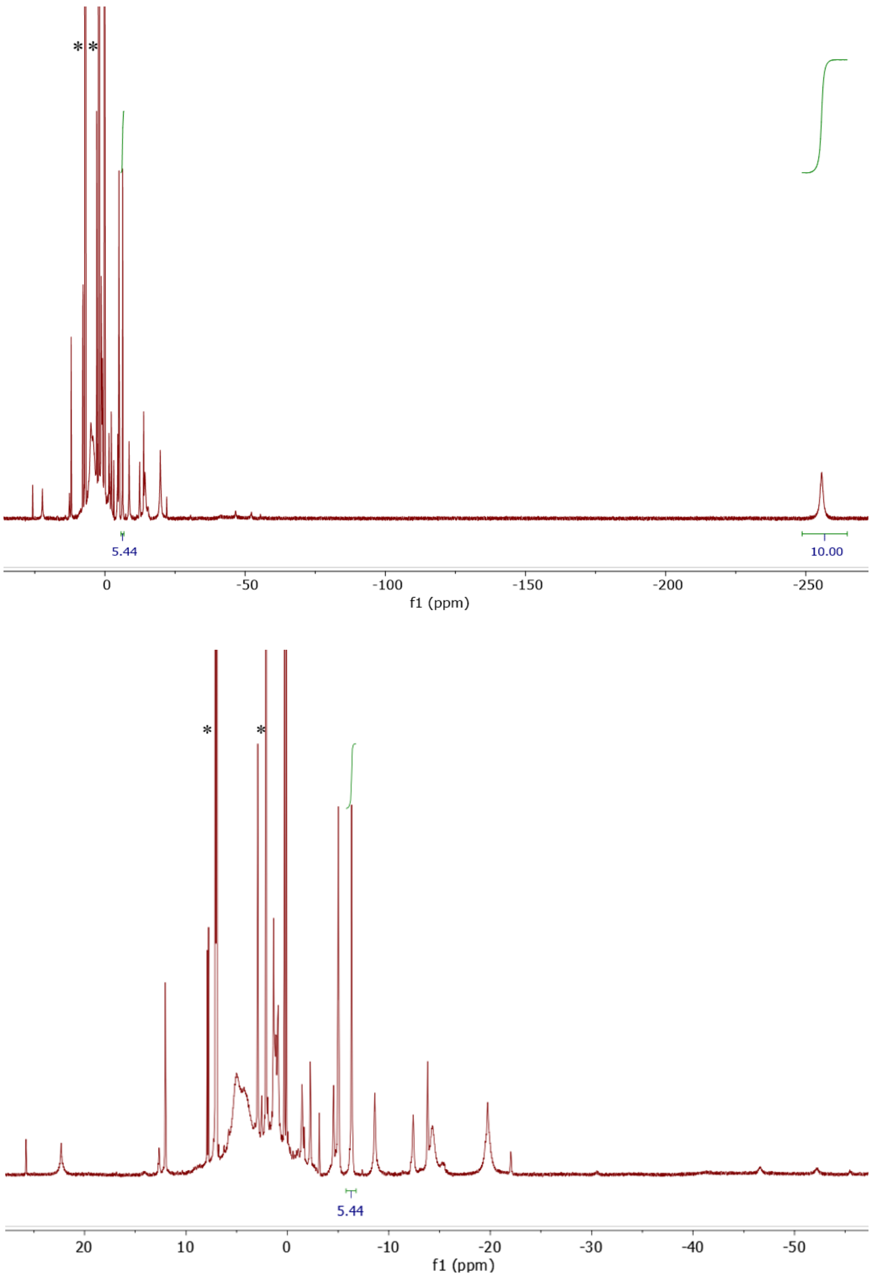

Figure S8. ^{1}H NMR (500 MHz) spectrum taken after stirring **$NaFe_4S_4$** and 2.0 equiv. of IPrNi(toluene) at ambient temperature for 60 minutes in toluene/THF (Table S1, Trial 7). A zoomed in spectrum is shown on the bottom. The * denotes residual protiated solvent peaks of C_7D_7H (7.09 and 2.09 ppm).

FTIR spectra of metal complexes

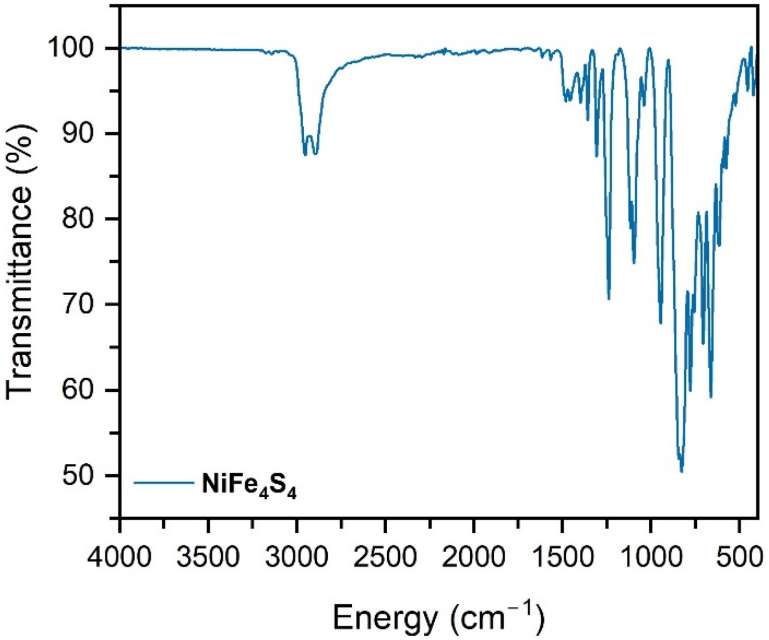

Figure S9. FTIR spectrum of **$NiFe_4S_4$** (solid).

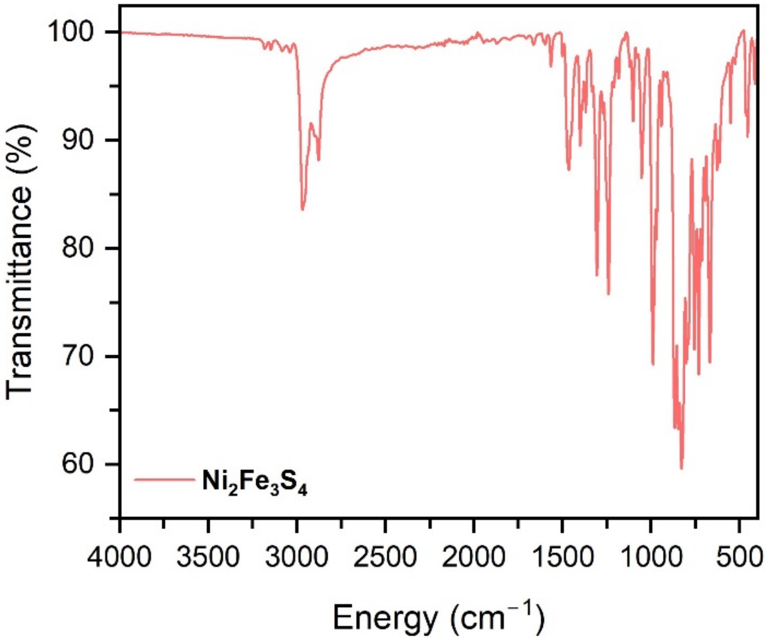

Figure S10. FTIR spectrum of **$Ni_2Fe_3S_4$** (solid).

UV/vis spectra of metal complexes

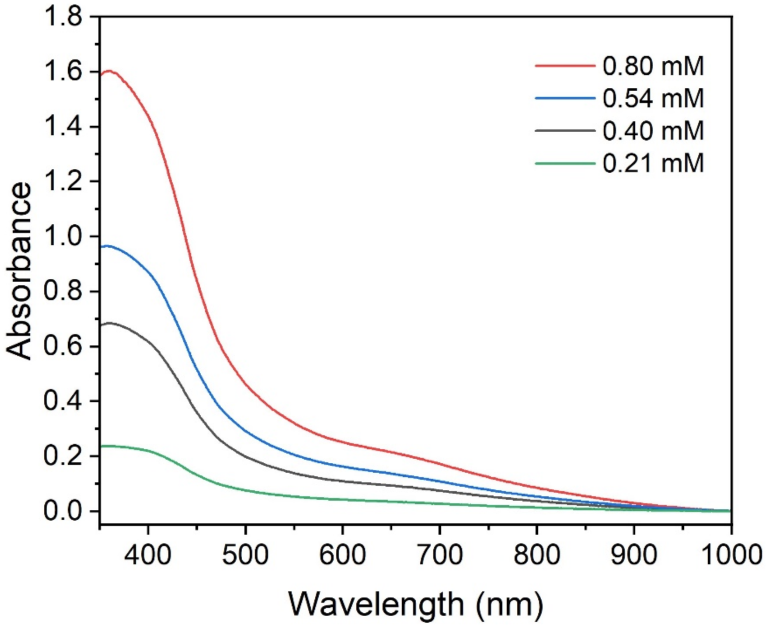

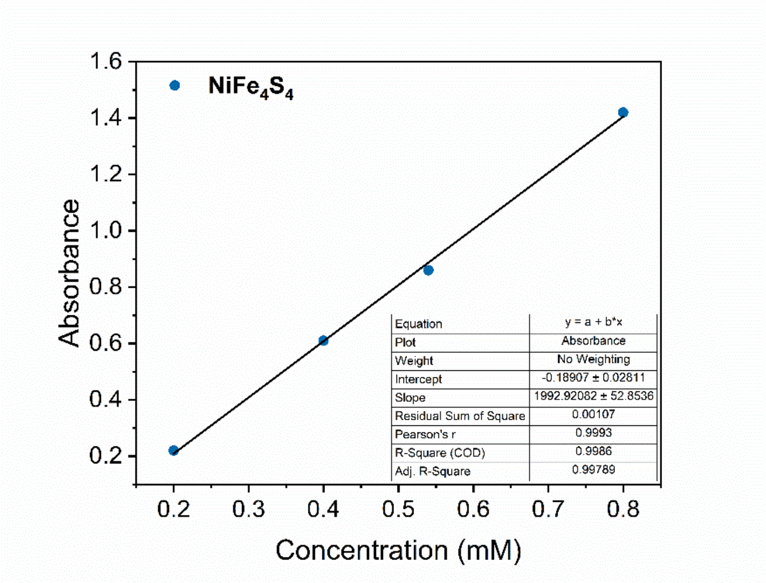

Figure S11. Top: UV-vis spectra of **$NiFe_4S_4$** in 0.21-0.8 mM solutions in benzene. Bottom: Beer-Lambert Law plot of selected UV-vis spectral feature at 400 nm.

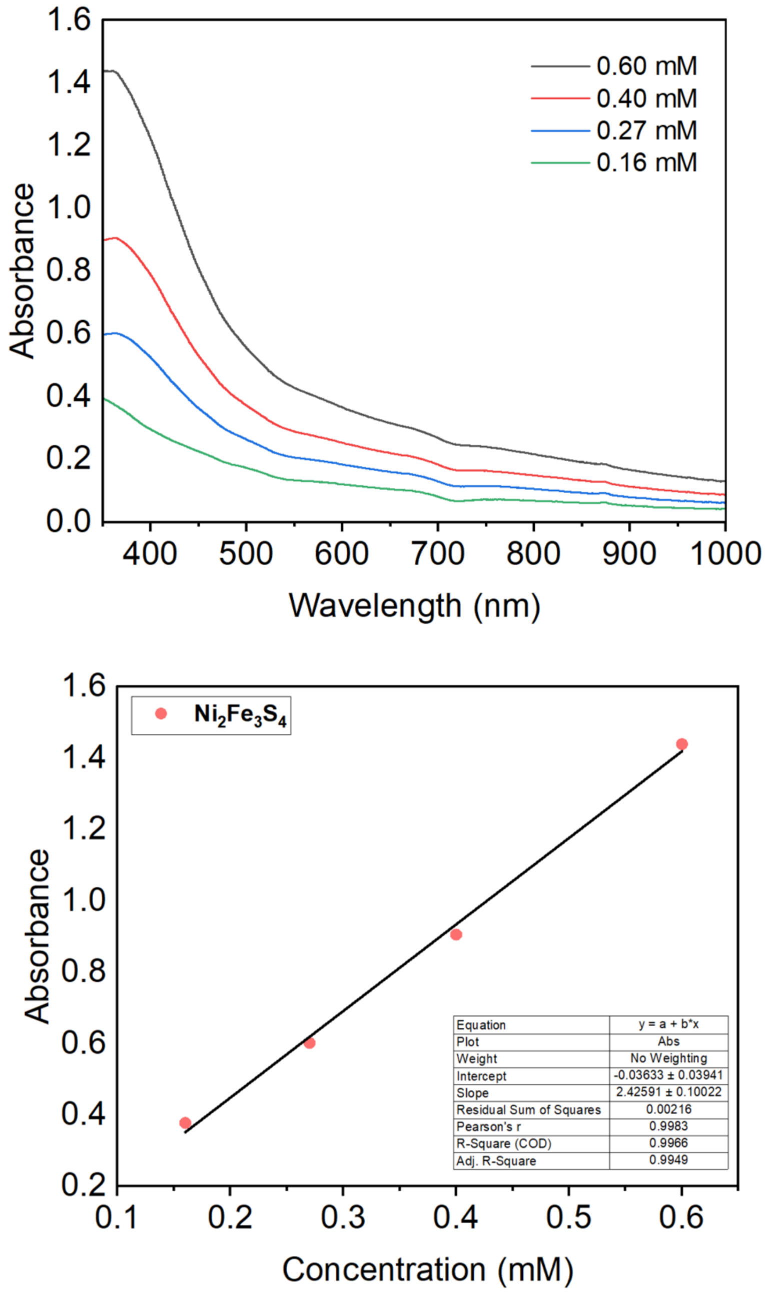

Figure S12. Top: UV-vis spectra of $\mathbf{Ni_2Fe_3S_4}$ in 0.16-0.6 mM solutions in benzene. Bottom: Beer-Lambert Law plot of selected UV-vis spectral feature at 361 nm.

Mössbauer spectra and assignments

Mössbauer spectra were recorded on a conventional spectrometer with an alternating constant acceleration of the γ-source. The minimum experimental linewidth was 0.24 mm s^{-1} (full-width at half-maximum). The sample temperature was controlled using an Oxford Instruments Variox cryostat. The ^{57}Co/Rh source (0.6 GBq) was kept at room temperature. Isomer shifts are quoted relative to iron metal at 300 K. The data were collected without an external magnetic field.

Table S2. Zero-field Mössbauer fitting parameters for **$NaFe_4S_4$**, **$NiFe_4S_4$**, and **$Ni_2Fe_3S_4$**.

Complex	Component	Isomer shift (δ, mm s^{-1})	Quadrupole Splitting ($\lvert\Delta E_Q\rvert$, mm s^{-1})	FWHM (Γ, mm s^{-1})	Area
**$NaFe_4S_4$**, 1-site	1	0.32	1.28	0.37	100%
**$NaFe_4S_4$**, 2-site	1	0.31	1.37	0.34	75%
	2	0.36	1.06	0.25	25%
**$NiFe_4S_4$**	1	0.46	1.03	0.56	100%
**$Ni_2Fe_3S_4$**, 1-site	1	0.48	0.82	0.36	100%
**$Ni_2Fe_3S_4$**, 2-site	1	0.34	0.75	0.34	33%
	2	0.52	0.83	0.28	67%
**IPrNiFe$_4$S$_4$**[*]	NA	0.47	1.13	0.81	

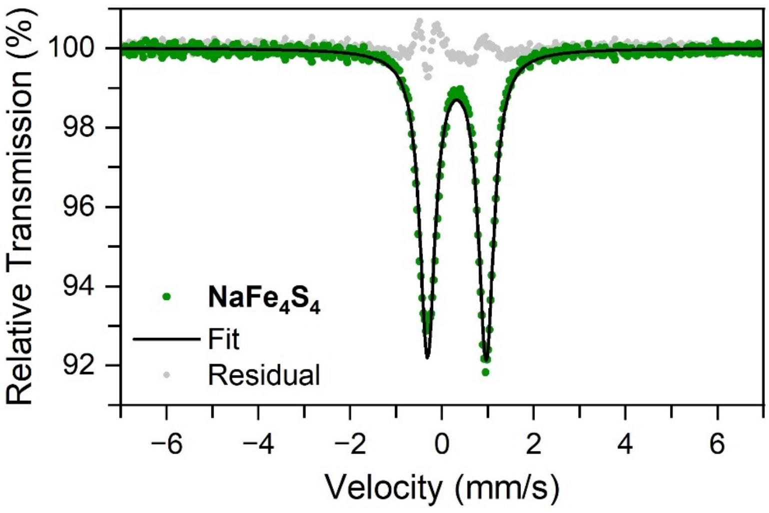

Figure S13. Zero-field Mössbauer spectrum of **$NaFe_4S_4$** at 80 K. Black lines are best fit to the data, and gray lines represent the residuals. The data were fit using a single site model with the following parameters: δ = 0.32 mm/s, ΔE_Q = 1.28 mm/s, and Γ = 0.37 mm/s.

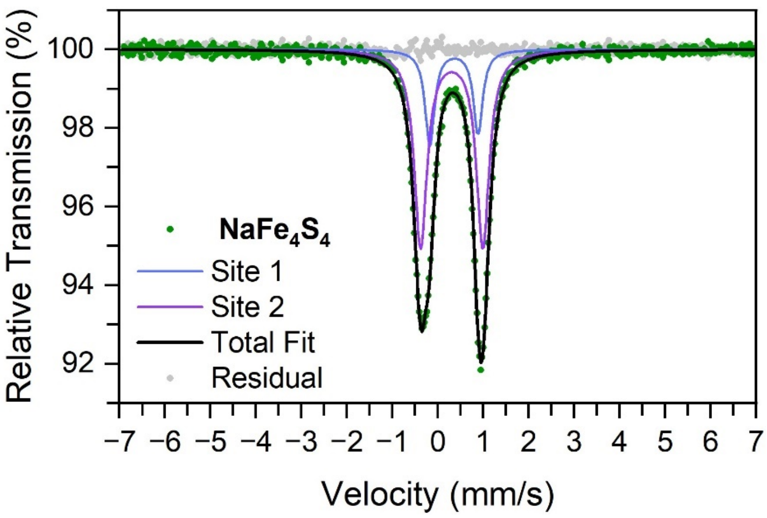

Figure S14. Zero-field Mössbauer spectrum of **NaFe$_4$S$_4$** at 80 K. Black lines are best fit to the data, and gray lines represent the residuals. The data were fit using a two-site model with the following parameters: δ_1 = 0.31 mm/s, ΔE_{Q1} = 1.37 mm/s, and Γ_1 = 0.34 mm/s, area = 75%; δ_2 = 0.36 mm/s, ΔE_{Q2} = 1.06 mm/s, and Γ_2 = 0.25 mm/s, area = 25%. The two-site model gives a better residual and is more consistent with the iron oxidation states (1 Fe^{2+} and 3 Fe^{3+}). The relative areas of the two components were restrained during the fitting.

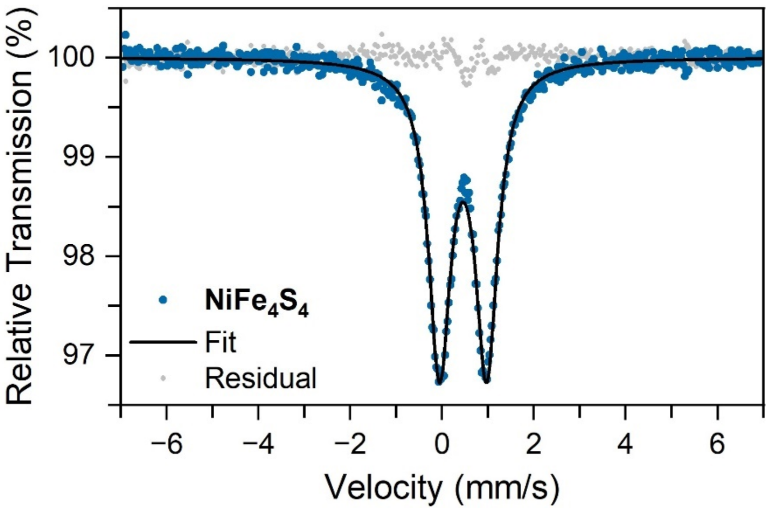

Figure S15. Zero-field Mössbauer spectrum of **NiFe$_4$S$_4$** at 80 K. The black line is the best fit to the data, and grey circles represent the residual. The data were fit using a one-site model with the following parameters: δ = 0.46 mm/s, ΔE_Q = 1.03 mm/s, and Γ = 0.56 mm/s. Attempts to fit the data with two sites did not improve the quality of the fit.

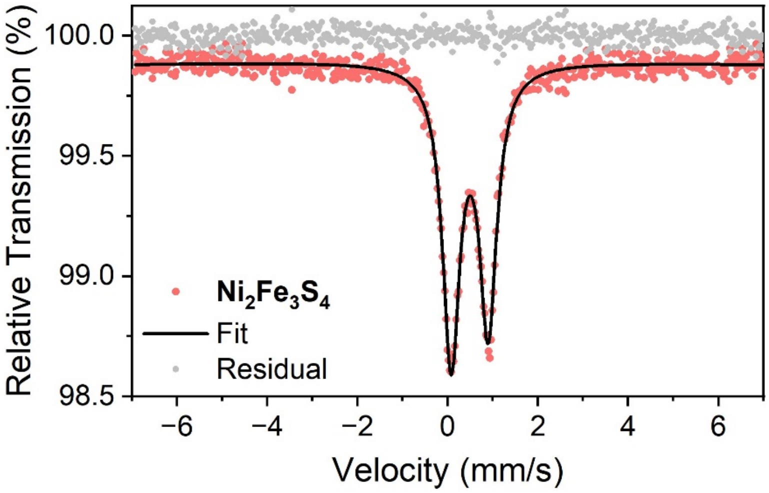

Figure S16. Zero-field Mössbauer spectrum of $\mathbf{Ni_2Fe_3S_4}$ at 80 K. The black line is the best fit to the data, and gray circles represent the residual. The data were fit using a one-site model with the following parameters: δ = 0.48 mm/s, ΔE_Q = 0.82 mm/s, and Γ = 0.36 mm/s.

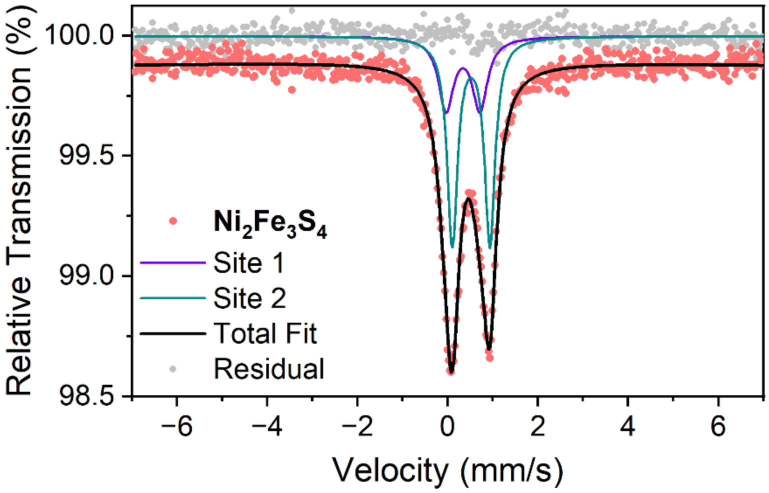

Figure S17. Zero-field Mössbauer spectrum of $\mathbf{Ni_2Fe_3S_4}$ at 80 K. The black line is a best fit to the data, and gray circles represent the residual. The data were fit using a two-site model while restraining the relative area of the two components to a 1:2 ratio. The fit gave the following parameters: δ_1 = 0.34 mm/s, ΔE_{Q1} = 0.75 mm/s, and Γ_1 = 0.34 mm/s, area = 33%; δ_2 = 0.52 mm/s, ΔE_{Q2} = 0.83 mm/s, and Γ_2 = 0.28 mm/s, area = 67%.

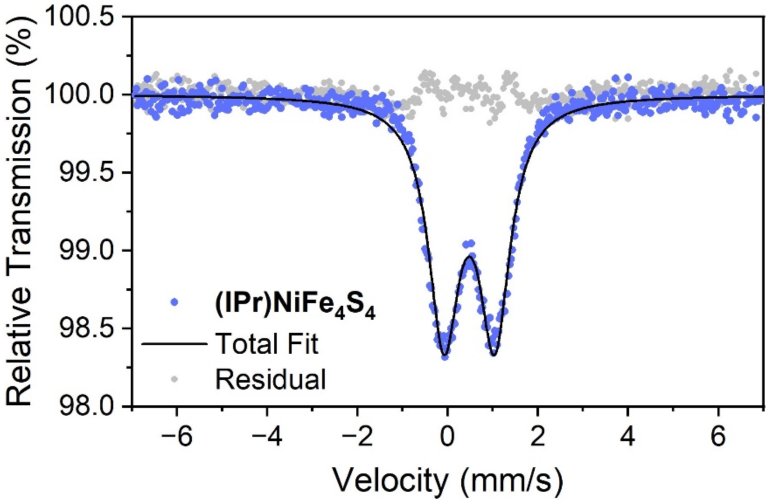

Figure S18. Zero-field Mössbauer spectrum of **IPrNiFe$_4$S$_4$** at 80 K. The black line is a best fit to the data, and gray circles represent the residual. The data were fit using a one-site model with the following parameters: δ = 0.47 mm/s, ΔE_Q = 1.13 mm/s, and Γ = 0.81 mm/s.

EPR spectra

Continuous-wave EPR spectra were recorded at X-band frequency (9.375–9.379 GHz) in perpendicular mode using a Bruker ELEXSYS EPR Spectrometer equipped with an ER 049X microwave bridge and SHQ resonator. The data were collected using the following parameters: modulation frequency of 100 KHz, modulation amplitude of 2.0 G, time constant of 2.56 ms, sweep time of 41.94 ms, and microwave power of 0.64 mW (**$NaFe_4S_4$**), 0.2 mW (**$NiFe_4S_4$** and **$Ni_2Fe_3S_4$**), and 0.16 mW for **$IPrNiFe_4S_4$**. The spectra were simulated using the pepper function in EasySpin[7] with the spin Hamiltonian $\hat{H} = (g_x+g_y+g_z)\mu_B \boldsymbol{SH}$ using S = 1/2 for both **$NaFe_4S_4$** and **$NiFe_4S_4$**. The spectrum for **$IPrNiFe_4S_4$** was simulated using a two-component fit using the parameters in **Table S3**. The simulation for **$NiFe_4S_4$** used a Voigtian lineshape with a 4.47 mT Gaussian component and a 1.2 Lorentzian component and included *g*-strain of [0.08 0.0 0.005]. The simulation of **$Ni_2Fe_3S_4$** used a Voigtian lineshape with 7 mT Gaussian contribution, and a 3 mT Lorentzian contribution with a *g*-strain of 0.005.

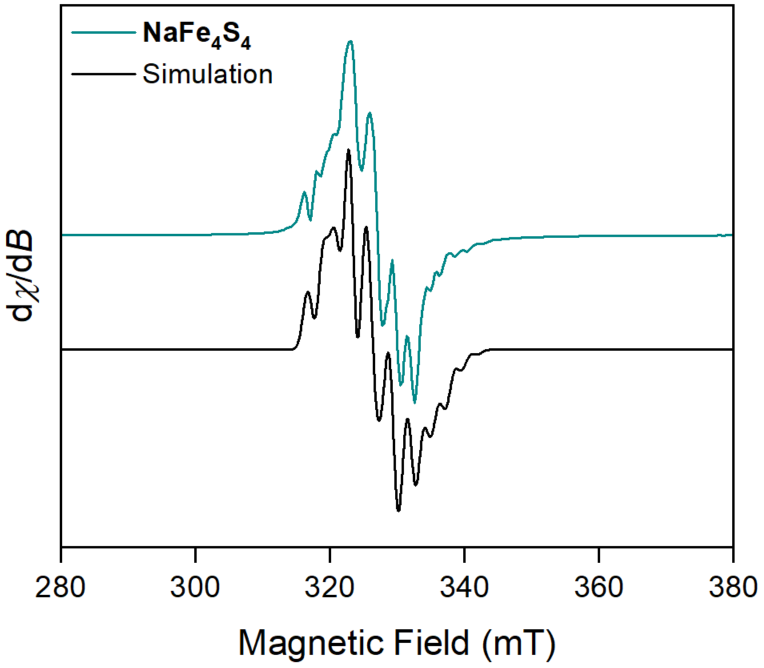

Figure S19. X-band EPR spectrum of a 1 mM solution (in toluene) of **$NaFe_4S_4$** at 9.379 GHz. The rhombic spectrum was fit with g = [2.074, 2.052, 2.014], A (3 x ^{14}N) = [60, 85, 66] MHz, and A (1 x ^{14}N) = [0 0 65]. The simulation used a Gaussian lineshape of 1.7 mT.

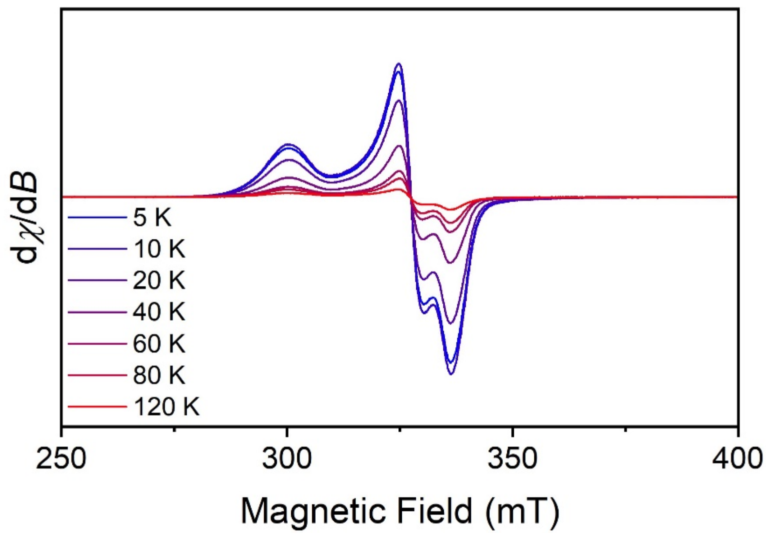

Figure S20. Variable temperature X-band EPR spectrum of a 1 mM solution (in toluene) of **$NiFe_4S_4$** at 9.376 GHz.

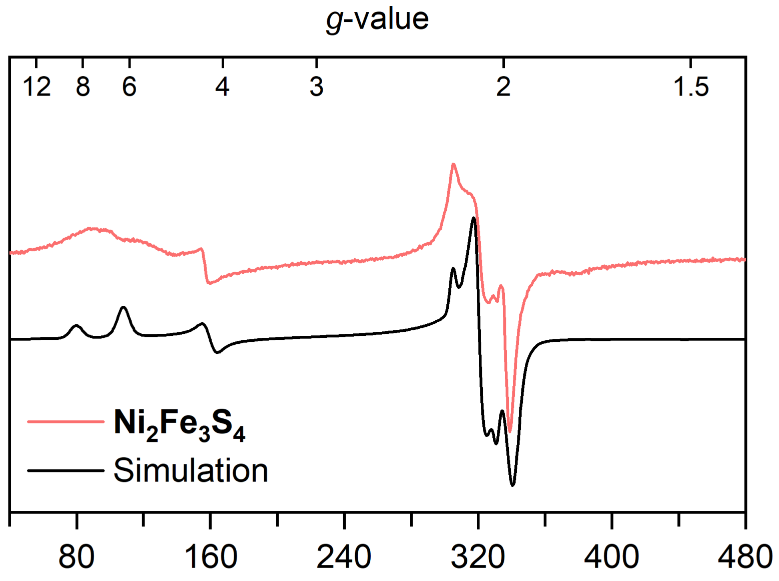

Figure S21. Solid-state X-band (9.381 GHz) EPR spectrum of **$Ni_2Fe_3S_4$** at 10 K. The spectrum was fit with $g = [2.11, 2.15, 2.15]$, $D = -9.0\ cm^{-1}$ and $E = -0.765\ cm^{-1}$. The simulation accounts for a $S = 5/2$ component (99.8%) and a $S = 1/2$ component (0.2%). The $S = 5/2$ component was simulated using a Voigtian lineshape with 7 mT Gaussian contribution, and a 3 mT Lorentzian contribution with a g-strain of 0.005. The $S = ½$ component was modeled using a Gaussian lineshape of 4 mT.

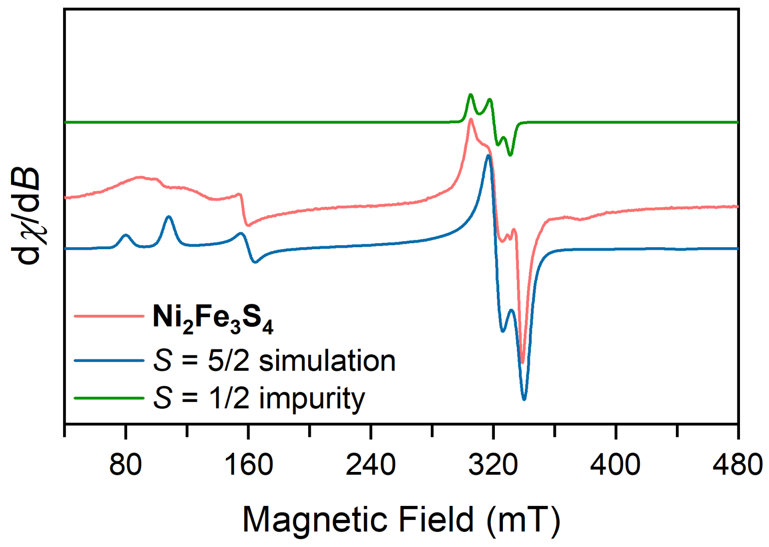

Figure S22. Simulation of the X-band EPR spectrum (red) of $\mathbf{Ni_2Fe_3S_4}$ at 9.381 GHz. The simulation is deconvoluted into the $S = 5/2$ component (blue) and the $S = 1/2$ impurity (green). The relative contribution of the $S = ½$ component to the total simulation was modeled at 0.2%.

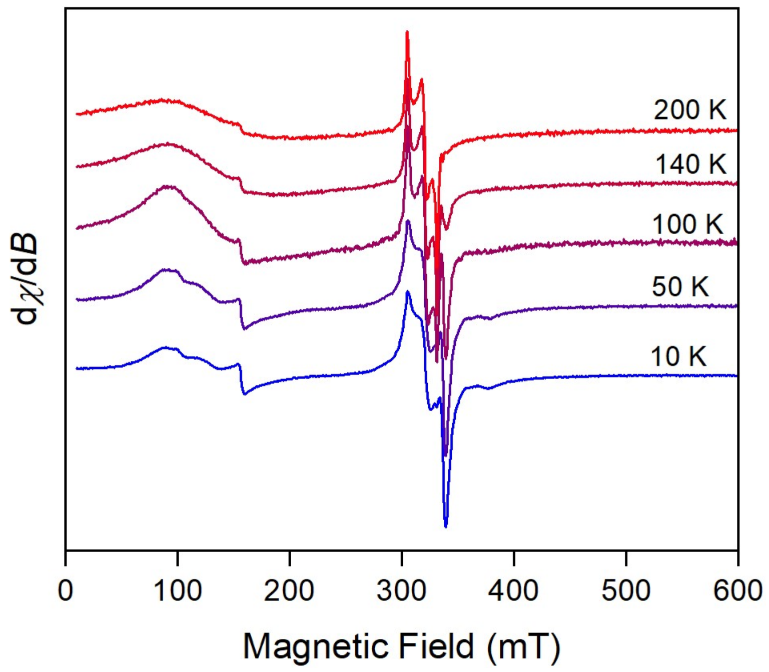

Figure S23. Variable temperature X-band EPR spectrum of $\mathbf{Ni_2Fe_3S_4}$ at 9.381 GHz. The $S = 5/2$ component gradually diminishes as the temperature increases, leaving only the $S = 1/2$ component resolved at higher temperatures.

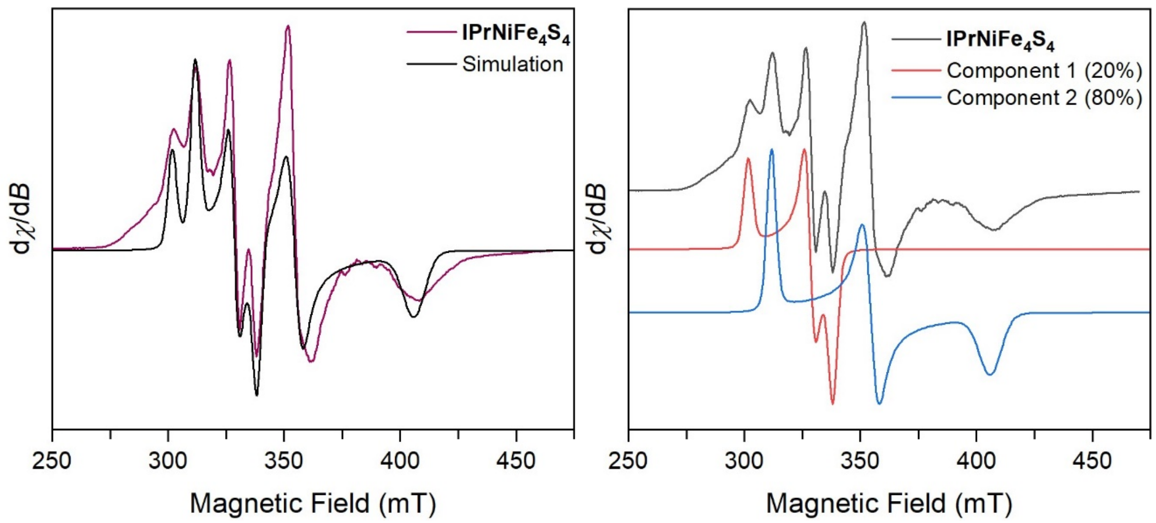

Figure S24. X-band (9.380 GHz) EPR spectrum of a 1 mM solution (in toluene) of **$IPrNiFe_4S_4$**. The left figure shows the total simulated spectrum as a weighted average of the two components. The right figure shows the total simulated spectrum deconvoluted into its two components.

Table S3. EPR fitting parameters for **$NaFe_4S_4$**, **$NiFe_4S_4$**, **$Ni_2Fe_3S_4$**, and **$IPrNiFe_4S_4$**.

Compound	*S*	*g*	*A* (^{14}N, in MHz)	*D* (*E*/*D*) (in cm^{-1})
**$NaFe_4S_4$**	1/2	2.074, 2.052, 2.014	3 × [60, 85, 66]	-
			1 × [0, 0, 65]	
**$NiFe_4S_4$**	1/2	2.23, 2.05, 1.99	-	-
**$Ni_2Fe_3S_4$**	5/2	2.11, 2.15, 2.15	-	−9.0 (0.09)
**$Ni_2Fe_3S_4$ (impurity)**	1/2	2.20 2.09 2.02	-	-
**$IPrNiFe_4S_4$** (comp 1, 20%)	1/2	2.22, 2.04, 1.98	-	-
**$IPrNiFe_4S_4$** (comp 2, 80%)	1/2	2.15, 1.89, 1.65	-	-

Quantitative EPR Studies

We were interested in following the formation of **$IPrNiFe_4S_4$** spectroscopically. To do so, 1 mL of a solution (10 mM) of IPrNi(η^4-C_6H_{10}) in toluene was added dropwise to 1 mL of a solution (10 mM) of **$NaFe_4$** in toluene. The solution was allowed to stand for 10 minutes before 0.25 mL of the solution was added to an EPR tube, frozen, and a spectrum was recorded. The spectrum and double integration are shown below (Figure S24 and S26). The double integration of a solution (5 mM) of **$NaFe_4$** in toluene is shown below (Figure S25). The final conversion to EPR active species (**$IPrNiFe_4S_4$**) is 33%. The derivative

spectrum could be fit using the parameters listed in Figure S16 to give a final yield of 7% for component 1 and 30% for component 2.

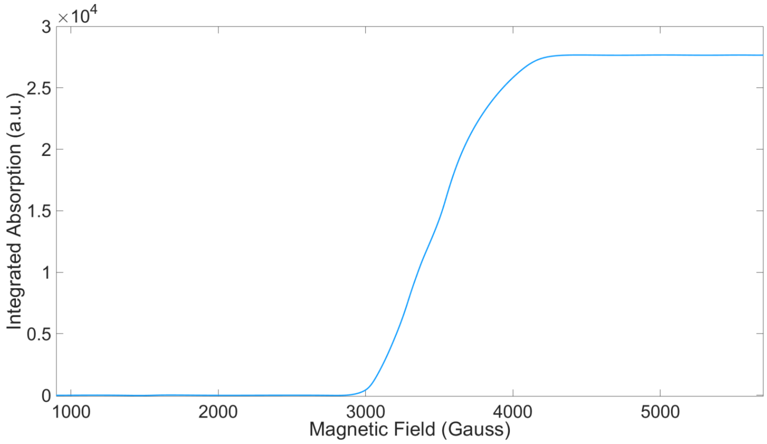

Figure S25. Double integral of X-band EPR spectrum of a 5 mM solution (in toluene) of **$IPrNiFe_4$**. The max y-value is $2.77x10^4$.

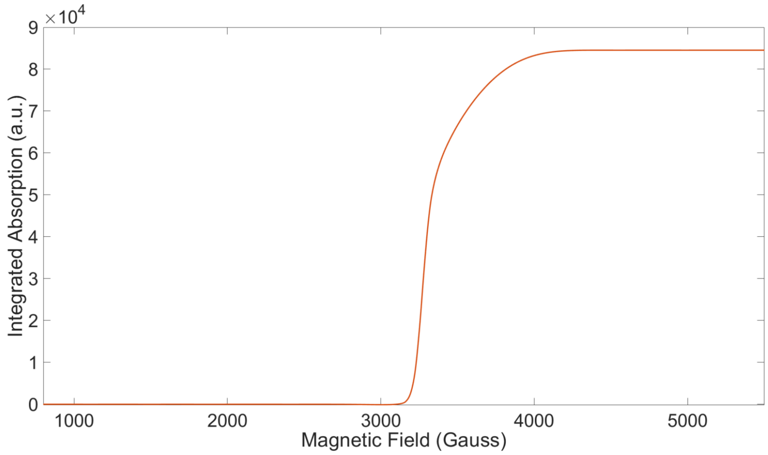

Figure S26. Double integral of X-band EPR spectrum of a 5 mM solution (in toluene) of **$NaFe_4$**. The maximum y-value is 8.45×10^4.

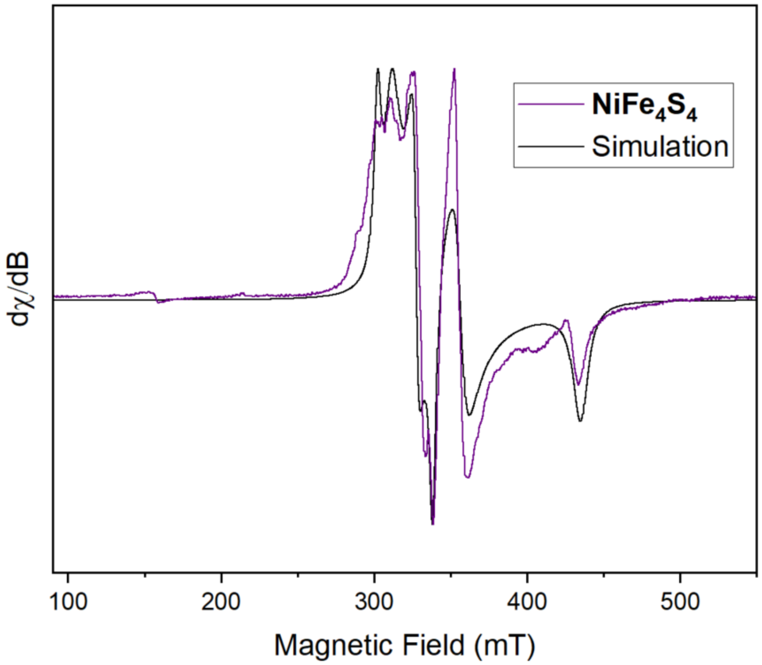

Figure S27. Derivative spectrum of EPR active species resulting from mixing **$NaFe_4S_4$** and IPrNi(η^4-C_6H_{10}).

Magnetic measurements

Magnetic susceptibility data were collected using a Quantum Design MPMS 3 superconducting quantum interference device (SQUID) magnetometer. Magnetic measurements for compounds **$NaFe_4S_4$**, **$NiFe_4S_4$** and **$Ni_2Fe_3S_4$** were performed on ground microcrystalline solids. The samples were prepared under an atmosphere of N_2 and restrained with eicosane in polyethylene capsules. DC magnetic measurements were collected in the temperature range of 2–300 K. Variable field magnetization curves were collected at 100 K to check for curvature indicative of the presence of ferromagnetic impurities. DC magnetic susceptibility measurements were performed under applied magnetic fields of 0.05 T and 0.10 T, and 0.5 T and corrected for the diamagnetism of each sample and eicosane, estimated using Pascal's constants.[8] Variable field, variable temperature magnetization measurements (reduced magnetization) were performed under applied magnetic fields of 1–7 T in 1 T increments, in the temperature range of 2–10 K. DC magnetic susceptibility data and reduced magnetization data were simulated using the program MagProp in DAVE 2.0.[9] Magnetic data for **$Ni_2Fe_3S_4$** were modelled according to the Van Vleck model[10] using the following spin Hamiltonian: $\hat{H} = D\hat{S}_z^2 + E(\hat{S}_x^2 - \hat{S}_y^2) + g_i \mu_B \boldsymbol{SH}$, ($i = x, y, z$). In this Hamiltonian, D and E are the axial and transverse zero-field splitting parameters, respectively, g is the electron g-value, S is the spin operator, and $\boldsymbol{H}$ is the magnetic field.

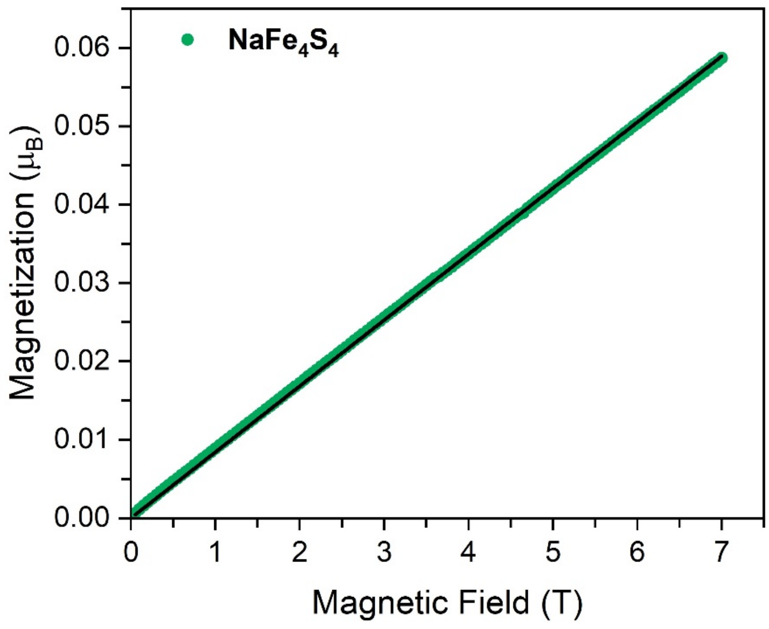

Figure S28. Variable field magnetization collected on a polycrystalline sample of **NaFe4S**$_4$ at 100 K. The linear fit and absence of curvature supports the absence of significant ferromagnetic impurities.

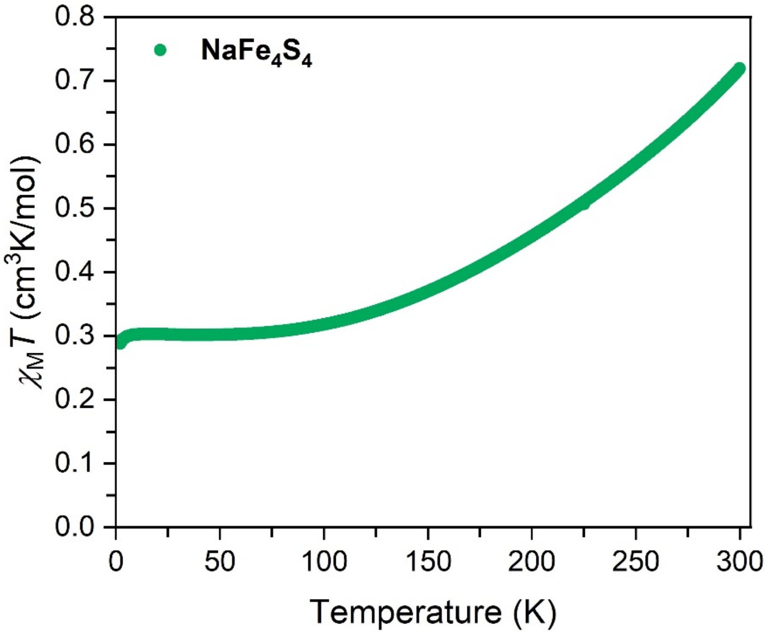

Figure S29. Variable temperature magnetic susceptibility data collected on a polycrystalline sample of **NaFe**$_4$**S**$_4$ at 5000 Oe. The low temperature $\chi_M T$ value of 0.3 cm^{3}K/mol is consistent with a S = 1/2 ground state and is in agreement with its X-band EPR spectrum.

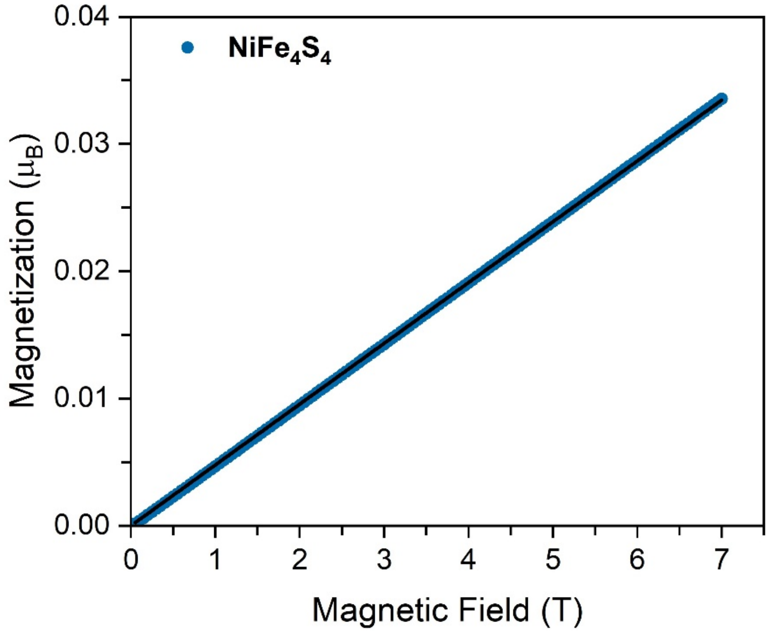

Figure S30. Variable field magnetization collected on a polycrystalline sample of $\mathbf{NiFe_4S_4}$ at 100 K. The linear fit and absence of curvature supports the absence of significant ferromagnetic impurities.

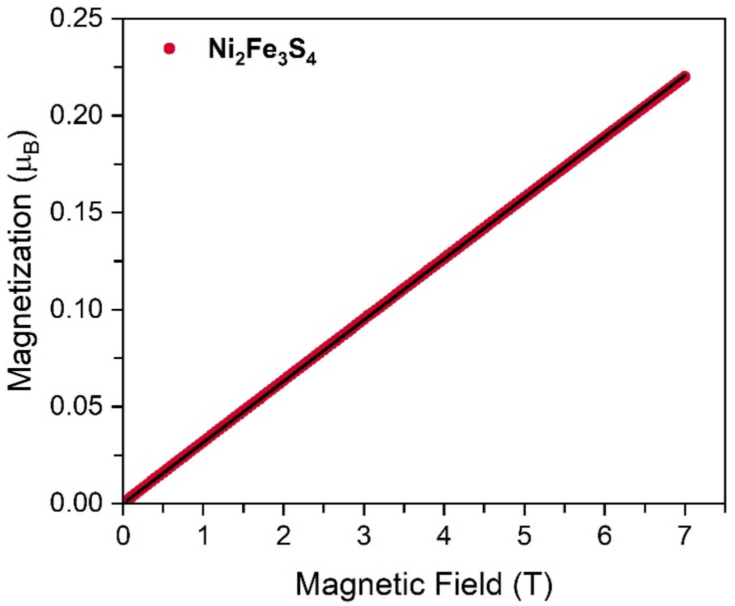

Figure S31. Variable field magnetization collected on a polycrystalline sample of $\mathbf{Ni_2Fe_3S_4}$ at 100 K. The linear fit and absence of curvature supports the absence of significant ferromagnetic impurities.

Cyclic voltammetry data

Cyclic voltammetry measurements were performed inside an argon filled glovebox, and were collected using a CHI 660E potentiostat in an undivided three-electrode cell that used a glassy carbon working electrode, a Pt wire counter electrode, and an Ag wide reference electrode. A 0.1 M $[Bu_4N][PF_6]$ electrolyte solution in THF was used for all measurements, and ferrocene was added after initial data collection as an internal reference.

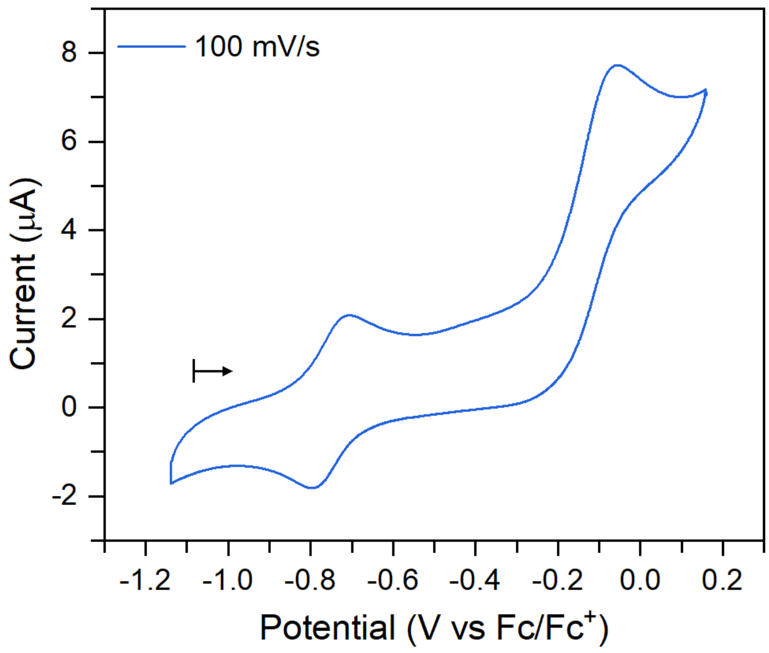

Figure S32. Cyclic voltammogram of **$NiFe_4S_4$**. The CV shows a quasi-reversible redox process at E = –0.75 V (versus Fc/Fc^+) and an irreversible redox process at E = –0.08 V. The CV was collected at a scan rate of 100 mV/s.

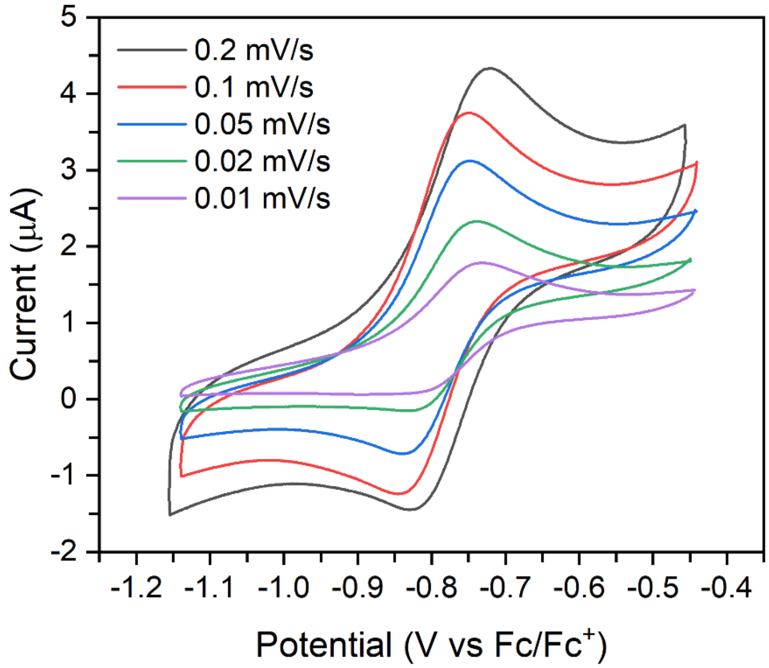

Figure S33. Variable scan rate CV of **$NiFe_4S_4$**.

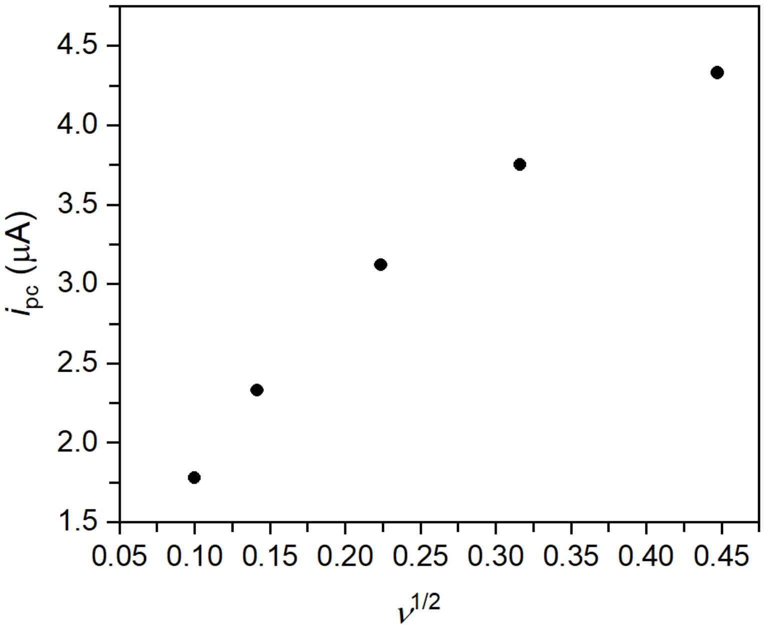

Figure S34. Dependence of the cathodic peak current on the square root of the scan rate ($\nu^{1/2}$) for the redox process at –0.75 V. The plot is non-linear and therefore we classify it as quasireversible.

Crystallographic data

Low-temperature diffraction data (ω-scans) were collected on a Rigaku MicroMax-007HF diffractometer coupled to a Saturn994+ CCD detector with Cu Kα (λ = 1.54178 Å) for the structure of 007a-22087. The diffraction images were processed and scaled using Rigaku Oxford Diffraction software (CrysAlisPro; Rigaku OD: The Woodlands, TX, 2015). The structure was solved with SHELXT and was refined against F^2 on all data by full-matrix least squares with SHELXL (Sheldrick, G. M. *Acta Cryst.* **2008**, *A64*, 112–122). All non-hydrogen atoms were refined anisotropically. Hydrogen atoms were included in the model at geometrically calculated positions and refined using a riding model. The isotropic displacement parameters of all hydrogen atoms were fixed to 1.2 times the U value of the atoms to which they are linked (1.5 times for methyl groups). The full numbering scheme of compounds **$NiFe_4S_4$** and **$Ni_2Fe_3S_4$** can be found in the full details of the X-ray structure determination (CIF), which is included as Supporting Information. CCDC numbers 2356745 (**$NiFe_4S_4$**) and 2356746 (**$Ni_2Fe_3S_4$**) contains the supplementary crystallographic data for this paper. These data can be obtained free of charge from The Cambridge Crystallographic Data Center via www.ccdc.cam.ac.uk/data_request/cif.

Refinement details for $NiFe_4S_4$: The 15-crown-5 was modeled as disordered over two positions. The site occupancies were freely refined and converged at nearly a 0.9/0.1 split in the population distribution of the major and minor component. All disordered, chemically equivalent C-C and C-O distances were restrained to be similar with strict standard uncertainties of 0.002. Due to the minor component's low occupancy, strict uncertainties were also use to restrain the thermal parameters of the major and minor component to be similar. Finally, several of the C,O and C,C angles converged at chemically unreasonable values and the distances which separate them were restrained to reasonable values (2.25 Å with an uncertainty of 0.002).

Refinement details for $Ni_2Fe_3S_4$: Three THF models were included in the asymmetric unit. All three were disordered. In the disordered models, chemically similar C-C and C-O bonds were restrained to have similar distances. The disordered thermal parameters were collectivity restrained to have similar tensor values. Several 1,3 distances were restrained with target values of 2.415(2) Å. Without these restraints, the THF models converged to chemically unreasonable geometries.

Table S4. Crystallographic M–M distances and formal shortness ratios (FSRs; computed according to Chipman & Berry 2020)[11] for **$NiFe_4S_4$** and **$Ni_2Fe_3S_4$**.

**$NiFe_4S_4$**			**$Ni_2Fe_3S_4$**		
	Distance (Å)	FSR		Distance (Å)	FSR
Ni(1)–Fe1	2.5423(12)	1.16	Ni(1)–Fe(2)	2.5213(13)	1.14
Fe1–Fe2	2.8207(12)	1.28	Ni(1)#1–Fe(2)	2.5214(13)	1.14
Fe1–Fe3	2.8533(12)	1.30	Fe(2)–Fe(1)	2.6325(11)	1.20
Fe1–Fe4	2.7773(12)	1.26	Fe(2)–Fe(1)#1	2.6325(11)	1.20
Fe2–Fe3	2.9111(13)	1.32			
Fe2–Fe4	2.9101(13)	1.32			
Fe3–Fe4	2.9218(13)	1.33			

Table S5. Selected crystallographic distances (Å) and angles (°) for **$NiFe_4S_4$** and **$Ni_2Fe_3S_4$**.

**$NiFe_4S_4$**		**$Ni_2Fe_3S_4$**	
Bond Metric	Distance/Angle	Bond Metric	Distance/Angle
Ni(1)-N(1)	1.902(5)	Ni(1)-C(11)	1.899(8)
Ni(1)-N(1)	1.968(5)	Ni(1)-S(2)	2.182(2)
Ni(1)-S(1)	2.2697(16)	Ni(1)-S(1)	2.182(2)
Fe(1)-N(1)	2.032(4)	Fe(2)-S(2)#1	2.231(2)
Fe(1)-S(3)	2.2641(16)	Fe(2)-S(2)	2.231(2)
Fe(1)-S(2)	2.2754(17)	Fe(2)-S(1)#1	2.283(2)
Fe(1)-S(1)	2.3106(17)	Fe(2)-S(1)	2.283(2)
Fe(2)-N(2)	1.959(5)	Fe(1)-N(12)	1.908(6)
Fe(2)-S(4)	2.2814(17)	Fe(1)-S(2)	2.252(2)
Fe(2)-S(2)	2.3255(19)	Fe(1)-S(1)#1	2.267(2)
Fe(2)-S(1)	2.3586(16)	Na(1)-O(1A)	2.16(3)
Fe(3)-N(3)	1.958(5)	Na(1)-O(1A)#1	2.16(3)
Fe(3)-S(3)	2.3137(17)	Na(1)-O(1B)	2.65(2)
Fe(3)-S(4)	2.3232(17)	Na(1)-O(1B)#1	2.65(2)
Fe(3)-S(1)	2.3764(16)	Na(1)-S(1)	3.035(4)
Fe(4)-N(4)	1.953(5)	Na(1)-S(1)#1	3.035(4)
Fe(4)-S(2)	2.2858(17)	C(11)-Ni(1)-S(2)	119.4(2)
Fe(4)-S(4)	2.2966(18)	C(11)-Ni(1)-S(1)	126.9(2)
Fe(4)-S(3)	2.3094(17)	S(2)-Ni(1)-S(1)	113.53(9)
C(1)-Ni(1)-N(1)	144.0(2)		
C(1)-Ni(1)-S(1)	107.26(17)		
N(1)-Ni(1)-S(1)	108.67(14)		

Table S6. Crystal data and structure refinement for **$NiFe_4S_4$**.

Identification code	007a-22087; CCDC 2346745	
Empirical formula	C59 H126 Fe4 N6 Na Ni O6 S4 Si8	
Formula weight	1673.71	
Temperature	93(2) K	
Wavelength	1.54184 Å	
Crystal system	Monoclinic	
Space group	$P2_1/n$	
Unit cell dimensions	a = 20.5031(5) Å	α= 90°.
	b = 20.5824(4) Å	β= 90.314(2)°.
	c = 20.5668(4) Å	γ = 90°.
Volume	8679.1(3) $Å^3$	
Z	4	
Density (calculated)	1.281 g/cm^3	
Absorption coefficient	7.825 mm^{-1}	
F(000)	3556	
Crystal size	0.100 x 0.100 x 0.020 mm^3	
Crystal color and habit	Black Plate	
Diffractometer	Rigaku Saturn 944+ CCD	
Theta range for data collection	3.035 to 68.239°.	
Index ranges	-24<=h<=24, -24<=k<=24, -24<=l<=24	
Reflections collected	218093	
Independent reflections	15768 [R(int) = 0.1383]	
Observed reflections (I > 2sigma(I))	10107	
Completeness to theta = 67.684°	99.6 %	
Absorption correction	Semi-empirical from equivalents	
Max. and min. transmission	1.00000 and 0.40498	
Solution method	SHELXT-2014/5 (Sheldrick, 2014)	
Refinement method	SHELXL-2014/7 (Sheldrick, 2014)	
Data / restraints / parameters	15768 / 780 / 979	
Goodness-of-fit on F^2	1.015	
Final R indices [I>2sigma(I)]	R1 = 0.0696, wR2 = 0.1540	
R indices (all data)	R1 = 0.1178, wR2 = 0.1787	
Largest diff. peak and hole	1.039 and -0.988 e.$Å^{-3}$	

Table S7. Crystal data and structure refinement for **$Ni_2Fe_3S_4$**.

Identification code	007b-22038; CCDC 2346746	
Empirical formula	C90 H156 Fe3 N6 Na Ni2 O6 S4 Si4	
Formula weight	1966.76	
Temperature	93(2) K	
Wavelength	1.54184 Å	
Crystal system	Monoclinic	
Space group	I2/a	
Unit cell dimensions	a = 26.8811(8) Å	α= 90°.
	b = 12.3676(4) Å	β= 97.658(3)°.
	c = 31.7007(12) Å	γ = 90°.
Volume	10445.0(6) $Å^3$	
Z	4	
Density (calculated)	1.251 g/cm^3	
Absorption coefficient	5.265 mm^{-1}	
F(000)	4204	
Crystal size	0.080 x 0.030 x 0.010 mm^3	
Crystal color and habit	Black Plate	
Diffractometer	Rigaku Saturn 944+ CCD	
Theta range for data collection	2.813 to 66.595°.	
Index ranges	-31<=h<=31, -14<=k<=14, -36<=l<=37	
Reflections collected	133298	
Independent reflections	9196 [R(int) = 0.2950]	
Observed reflections (I > 2sigma(I))	4619	
Completeness to theta = 66.595°	99.6 %	
Absorption correction	Semi-empirical from equivalents	
Max. and min. transmission	1.00000 and 0.88380	
Solution method	SHELXT-2014/5 (Sheldrick, 2014)	
Refinement method	SHELXL-2014/7 (Sheldrick, 2014)	
Data / restraints / parameters	9196 / 662 / 676	
Goodness-of-fit on F^2	1.012	
Final R indices [I>2sigma(I)]	R1 = 0.0849, wR2 = 0.1921	
R indices (all data)	R1 = 0.1712, wR2 = 0.2387	
Extinction coefficient	n/a	
Largest diff. peak and hole	0.754 and -0.433 e.$Å^{-3}$	

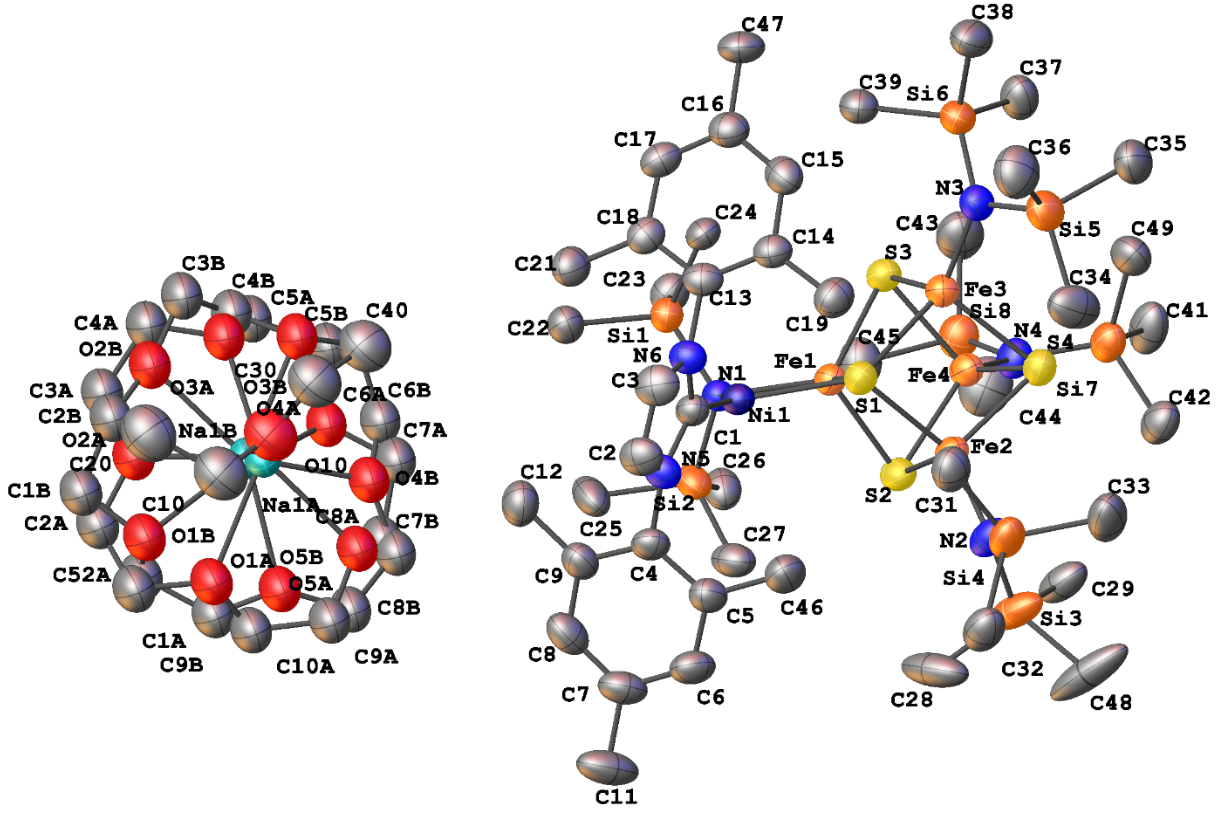

Figure S35. Structure of **$NiFe_4S_4$**. The thermal ellipsoids are drawn at 50% probability level. The hydrogen atoms are omitted for clarity.

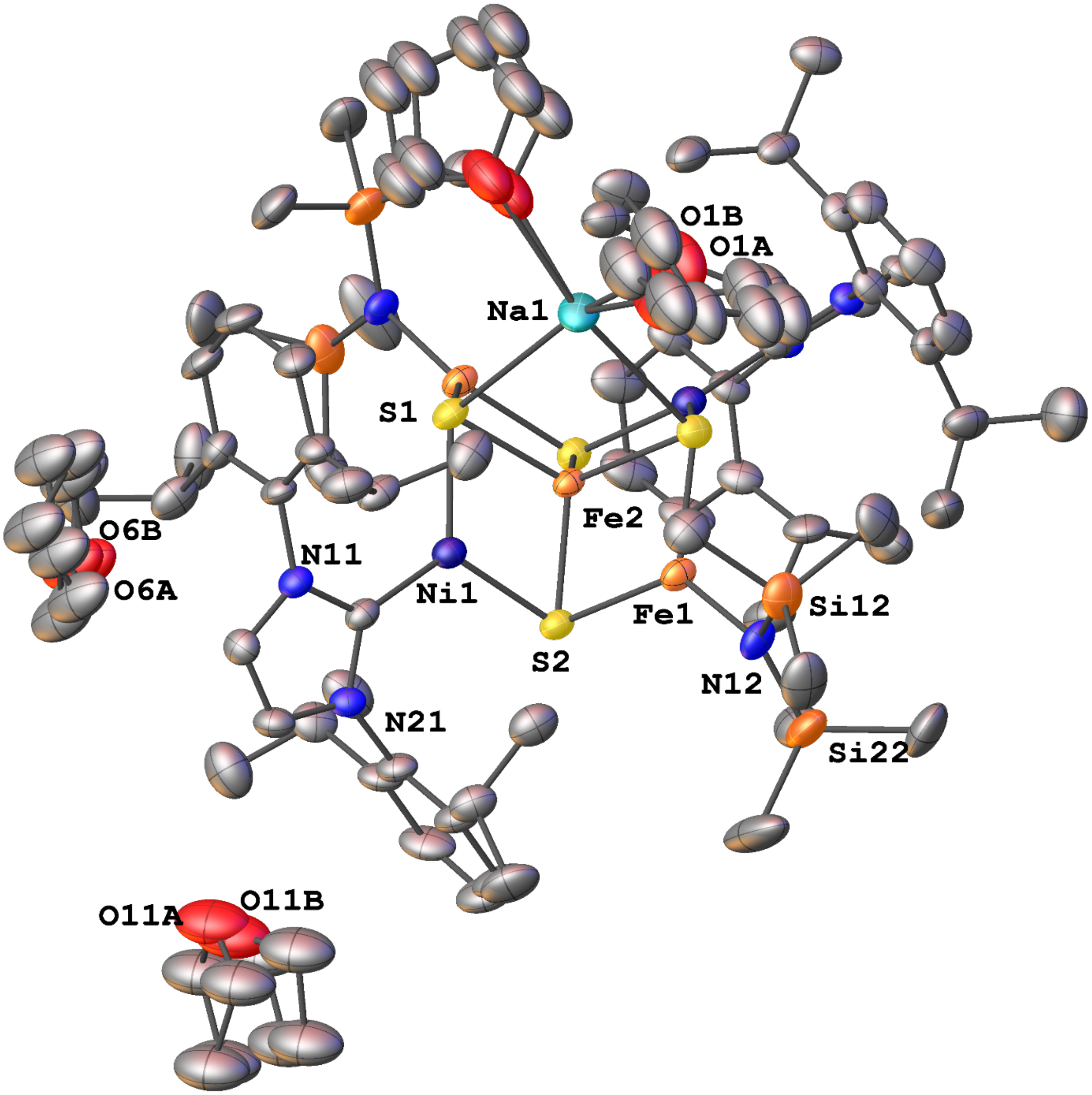

Figure S36. Structure of **$Ni_2Fe_3S_4$**. The thermal ellipsoids are drawn at 30% probability level. The hydrogen atoms, except H1 on B1, are omitted for clarity.

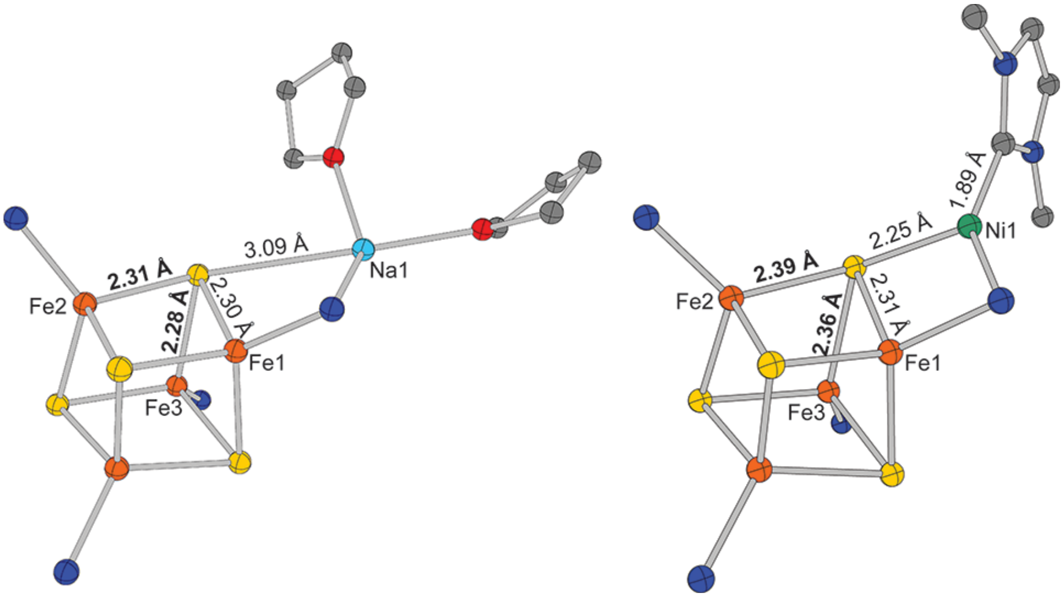

Figure S37. Comparison of the structures of **$NaFe_4S_4$** and **$NiFe_4S_4$**. The thermal ellipsoids are drawn at 30% probability level. Two notable bond lengths, Fe2–S1 and Fe3–S1 are significantly elongated in $NiFe_4S_4$.

Tetrahedral volume determination:

The volumes (V) of the Fe_4- and S_4-core tetrahedra for **$NiFe_4S_4$** was calculated using the Cayley-Menger determinant shown below, where d_{ij} denote the distances between the tetrahedra vertices.

$$288 \cdot V^2 = \begin{vmatrix} 0 & 1 & 1 & 1 & 1 \\ 1 & 0 & d_{12}^2 & d_{13}^2 & d_{14}^2 \\ 1 & d_{12}^2 & 0 & d_{23}^2 & d_{24}^2 \\ 1 & d_{13}^2 & d_{23}^2 & 0 & d_{34}^2 \\ 1 & d_{14}^2 & d_{24}^2 & d_{34}^2 & 0 \end{vmatrix}$$

X-ray Absorption Spectroscopy

X-ray absorption spectroscopic (XAS) measurements of **$Ni_2Fe_3S_4$** and **$NiFe_4S_4$**, as well as the Ni(0) reference complex Ni(IPr)(styrene)$_2$, were performed at beamline 9-3 at the Stanford Synchrotron Radiation Lightsource (SSRL) in 2022 and 2024. SSRL was operated in top-up mode with a ring energy of 3 GeV and a ring current of 500 mA. The incident beam had a nominal flux of 10^{12} photons/second and was monochromatized with a LN_2-cooled Si(220) double-crystal monochromator with φ=0°. Harmonic rejection was achieved with a rhodium-coated mirror, set to a cutoff of 10 keV, and by detuning the monochromator by 20-30%. Aluminum filters totaling 0.25 mm were used to reduce the flux density and the beam spot size was 1x5 mm. Absorbance was measured in transmission mode using three standard ionization chambers filled with N_2, with the sample between the first two chambers and a reference Fe or Ni metal foil between the latter two. Preliminary XAS measurements, which are not presented here, were also performed at beamline P65 at the Deutsches Elektronen- Synchrotron (DESY) in 2022.

Samples for transmission X-ray absorption spectroscopy were prepared by diluting pure powders in boron nitride. Diluted powders were tightly packed into 1-mm-thick aluminum sample cells with polyimide (Kapton) tape windows. Samples were prepared in an O_2- and H_2O-free glovebox and transported to the synchrotron at 77 K in a LN_2 dry shipper. Samples were handled under LN_2 and measured at 10–15 K in a LHe cryostat. Multiple scans were collected at each spot and no evidence of photodamage was observed.

XAS data were processed using Larch v0.9.76 and Python 3.11.[12] The incident beam energy was calibrated to the metal foil edge energies, defined as the first derivative maxima, at 7111.2 eV for the iron K edge[13] and 8331.7 eV for the nickel K edge.[14] Through the edges, data were collected with 0.2 eV (Fe) and 0.25 eV (Ni) spacings, then interpolated onto a 0.01-eV grid. Foil spectra were smoothed with a Whittaker smoother (λ = 5E5) to prior to taking derivatives for energy calibration. Sample spectra were slightly smoothed with a Whittaker smoother (λ = 3E4) to reduce interpolation noise in derivative spectra without affecting the shape of spectra. Data processing was performed with standard procedures in Larch: the pre-edge background was fit with line and subtracted, and the post-edge was fit with a quadratic function and subtracted to obtain “flattened” spectra. All spectroscopic features are identified by maxima and zero-crossings of first derivative spectra.

Calculations using Density Functional Theory

DFT calculations were performed with ORCA v5.0.3[15] using unrestricted determinants, ZORA-Def2-TZVP basis sets,[16] the TPSSh meta-hybrid functional,[17] the zeroth-order regular approximation (ZORA) for relativistic effects,[18,19] the D3BJ dispersion correction,[20,21] and the conductor-like polarizable continuum model[22] (CPCM) of solvation with an infinite dielectric constant. The RIJCOSX approximation[23,24] was used with a SARC/J auxiliary basis[25] to decrease computational costs. Broken-symmetry DFT wavefunctions were characterized by analyzing Pipek-Mezey localized orbitals[26] and Hirshfeld populations.[27] Geometries of all complexes were optimized starting from crystallographic coordinates and all properties were calculated from optimized structures.

DFT Analysis of $NiFe_4S_4$

$NiFe_4S_4$ has a total spin S = ½, determined by EPR spectroscopy and magnetometry. The experimental Mössbauer spectrum and Fe K-edge XAS clearly indicate a $Fe^{2.5+}{}_4S_4$ cluster, implying an assignment of Ni^{1+} that is supported by the preedge in the Ni K-edge XAS. BS DFT analysis indicates Ni^{1+} and a typical $Fe^{2.5+}{}_4S_4$ cubane with two delocalized mixed-valent $Fe^{2.5+}{}_2$ subsystems that are antiferromagnetically (AFM) coupled. All metal 3d orbitals were clearly localized, including a Ni $3d_{x2-y2}$ SOMO and a spin-polarized Ni $3d_{xy}$ DOMO (Figure S38). The unoccupied beta $3d_{x2-y2}$ orbital is the dominant orbital contribution to the first excited state calculated for the Ni K-edge (see Figure S41, left).

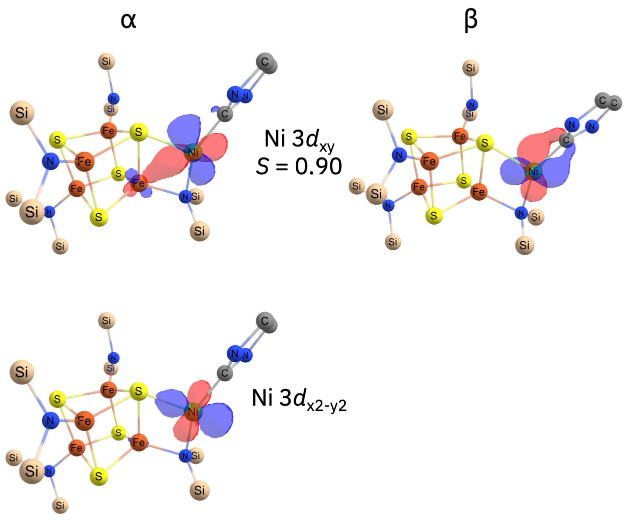

Figure S38. Selected localized Ni 3d orbitals calculated from the BS-03 wavefunction of **$NiFe_4S_4$**; those calculated for BS-01 and BS-02 are equivalent. The z axis is defined as normal to the Ni coordination plane. The Ni $3d_{xy}$ DOMO is spin-polarized with an α/β overlap of 0.90; the α orbital is slightly σ-delocalized to

the adjacent Fe, while the β orbital shows some π-backbonding into the NHC. The Ni 3d_{x2-y2} SOMO has lobes oriented toward ligands, and indicates a Ni^{1+} assignment for the BS wavefunction.

Three different spin coupling schemes were investigated as potential ground states for **$NiFe_4S_4$**. They differ in the arrangement of mixed-valent subsystems within the $Fe^{2.5+}{}_4S_4$ subcluster, but all include antiferromagnetic coupling between Ni and the adjacent Fe site, Fe1 (Figure S39). While all three BS solutions are plausible models of **$NiFe_4S_4$**, BS-03 is the most probable; BS-01 produces an optimized geometry with larger errors in M–M and M–L distances, while BS-02 is 4.1 kcal/mol higher in energy (Table S8).

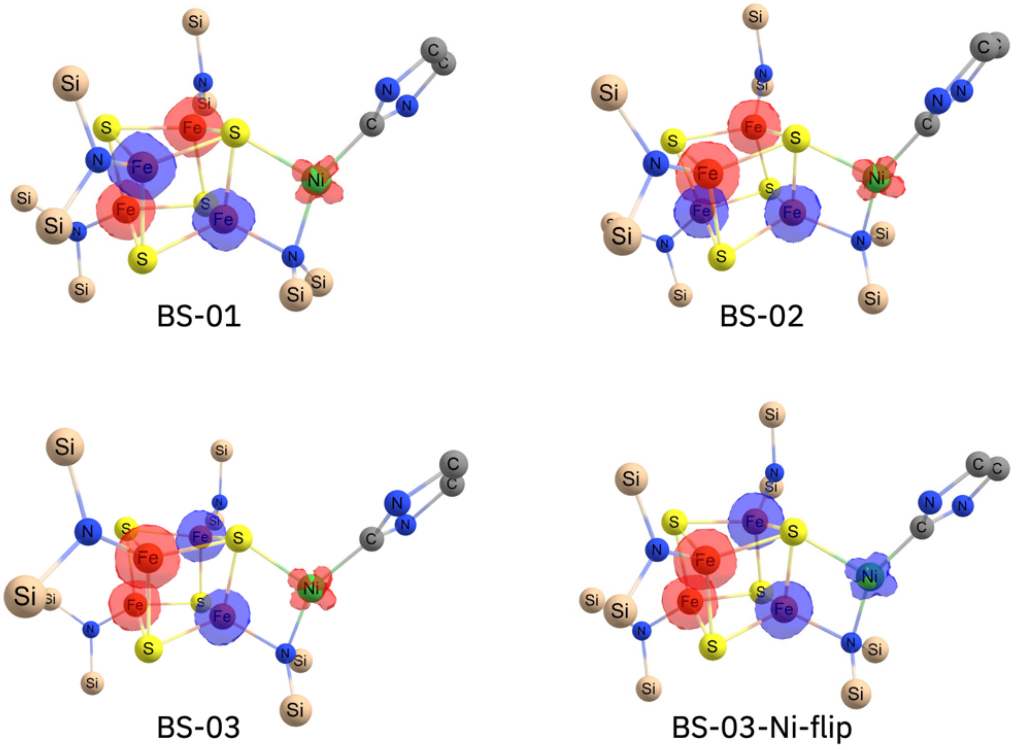

Figure S39. Total spin density plots for the three candidate BS wavefunctions investigated, as well as a BS wavefunction equivalent to BS-03 except for the relative spin at Ni being flipped. Red and blue indicate alpha and beta spin density, respectively. BS-01 and BS-03 are inequivalent because **$NiFe_4S_4$** deviates significantly from C_S symmetry, both in the crystal structure and all DFT-optimized structures. The total spin density of BS-03-Ni-flip has been plotted after multiplication by -1 for ease of visual comparison to BS-03; the ORCA program does not allow for BS DFT calculations with a negative M_S.

Table S8. Relative energies and mean absolute errors (MAEs) in metal-metal and metal-ligand distances versus crystallographic coordinates for structures of **$NiFe_4S_4$** optimized with three different BS topologies.

	Relative Energy (kcal/mol)	M-M MAE (Å)	M-L MAE (Å)
BS-01	0.7	0.082	0.040
BS-02	4.1	0.037	0.025
BS-03	0.0	0.056	0.028

Spin coupling between Ni^{1+} and the $Fe^{2.5+}{}_4S_4$ subcluster was assessed for the BS-02 and BS-03 optimized structures. BS wavefunctions were converged with the same spin topology used in the structure optimization, except for the spin on Ni being flipped to be parallel to that of Fe1 (Figure S39). The Ni-spin-flipped BS wavefunctions were 4.0 and 2.8 kcal/mol higher in energy for BS-02 and BS-03 respectively, indicating significant antiferromagnetic coupling between Ni and Fe1. At the BS-03 geometry, BS wavefunctions were also found with all-parallel [4Fe-4S] subclusters; all BS wavefunctions at this geometry are detailed in Table S9. The BS wavefunction with an all-parallel [4Fe-4S] subcluster and antiparallel Ni^+ has an energy of +31 kcal/mol relative to the BS-03 ground state.

We have provided these BS DFT energy differences to demonstrate that there is significant magnetic interaction between the Ni^{1+} and the [4Fe-4S] cluster at the BS DFT level, and likely also in the actual system. The energy of this interaction is less than that between the mixed-valence pairs within the [4Fe-4S] cluster. We do not calculate a BS DFT Ni-Fe exchange coupling constant for two reasons. First, calculation of an exchange coupling constant for the Ni-Fe interaction would require defining a second subsite spin to interact with the Ni^{1+} spin, which is not straightforward for **$NiFe_4S_4$**. Second and more generally, the Heisenberg exchange model is known to break down severely for Fe-S systems,[28] and so we would not expect to obtain meaningful spin Hamiltonian parameters from BS DFT for this system.

Table S9. Relative energies for different BS solutions of **$NiFe_4S_4$** , all calculated using the BS-03 geometry.

	BS Spin Topology [Fe2+Fe4, Fe1+Fe3, Ni]	BS M_S	Relative Energy (kcal/mol)	Relative Energy (cm^{-1})
BS-03	[α, β, α]	1/2	0.0	0
BS-03-Ni-flip	[β, α, α]	1/2	2.8	973
HS-03	[α, α, β]	17/2	30.7	10735
HS-03-Ni-flip	[α, α, α]	19/2	33.9	11870

DFT Analysis of $Ni_2Fe_3S_4$

$Ni_2Fe_3S_4$ has a total spin $S = 5/2$, determined by magnetometry. The presence of a pre-edge feature in the Ni K-edge XAS indicates significant depopulation of the Ni 3*d* manifold and suggests a local Ni^+ oxidation state. This assignment, together with the total spin, enables assignment of the central Fe (Fe2) as Fe^{3+} and the peripheral Fe (Fe1) as Fe^{2+}. All assignments are supported by the BS DFT analysis. All metal 3*d* orbitals were clearly localized, including a Ni 3*d* SOMO, with some M-M sigma delocalization observed between Fe_C and the peripheral metals (Figure S40). Attempts to converge qualitatively different BS wavefunctions were not fruitful.

In the crystal structure of **$Ni_2Fe_3S_4$**, a Na^+ ion is located 2.819 Å from two of the μ_3-sulfide ions and is additionally ligated by two THF molecules. Inclusion of the $Na(THF)_2$ moiety did not significantly affect geometry optimization, TD-DFT or isomer shift calculations. All properties of **$Ni_2Fe_3S_4$** are reported with the inclusion of $Na(THF)_2$.

As discussed in the section Mössbauer Isomer Shifts from DFT, the isomer shift calculated for Fe1 sites is significantly underestimated, indicating the calculated electron density at the Fe1 nuclei is too low. Differences in isomer shifts are primarily due to differences in 3*d* and 4*s* hybridization and occupation.[29,30] One possible explanation for the Fe1 isomer shift underestimation is that the 3*d* delocalization observed in the Fe1 orbital of Figure S40 is overestimated in the BS wavefunction.

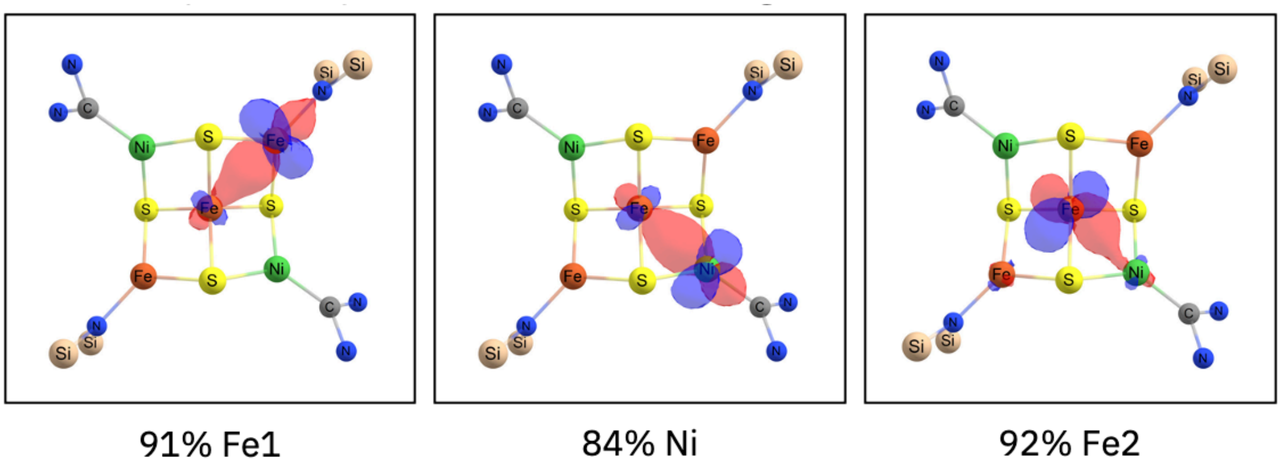

Figure S40. The three localized orbitals of **$Ni_2Fe_3S_4$** with M-M σ interactions between the central Fe (Fe2) and the peripheral metals; the left and middle orbitals are α, while the right orbital is β. The middle orbital is a Ni 3*d* SOMO. σ delocalization from Fe2 to Fe1 was not observed in any Fe2 3*d* orbitals.

Computational X-ray Absorption Spectroscopy

X-ray spectra of **NiFe4S$_4$** and **Ni$_2$Fe$_3$S$_4$** were calculated using TDA-TD-DFT with 400 roots per absorbing atom. Natural transition orbitals and Hirshfeld populations of electron-hole distributions were used to characterize excited states.

Clusters **NiFe$_4$S$_4$** and **Ni$_2$Fe$_3$S$_4$** both have Ni pre-edges at 8330 eV, indicating significant depopulation of the Ni 3*d* orbitals in these clusters compared to the unambiguously d^{10} starting materials. Calculated Ni K-edges of both clusters have first excited states with dominant Ni 3*d* character and are present only in the β manifold, supporting the assignments of Ni^{1+} (Figure S41).

The calculated pre-edge of **Ni$_2$Fe$_3$S$_4$** is shown in Figure S42, with transitions partitioned into those with Fe2 and Fe1 core-holes and those with local or MMCT character. The separation of 0.5 eV between the local 3d transitions of $Fe2^{3+}$ and $Fe1^{2+}$ supports the local oxidation state assignments. The presence of many low-lying charge-transfer transitions and featureful lower-edge in the experimental Fe XAS of **Ni$_2$Fe$_3$S$_4$** would necessitate many peak functions to fit, allowing almost arbitrary fitted peak positions to be obtained. Thus, experimental fits of **Ni$_2$Fe$_3$S$_4$** are not presented.

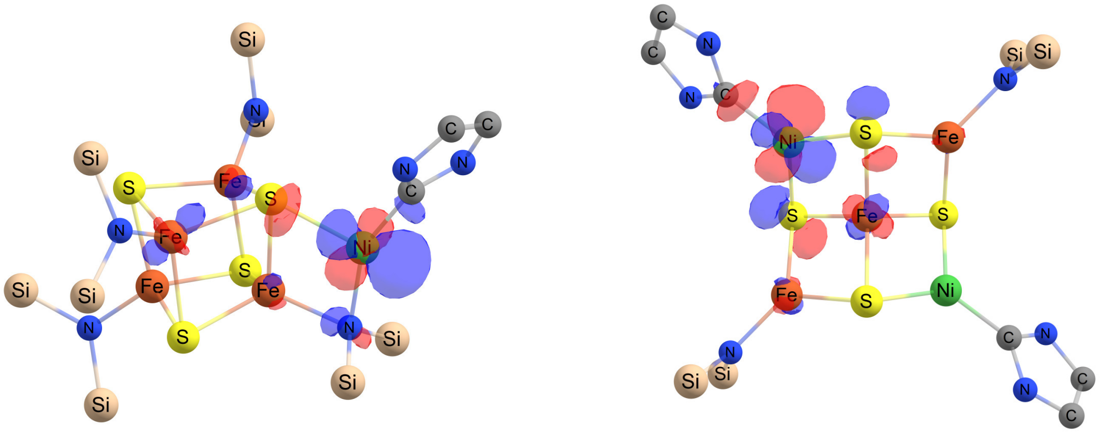

Figure S41. Natural transition orbitals of the first Ni K-edge excited states of **NiFe$_4$S$_4$** (left) and **Ni$_2$Fe$_3$S$_4$** (right). The transitions have primarily local Ni 3*d* character and are present for only for beta spin, supporting the assignment of Ni^+ for both clusters.

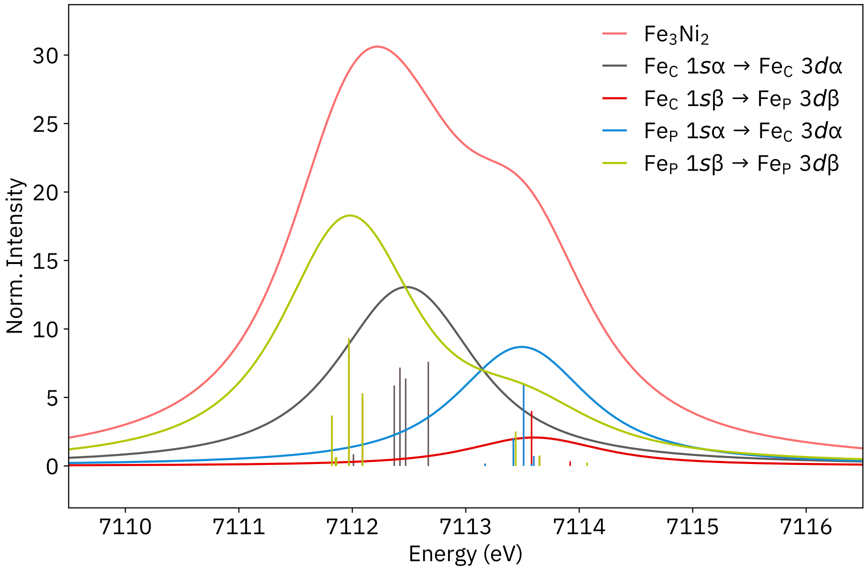

Figure S42. Calculated Fe pre-edge of $\mathbf{Ni_2Fe_3S_4}$ including only the first 31 transitions, with transitions grouped by the character of the transition.

Ni 4p_z Transitions

Ni K-edges were measured for $\mathbf{Ni_2Fe_3S_4}$, $\mathbf{NiFe_4S_4}$ and the Ni^0 reference complex (IPr)Ni(styrene)$_2$ (Figure S43). Pronounced Ni 4p_z peaks are observed at 8335 eV in the two species for which the Ni 4p_z is relatively localized and unmixed with other orbitals, namely the low-lying π* orbital of the N-heterocyclic carbene (NHC). This orbital mixing depends on the rotation angle of the NHC plane relative to the Ni coordination plane (the *interplane angle*). For $\mathbf{NiFe_4S_4}$ and (IPr)Ni(styrene)$_2$, the interplane angles are 60° and 67°, an orientation that mostly prohibits mixing between the Ni 4p_z and NHC π* orbitals. For $\mathbf{Ni_2Fe_3S_4}$, the interplane angle is 10°, which allows mixing between these orbitals. For all three species, the angle between the NHC plane and Ni coordination plane is sterically determined by the large organic groups on the NHCs.

The relationship between the interplane angle and prominence of the Ni 4p_z feature was demonstrated computationally using the hypothetical complex [(1,3-diphenyl-imidazol-2-ylidene)Ni(SMe)$_2$]$^-$ (Figure S44). This small complex was chosen to allow for free modification of the interplane angle without steric clashes. When the interplane angle is large and mixing between the Ni 4p_z and NHC π* is not possible, a prominent feature is found at 8335 eV resulting from intense, local Ni 4p_z excitations. At smaller interplane angles, more orbital mixing occurs, and the 8335-eV feature is less prominent; instead, the Ni 4p_z mixes with higher-energy NHC orbitals, increasing transition intensity around 8338 eV. While this second spectral effect may be observable in $\mathbf{Ni_2Fe_3S_4}$, its magnitude is overestimated in the TD-DFT calculation due to the

diminishing capability of the method to accurately capture excited states with higher energies and greater charge-transfer character.

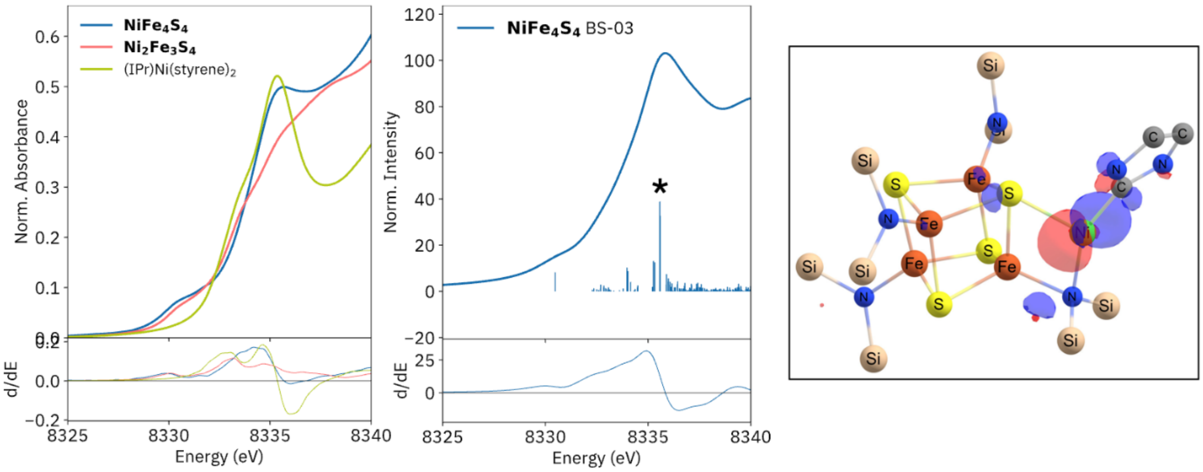

Figure S43. Experimental Ni pre-edges for $\mathbf{NiFe_4S_4}$, $\mathbf{Ni_2Fe_3S_4}$ and IPrNi(styrene)$_2$ (left), Ni pre-edge of $\mathbf{NiFe_4S_4}$ calculated from the BS-03 wavefunction (middle) and the natural transition orbital for the Ni 4p_z excited state marked with a star (right); the z axis is defined as normal to the Ni coordination plane.

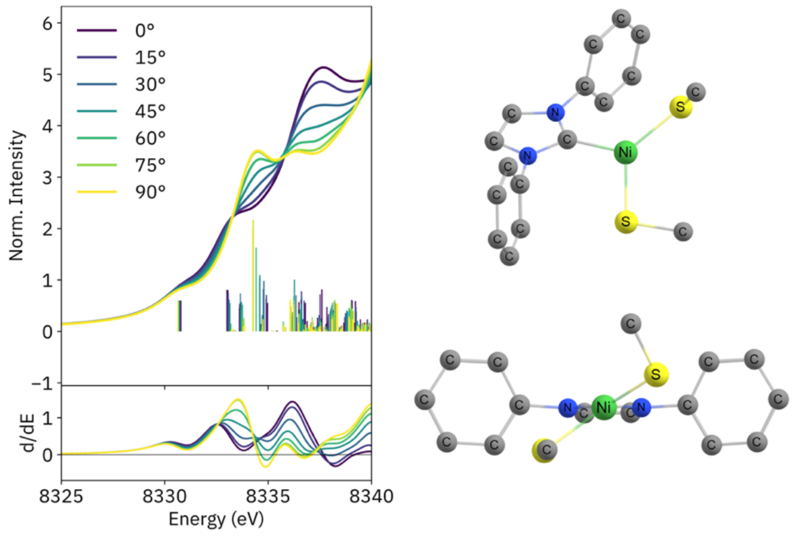

Figure S44. Ni K-edges for the hypothetical complex [(1,3-diphenyl-imidazol-2-ylidene)Ni(SMe)$_2$]$^-$ calculated from structures with various interplane angles between the Ni coordination plane and the NHC plane (left); two views of the hypothetical complex in the 30° structure, with hydrogen atoms omitted for clarity (right).

Mössbauer Isomer Shifts from DFT

Mössbauer isomer shifts (δ) are linearly dependent on the electron density ρ_0 at the ^{57}Fe nucleus. The calculation of isomer shifts from DFT (or other) wavefunctions is well-established using the equation $\delta = \alpha + \beta(\rho_0 - C)$, where α, β and *C* are constants obtained by linear regression of a reference dataset.[29,31] The parameters used here (α = -0.309785357, β = 0.325438551, *C* = 13785) were calculated by Ragnar Bjornsson for the present DFT protocol and the new grid scheme in ORCA 5 following published procedures.[32] A large reference dataset was used, combining the datasets of Römelt and coworkers[33] and Sandala and coworkers, with an emphasis on sulfur-ligated species, including 2Fe2S and 4Fe4S clusters.[34] Second-order doppler (SOD) shifts for spectra collected at higher temperatures were adjusted to 4 K using a simple linear correction of 0.00040568 mm/s•K that may have introduced individual absolute errors 0.01–0.02 mm/s.[29,34] This same parameterization was used in a recent study of heterometallic iron-sulfur clusters by the present authors.[35]

The calculated δ values for **$Ni_2Fe_3S_4$** (average 0.38 mm/s, Fe2 0.36 mm/s, Fe1 0.39 mm/s) deviate considerably from the δ values obtained from fits of the experimental spectrum (one-site fit 0.49 mm/s, two-site fit 0.34 (33%) and 0.52 (67%) mm/s). The quality of calculated δ values is sensitive to the choice of reference dataset; the present parameterization performed well for **$NiFe_4S_4$** as well as two other

heterometallic clusters (errors of -0.02, -0.01 and -0.03 mm/s; errors of -0.01 to -0.02 mm/s are expected from the lack of SOD correction to the experimental data, which were collected at 77 K[29]). The error in the calculated δ values of **$Ni_2Fe_3S_4$** indicates that the BS wavefunction underestimates the electron density at the iron nuclei, in particular the Fe1 sites. This may be a result of overestimation of delocalization of Fe1 *d* electrons onto Fe2 (see section DFT Analysis of **$Ni_2Fe_3S_4$**). We do not believe this shortcoming of the BS DFT casts doubt on the qualitative description of the electronic structure of **$Ni_2Fe_3S_4$** deduced from experiment and supported by the localized orbital analysis, i.e., the assignment of local oxidation states and spin couplings.

References

(1) Zhong, H.; Egger, D. T.; Gasser, V. C. M.; Finkelstein, P.; Keim, L.; Seidel, M. Z.; Trapp, N.; Morandi, B. Skeletal Metalation of Lactams through a Carbonyl-to-Nickel-Exchange Logic. *Nat. Commun.* **2023**, *14* (1), 5273. https://doi.org/10.1038/s41467-023-40979-3.

(2) Wu, J.; Faller, J. W.; Hazari, N.; Schmeier, T. J. Stoichiometric and Catalytic Reactions of Thermally Stable Nickel(0) NHC Complexes. *Organometallics* **2012**, *31* (3), 806–809. https://doi.org/10.1021/om300045t.

(3) Sharp, C. R.; Duncan, J. S.; Lee, S. C. $[Fe_4S_4]^q$ Cubane Clusters (q = 4+, 3+, 2+) with Terminal Amide Ligands. *Inorg. Chem.* **2010**, *49* (14), 6697–6705. https://doi.org/10.1021/ic100742c.

(4) Ohki, Y.; Sunada, Y.; Tatsumi, K. Synthesis of [2Fe–2S] and [4Fe–4S] Clusters Having Terminal Amide Ligands from an Iron(II) Amide Complex. *Chem. Lett.* **2005**, *34* (2), 172–173. https://doi.org/10.1246/cl.2005.172.

(5) Kern, R. J. Tetrahydrofuran Complexes of Transition Metal Chlorides. *J. Inorg. Nucl. Chem.* **1962**, *24* (9), 1105–1109. https://doi.org/10.1016/0022-1902(62)80255-3.

(6) Beattie, D. D.; Lascoumettes, G.; Kennepohl, P.; Love, J. A.; Schafer, L. L. Disproportionation Reactions of an Organometallic Ni(I) Amidate Complex: Scope and Mechanistic Investigations. *Organometallics* **2018**, *37* (9), 1392–1399. https://doi.org/10.1021/acs.organomet.8b00074.

(7) Stoll, S.; Schweiger, A. EasySpin, a Comprehensive Software Package for Spectral Simulation and Analysis in EPR. *J. Magn. Reson.* **2006**, *178* (1), 42–55. https://doi.org/10.1016/j.jmr.2005.08.013.

(8) Bain, G. A.; Berry, J. F. Diamagnetic Corrections and Pascal's Constants. *J. Chem. Educ.* **2008**, *85* (4), 532. https://doi.org/10.1021/ed085p532.

(9) Azuah, R. T.; Kneller, L. R.; Qiu, Y.; Tregenna-Piggott, P. L. W.; Brown, C. M.; Copley, J. R. D.; Dimeo, R. M. DAVE: A Comprehensive Software Suite for the Reduction, Visualization, and Analysis of Low Energy Neutron Spectroscopic Data. *J. Res. Natl. Inst. Stand. Technol.* **2009**, *114* (6), 341–358. https://doi.org/10.6028/jres.114.025.

(10) Kahn, O. *Molecular Magnetism*; VCH: New York, NY, 1993.

(11) Chipman, J. A.; Berry, J. F. Paramagnetic Metal–Metal Bonded Heterometallic Complexes. *Chem. Rev.* **2020**, *120* (5), 2409–2447. https://doi.org/10.1021/acs.chemrev.9b00540.

(12) Newville, M. Larch: An Analysis Package for XAFS and Related Spectroscopies. *J. Phys.: Conf. Ser.* **2013**, *430*, 012007. https://doi.org/10.1088/1742-6596/430/1/012007.

(13) Westre, T. E.; Kennepohl, P.; DeWitt, J. G.; Hedman, B.; Hodgson, K. O.; Solomon, E. I. A Multiplet Analysis of Fe K-Edge 1s → 3d Pre-Edge Features of Iron Complexes. *J. Am. Chem. Soc.* **1997**, *119* (27), 6297–6314. https://doi.org/10.1021/ja964352a.

(14) Hugenbruch, S.; Shafaat, H. S.; Krämer, T.; Delgado-Jaime, M. U.; Weber, K.; Neese, F.; Lubitz, W.; Debeer, S. In Search of Metal Hydrides: An X-Ray Absorption and Emission Study of [NiFe] Hydrogenase Model Complexes. *Phys. Chem. Chem. Phys.* **2016**, *18* (16), 10688–10699. https://doi.org/10.1039/c5cp07293j.

(15) Neese, F. Software Update: The ORCA Program System—Version 5.0. *WIREs Comput. Molec. Sci.* **2021**, e1606. https://doi.org/10.1002/wcms.1606.

(16) Weigend, F.; Ahlrichs, R. Balanced Basis Sets of Split Valence, Triple Zeta Valence and Quadruple Zeta Valence Quality for H to Rn: Design and Assessment of Accuracy. *Phys. Chem. Chem. Phys.* **2005**, *7*, 3297–3305.

(17) Tao, J.; Perdew, J. P.; Staroverov, V. N.; Scuseria, G. E. Climbing the Density Functional Ladder: Nonempirical Meta-Generalized Gradient Approximation Designed for Molecules and Solids. *Phys. Rev. Lett.* **2003**, *91* (14), 146401. https://doi.org/10.1103/PhysRevLett.91.146401.

(18) van Wüllen, C. Molecular Density Functional Calculations in the Regular Relativistic Approximation: Method, Application to Coinage Metal Diatomics, Hydrides, Fluorides and Chlorides, and Comparison with First-Order Relativistic Calculations. *J. Chem. Phys.* **1998**, *109* (2), 392–399.

(19) Lenthe, E. van; Baerends, E. J.; Snijders, J. G. Relativistic Regular Two-component Hamiltonians. *J. Chem. Phys.* **1993**, *99* (6), 4597–4610. https://doi.org/10.1063/1.466059.

(20) Grimme, S.; Antony, J.; Ehrlich, S.; Krieg, H. A Consistent and Accurate Ab Initio Parametrization of Density Functional Dispersion Correction (DFT-D) for the 94 Elements H-Pu. *J. Chem. Phys.* **2010**, *132* (15). https://doi.org/10.1063/1.3382344.

(21) Grimme, S.; Ehrlich, S.; Goerigk, L. Effect of the Damping Function in Dispersion Corrected Density Functional Theory. *J. Comput. Chem.* **2011**, *32* (7), 1545–1614. https://doi.org/10.1002/jcc.

(22) Barone, V.; Cossi, M. Quantum Calculation of Molecular Energies and Energy Gradients in Solution by a Conductor Solvent Model. *J. Phys. Chem. A* **1998**, *102* (11), 1995–2001. https://doi.org/10.1021/jp9716997.

(23) Neese, F.; Wennmohs, F.; Hansen, A.; Becker, U. Efficient, Approximate and Parallel Hartree–Fock and Hybrid DFT Calculations. A 'Chain-of-Spheres' Algorithm for the Hartree–Fock Exchange. *J. Chem. Phys.* **2009**, *356* (1), 98–109. https://doi.org/10.1016/j.chemphys.2008.10.036.

(24) Izsák, R.; Neese, F. An Overlap Fitted Chain of Spheres Exchange Method. *J. Chem. Phys.* **2011**, *135* (14), 144105. https://doi.org/10.1063/1.3646921.

(25) Weigend, F. Accurate Coulomb-Fitting Basis Sets for H to Rn. *Phys. Chem. Chem. Phys.* **2006**, *8* (9), 1057–1065. https://doi.org/10.1039/b515623h.

(26) Pipek, J.; Mezey, P. G. A Fast Intrinsic Localization Procedure Applicable for Ab Initio and Semiempirical Linear Combination of Atomic Orbital Wave Functions. *J. Chem. Phys.* **1989**, *90* (9), 4916–4926. https://doi.org/10.1063/1.456588.

(27) Hirshfeld, F. L. Bonded-Atom Fragments for Describing Molecular Charge Densities. *Theoret. Chim. Acta* **1977**, *44* (2), 129–138. https://doi.org/10.1007/BF00549096.

(28) Sharma, S.; Sivalingam, K.; Neese, F.; Chan, G. K.-L. Low-Energy Spectrum of Iron–Sulfur Clusters Directly from Many-Particle Quantum Mechanics. *Nature Chem.* **2014**, *6* (10), 927–933. https://doi.org/10.1038/nchem.2041.

(29) Gütlich, P.; Bill, E.; Trautwein, A. X. *Mössbauer Spectroscopy and Transition Metal Chemistry: Fundamentals and Applications*; Springer-Verlag: Berlin Heidelberg, 2011. https://doi.org/10.1007/978-3-540-88428-6.

(30) Neese, F. Prediction and Interpretation of the ^{57}Fe Isomer Shift in Mössbauer Spectra by Density Functional Theory. *Inorg. Chim. Acta* **2002**, *337*, 181–192. https://doi.org/10.1016/S0020-1693(02)01031-9.

(31) Neese, F. Prediction of Molecular Properties and Molecular Spectroscopy with Density Functional Theory: From Fundamental Theory to Exchange-Coupling. *Coord. Chem. Rev.* **2009**, *253* (5–6), 526–563. https://doi.org/10.1016/j.ccr.2008.05.014.

(32) Bjornsson, R.; Neese, F.; DeBeer, S. Revisiting the Mössbauer Isomer Shifts of the FeMoco Cluster of Nitrogenase and the Cofactor Charge. *Inorg. Chem.* **2017**, *56* (3), 1470–1477. https://doi.org/10.1021/acs.inorgchem.6b02540.

(33) Römelt, M.; Ye, S.; Neese, F. Calibration of Modern Density Functional Theory Methods for the Prediction of ^{57}Fe Mössbauer Isomer Shifts: Meta-GGA and Double-Hybrid Functionals. *Inorg. Chem.* **2009**, *48* (3), 784–785. https://doi.org/10.1021/ic801535v.

(34) Sandala, G. M.; Hopmann, K. H.; Ghosh, A.; Noodleman, L. Calibration of DFT Functionals for the Prediction of ^{57}Fe Mössbauer Spectral Parameters in Iron–Nitrosyl and Iron–Sulfur Complexes: Accurate Geometries Prove Essential. *J. Chem. Theory Comput.* **2011**, *7* (10), 3232–3247. https://doi.org/10.1021/ct200187d.

(35) Wilson, D. W. N.; Fataftah, M. S.; Mathe, Z.; Mercado, B. Q.; DeBeer, S.; Holland, P. L. Three-Coordinate Nickel and Metal–Metal Interactions in a Heterometallic Iron–Sulfur Cluster. *J. Am. Chem. Soc.* **2024**, *146* (6), 4013–4025. https://doi.org/10.1021/jacs.3c12157.
